# Carbon stock quantification and climate mitigation potential of a tropical moist forest in Ethiopia

**DOI:** 10.1371/journal.pone.0316886

**Published:** 2025-01-24

**Authors:** Alemayehu K. Shembo, Teshome Soromessa, Sebsebe Demissew, Addisie Geremew, Ram L. Ray, Laura Carson

**Affiliations:** 1 Cooperative Agricultural Research Center, College of Agriculture, Food and Natural Resources, Prairie View A&M University, Prairie View, TX, United States of America; 2 Department of Plant Biology and Biodiversity Management, Addis Ababa University, Addis Ababa, Ethiopia; 3 Center for Environmental Science, Addis Ababa University, Addis Ababa, Ethiopia; Western Carolina University, UNITED STATES OF AMERICA

## Abstract

The significance of forests in absorbing and storing carbon plays a crucial role in international greenhouse gas policies outlined by the United Nations Framework Convention for Climate Change (UNFCC). This study was conducted in a typical tropical moist forest of Ethiopia to assess its carbon stock, a critical issue in climate policy. The study domain was divided into six strata using elevation criteria. Ninety sample plots were used to gather relevant data from all carbon pools (above-ground biomass, below-ground biomass, litter, herbs, lying dead woods, and soils) following the standard operating procedure. ANOVA, post hoc analysis and correlation tests were used to analyze the collected data. The finding revealed that carbon stock in Sele-Nono forest varies not only within its carbon pools but also across environmental factors. Moreover, the study indicated that soil, above-ground biomass, and lying dead woods store the majority of the carbon. The forest stored 284.81±107.81 tons of carbon per hectare, which is equivalent to absorbing 157.12 Megatons of CO_2_ from the atmosphere. This highlights the critical role of the forest in mitigating climate change on a global scale. The finding from this study encourages policymakers to rigorously focus on forest conservation as a strategy for sustainable climate mitigation. Moreover, conserving forests through strengthening UN initiatives like REDD+ is imperative to prevent potential emissions from land use changes, such as deforestation or degradation.

## Introduction

The United Nations Framework Convention on Climate Change (UNFCCC) characterizes climate change as ’alterations in the global atmosphere’s composition that can be directly or indirectly attributed to human activities [1]. At present, there is undeniable evidence that the global climate is changing. This includes noticeable shifts in temperature and precipitation patterns, an increase in extreme weather events, and rising sea levels, which are just a few witnesses. However, some individuals deny the existence of climate change, considering it a conspiracy [2,3], which is completely untrue. As per intensive research reports, alterations in climate are attributed to the rise in concentrations of anthropogenic greenhouse gases (GHG), primarily carbon dioxide, in the atmosphere [4]. This issue has become a major concern for scientists, political leaders, and nations.

Scientific projections indicate that preventing hazardous climate change, characterized by a global average temperature exceeding two degrees Celsius, requires stabilizing atmospheric greenhouse gas (GHG) concentrations. This involves reducing the current concentration of CO_2_ from 420 ppm to 350 ppm by the year 2100, if not earlier [5]. This is the ultimate objective of UNFCC, as expressed in its Article 2 [1,6]. One of the highly operative and cost effective approaches for mitigating atmospheric greenhouse gas concentrations is conserving forest ecosystems [7,8].

In recent decades, the amount of carbon stored in forest biomass has gained particular focus, as suggested by the UN Framework Convention on Climate Change [9]. It is reported that countries, particularly those of Annex I, are requested to objectively compute and report the amount of carbon dioxide (CO_2_) removed due to their forest stands [10]. Since then, the interest in accurately assessing forest biomass to determine the increase in carbon storage (reflecting carbon removal from the atmosphere) or decrement (indicative of carbon emissions from a forest) has become increasingly important.

Among the various forest regions worldwide, tropical forests have gained international recognition as an important center to offset the effect of climate change. This acknowledgment is attributed to their exceptional ability to sequester carbon and the substantial emissions that would result if they were subjected to destruction [11]. In consensus with this perspective, researchers have estimated the potential emission of 1.3 billion tons (1.3Gt) to 1.6 billion tons (1.6 Gt) of CO_2_ annually upon the destruction of tropical forests [12,13]. These researchers agreed that the carbon stock of tropical forests accounts for approximately one-fifth of the total global carbon stock. Moreover, they noted that the carbon stored in the vegetation component of tropical forests represents half of the above-ground carbon stored in the vegetation of all other biomes. Consequently, owing to their substantial carbon stock, tropical forests are increasingly recognized as a means for mitigating climate change.

Many developed countries worldwide have engaged in research focused on their forests, aiming to understand the forests’ contributions to addressing climate change [14]. Some African countries have also attempted to assess the role of their forests in reducing GHGs from the atmosphere [15,16] and benefited from carbon credit through REDD+ program [16]. Ethiopia, as a tropical country in Africa with significant forest carbon resources [17,18] and land with relatively good forest coverage (15%), should have benefited much [19]. However, data regarding the carbon storage in Ethiopian forests was insufficient, and there has been minimal exploration of forest-specific studies, except for a few pioneering efforts [20–34] and some gross estimation [35,36].

Although the major dense forests in Ethiopia are located in the humid highlands of the Southwest (SW) regions [37,38], their carbon stock has not been accurately estimated and quantified, except for a few attempts [39–41]. Therefore, this research was carried out in the Sele-Nono moist forest, a region of the moist southwest forest in Ethiopia, to determine the current carbon stock of the forest, which can serve as a benchmark value.

The primary goal of this research was to assess the role of Sele-Nono forests in climate change mitigation. The specific objectives of this research were to (i) estimate the current carbon stock of the forest using field-based data, (ii) determine which carbon pools in Sele-Nono Forest hold the majority of the carbon, and (iii) explore whether environmental conditions within the study area affect the spatial variation of carbon stocks.

## Materials and methods

### Study area

**Fig 1:**
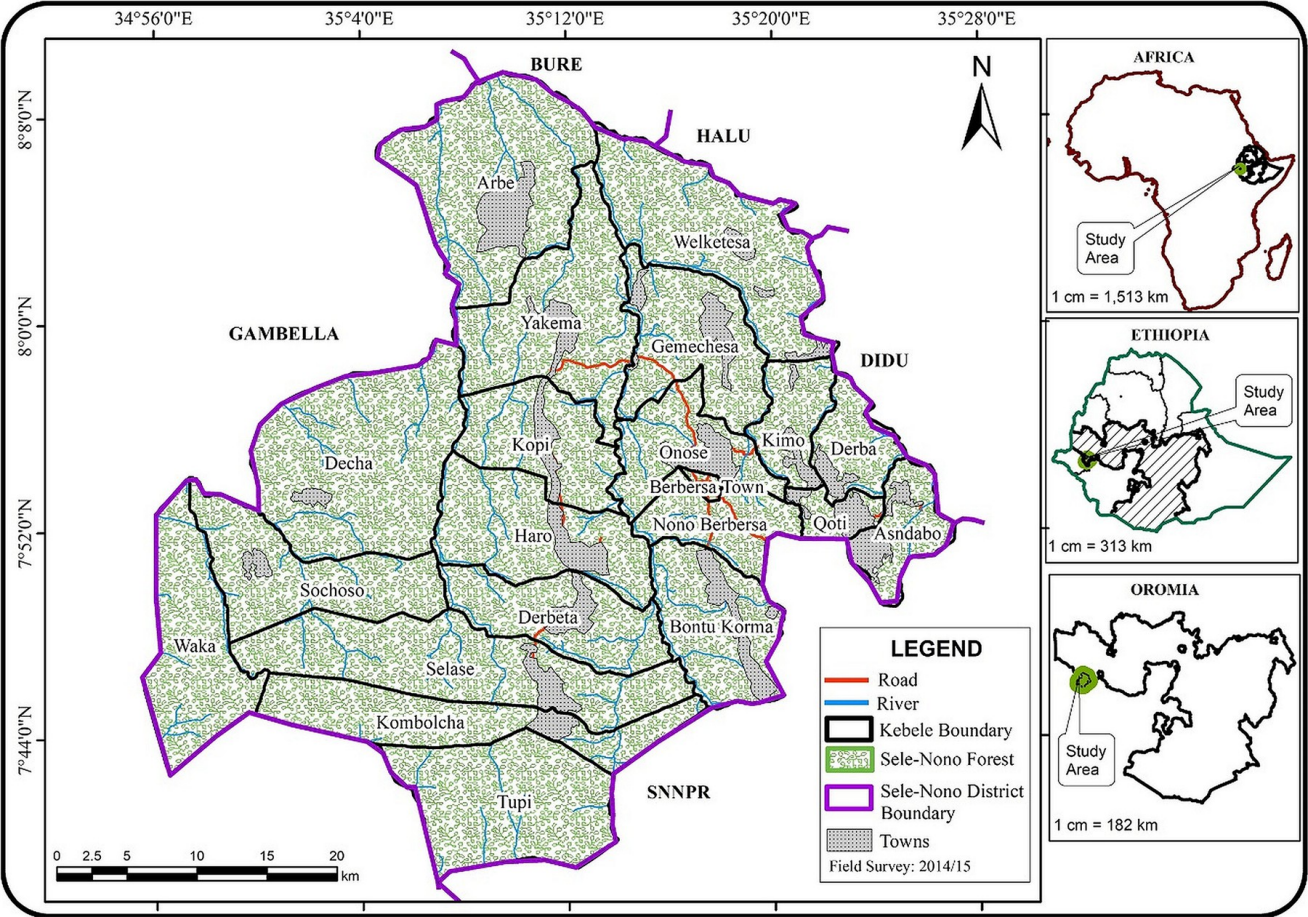
Map showing the location of the study domain.

This research was conducted in Sele-Nono Forest, part of the country’s national forest priority area (NFPA) and located in Sele-Nono District of Oromia Regional State of Ethiopia. The forest is approximately 100 km and 50 km away from Metu town and Masha town, respectively (the nearest major towns), and it is about 700 km from Addis Ababa, the capital of Ethiopia, en route to the Gambella region. Geographically, Sele-Nono Forest is situated between 7°48” -7°57” longitude and 35°12” - 35°17” latitude. It is bordered on the Southwest by the Gambela region, North by Bure, Northeast by Halu, and Southeast by the Southern Nations, Nationalities and Peoples Region (SNNPR) (Fig 1).

### Vegetation and soil type of the forest

A previous study by Kefalew et al. [42] found that the Sele-Nono forest is intact and covers over 91 percent of the district. This forest includes a transitional rainforest part with species such as *Pouteria atissima*, *Anthocleista schweinfurthii*, *Manilkara butugi*, *Morus mesozygia*, *Strychnos mitis*, *Trichilia dregeana*, and *Trilepisium madascariense*. In contrast, the Afromontane forest area is primarily composed of *Pouteria* (*Aningeria*)-*adolfi-friederici*, *Syzygium guineense*, *Olea welwitschii*, *Schefflera abyssinica*, *Croton macrostachyus*, *Ilex mitis*, and others [43]. Despite its pristine condition, the forest faces threats from selective logging and thinning. Studies have also shown that the forest is in a mature secondary successional stage. Additionally, the soils are predominantly Nitosols with a pH range of 6.4 to 6.6, as reported by the same author [42].

### Climate

**Fig 2:**
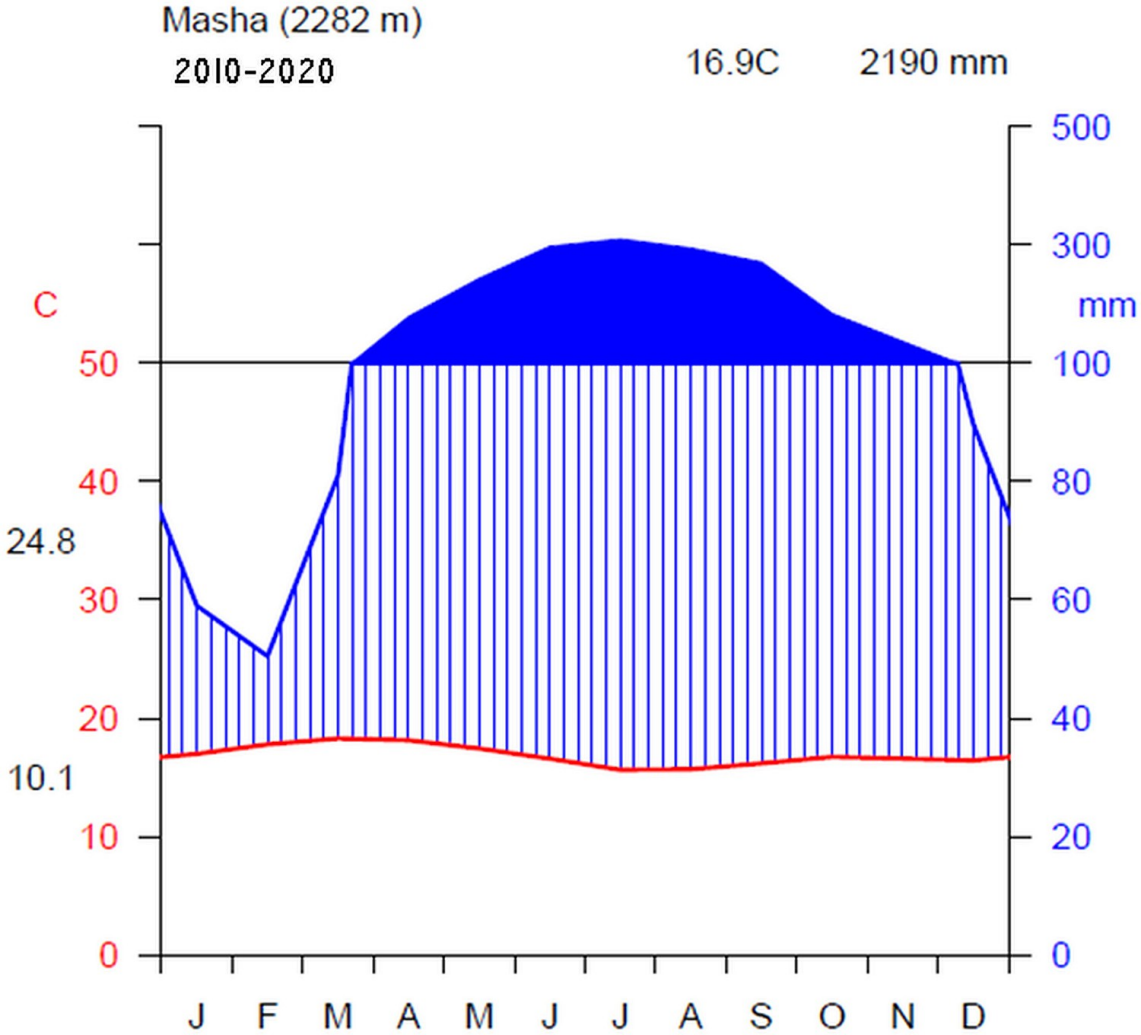
Climadiagram of Sele-Nono forest showing rainfall distribution and temperature variation from 2010–2020 at Masha. Data source: National Meteorological Agency of Ethiopia [44].(Note that the heading on the climate diagram indicates the elevation of the climate station, along with the mean annual temperature and precipitation for the year plotted. The blue curve represents precipitation, while the red curve denotes temperature. The daily maximum average temperature of the hottest month and the daily minimum average temperature of the coldest month are labeled in black on the left margin of the diagram. The solid blue area above the black horizontal line indicates the wet period of the study area, and the vertical blue stripes show the humid period).

The study area experiences eight rainy months, spanning from March to October, with a well-distributed rainfall pattern (Fig 2). Rainfall data obtained from the Masha meteorological station, the nearest station (50 Km) to Sele-Nono forest, reveals that the area receives notably high annual rainfall, reaching up to 2200 mm in certain peak years. The daily maximum average temperature of the hottest month and daily minimum average temperature of the coldest month in the area were recorded at 24.8°C and 10.1°C, respectively. The mean annual temperature is 16.9°C, showing slight variations yearly (Fig 2). Rainfall patterns show lower precipitation in January and February, gradually increasing to a peak in July and August then decreasing in November and December.

### Research design

#### (i) Delineation and stratification of the study area

**Fig 3:**
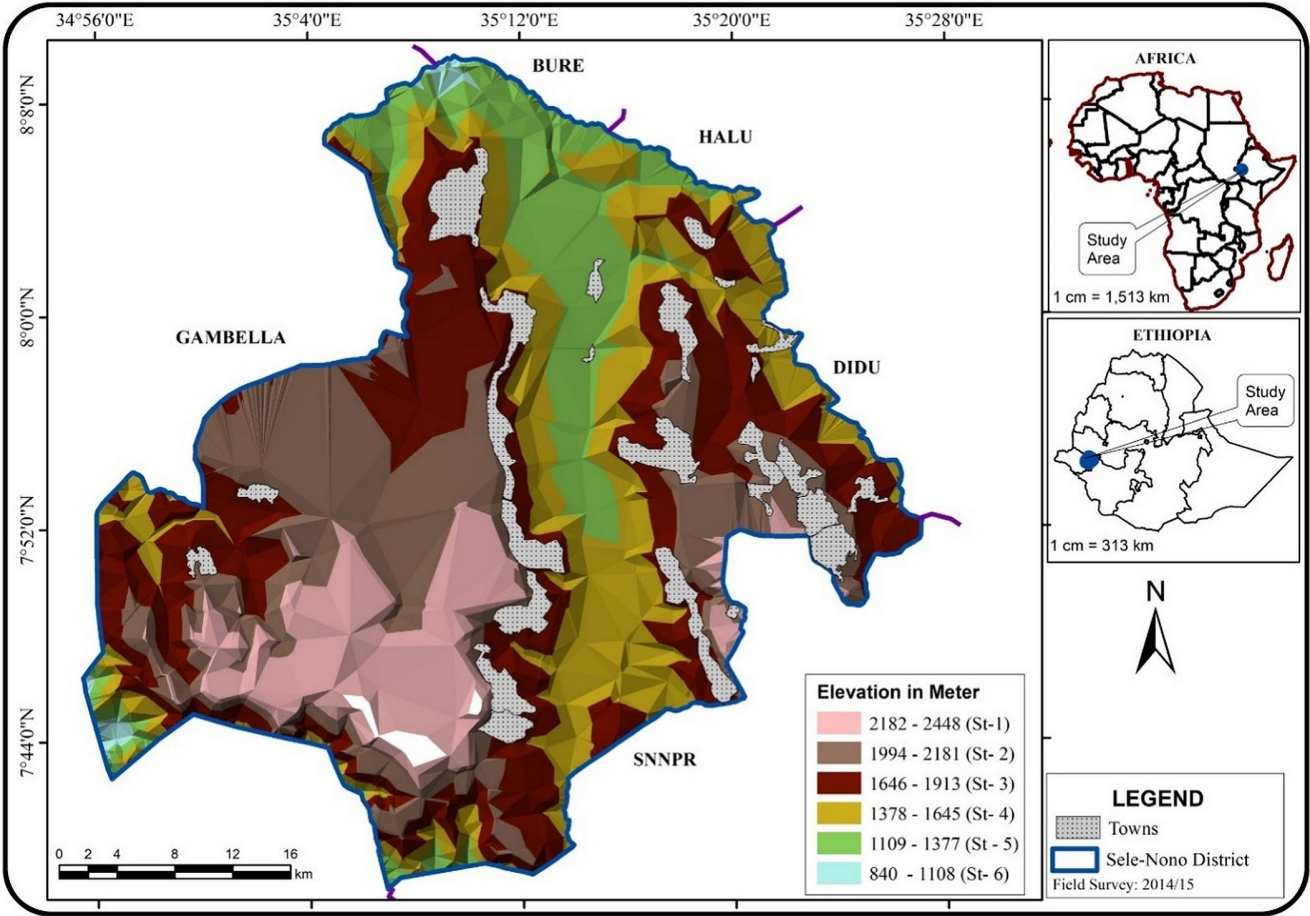
Delineation and stratification of the study area.

The spatial boundaries of the study forest were clearly defined and recognized from the start. This ensured that carbon pool measurements were taken only within the specified area, avoiding any mixing with vegetation from neighboring forest edges. This approach enhances the accuracy of carbon stock estimates in the designated areas. The boundary delineation was achieved by utilizing the clipping technique from the Ethio shape file and Google Earth images. The area of the study forest was calculated at 150,325.27 hectares by capturing and measuring the image of the study area on Google Earth. Since our study area is heterogeneous, we stratified it based on elevation segmentation using 275 m elevation differences to obtain relative homogenous vegetation units and reduce uncertainties in subsequent carbon stock estimations. Accordingly, the study forest was categorized into six strata (Fig 3) following the maximum recommended strata number suggested by MacDicken [45].

#### (ii) Sampling techniques

Following the stratification of the study area, sample plots were allocated proportionally to each stratum (Table 1). The number of plots in each stratum ranges from 1 to 22, depending on the stratum’s size, resulting in a total of 90 sampling plots.

**Table 1:** Number of sample plots proportionally allocated to each stratum in Sele-Nono forest.

Strata (St.) No.	Elevation range for each stratum (m, asl)	Total area (forest & village) of each stratum (Ha)	Total village areas in each stratum (Ha)	Net forest areas in each stratum (Ha)	Proportion of forest areas in each stratum (%)	Total number of plots allocated to each stratum
**Stratum 1**	2182–2448	18490	117	18373	12	11
**Stratum 2**	1994–2181	42025	5279	36746	24	22
**Stratum 3**	1646–1993	45674	8926	36746	24	22
**Stratum 4**	1378–1645	33827	422	33405	22	20
**Stratum 5**	1109–1377	23637	254	23383	15	14
**Stratum 6**	840–1108	1670	0	1670	1	1
**Total**	**840–2448**	**165,325**	**14,999**	**150,325**	**100**	**90**

**Fig 4:**
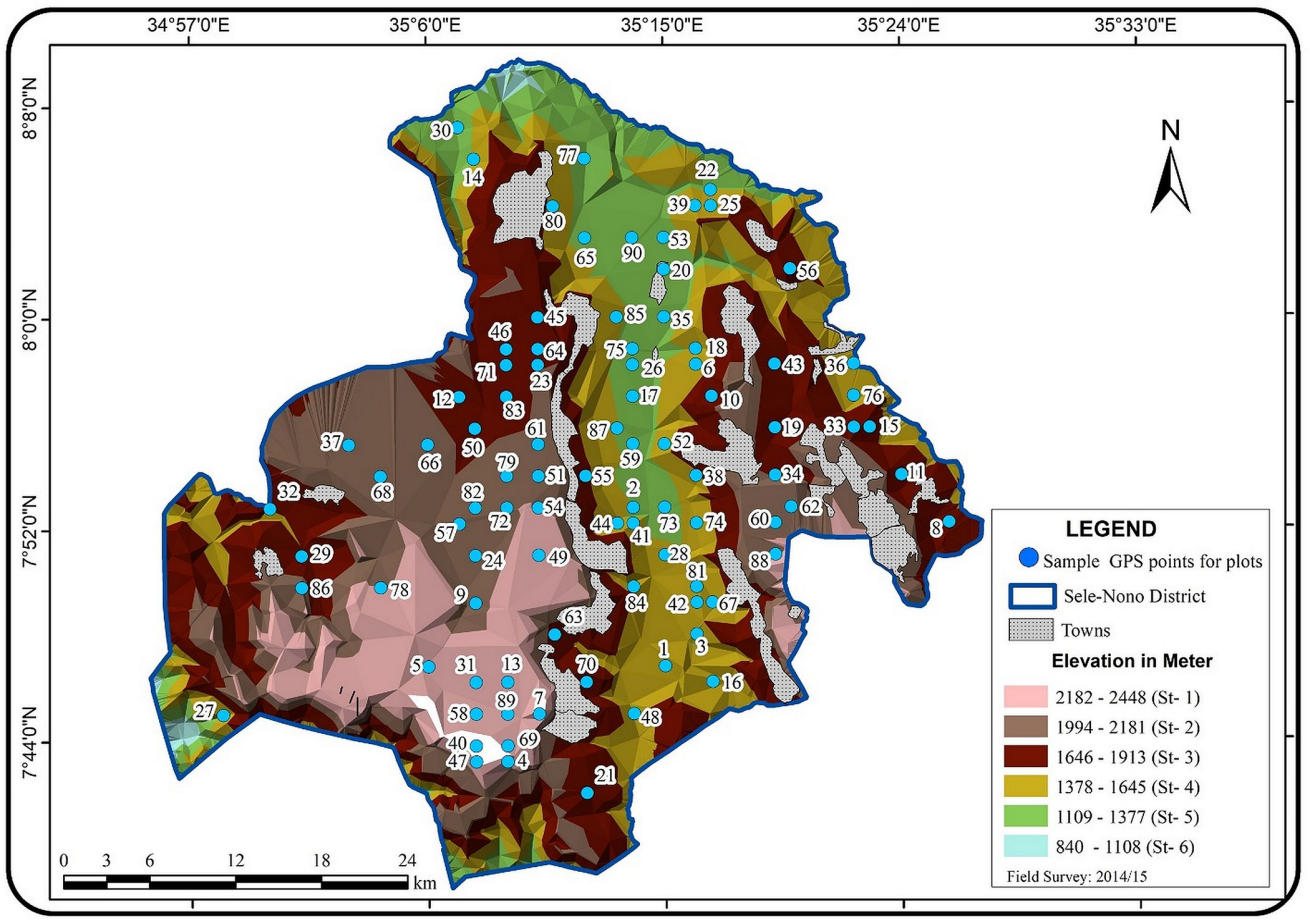
Location of sample plots in the study area.

The sample plots were allocated by choosing geographical grids on the map of the area before going to the field. The grids were made by dividing the map of the study forest in latitude and longitude coordinates using a one-minute (1’) grid-scale, which is equivalent to a 1.85 km actual distance on the ground. Then, some of the intersection points of the grids in each stratum were randomly chosen as a sample point (sample plot); and plot numbers were given randomly to each of the sampling points using computational technique (S1 Appendix). Those plot points on the map (Fig 4) were transferred to the ground of the actual study area using GPS navigation after ridding to the nearest town. Sampling was conducted after checking any natural or semi-natural vegetation category in the stratum. Agricultural fields and plantations were not sampled.

### Plot design and data collection

A permanent plot of size 35m x 35m (1225m^2^ (~0.1225 ha)) was randomly laid out at each of the previously determined sample plot locations. Each of the main plots was nested into three sections: 25 m x 25 m (for collecting data on medium trees with a diameter at breast height (DBH) of 20 cm to 50 cm), 7 m x 7 m (for collecting data on small trees and lianas with a DBH of 5 cm to 20 cm), and 3 m x 3 m (for counting saplings, i.e., trees with a DBH less than 5 cm) (S3 Appendix). Each plot in the nest was considered a separate plot. Such plot design was used in this study since it aligns with the recommended design [41,45,46] for conducting carbon stock assessments in a natural forest. Three environmental data (Altitude, slope, and disturbance) were collected following Kefalew et al. [42]. These environmental factors were considered since they were proven to be influential factors in structuring the vegetation of Sele-Nono forest [42].

Moreover, a 5 m x 5 m subplot was designed in the middle of the 25 m x 25 m nested plot to collect canopy diameters and heights of shrubs, which are relevant data to quantify biomass. In addition, soil samples for carbon analysis were surveyed in five 1m^2^ quadrats within the 25 m x 25 m (625 m^2^) nested plots; four of which were arranged at the four corners, and one was at the center of the 25 m^2^ subplot used for shrub data (S4 Appendix). A summary of the plot size used in the study area is shown below (Table 2).

**Table 2:** Nested plot sizes and measured above-ground biomass components.

Plot size (0% slope)	Biomass pool	DBH range	Remark
1m x 1m	Litter & herbs	………	Also used for soil samples
3 m X 3 m	Sapling	< 5 cm	For counting saplings
5 m X 5 m	Shrubs	………….	
7 m X 7 m	Small trees & Lianas	5 cm -20 cm	
	
25 m X 25 m	Medium trees	20 cm-50 cm	
35 m X 35 m	Big trees	>50 cm	

On top of this, four 1m^2^ plots (1m x 1m) radiating 100 paces away from the center of the main 35 m x 35 m plot were used to gather data for biomass estimation of herbs and litter (S5 Appendix). The positions of these four plots were randomly determined by following the direction of the ’second hand’ of a watch, as suggested by Walker *et al*. [46].

### Data

The primary data were obtained from field measurements to estimate carbon stock of the study forest. Moreover, environmental factors that influence the composition of the forest were collected to assess their impact on the carbon stock variation. Secondary data relevant to this study were collected from different resources, including published and unpublished materials, books, journal articles, reports, and electronic websites.

#### Field measurements and biomass estimation for carbon pools

*Field measurement and biomass estimation of above-ground biomass components*. Initially, all contributors to above-ground biomass (AGB) were meticulously selected, and a comprehensive list of these species in each plot was compiled along with their vernacular names. These plant samples were collected, pressed, dried, and then identified at the National Herbarium of Addis Ababa University (AAU) by comparing with authenticated specimens available in the Herbarium and referring to the published Flora of Ethiopia and Eritrea. The AGB measurement in this study encompasses the measurement of various plant categories, including all forms of trees (trees, tree ferns, palms, bamboos), lianas, shrubs, saplings, and standing dead woods.

**Trees, tree ferns, palms, lianas**: The measurement of trees, tree ferns, and palms over a minimum diameter of 5 cm in the proper nested plots were measured for their DBH and height. For Bamboo DBH, each age class was measured in the proper nested plots. Age classes were determined following Wimbush [47] and Banik [48]. Lianas rate were measured for their DBH vertically (d_vert_) at 1.30 meters above the ground. Then, for each liana, we converted the vertical diameter (d_vert_) into the diameter at 1.3 meters along the stem (d_1.3_) using the Schnitzer *et al*. [49] equation as used by van der Heijden and Phillips [50]. For shrubs, we measured the maximum height of the shrubs (h) and the average crown radius (r) to estimate the volume of the shrubs, assuming a conical geometry [51].

**Sapling:** A destructive sampling technique was used in a single 3m x 3m plot located outside the permanent plots for each stratum. All the sapling individuals (trees less than 5 cm DBH) within this plot were counted (c), completely harvested, and weighed on the field for their total fresh weight using a hanging balance. Then, some portions of the sample (500 gm) were brought to the Ecophysiology laboratory (Addis Ababa University), where it was oven-dried. The total dry weight (Tw) was then extrapolated from this dried sample. Finally, the total dry weight of the saplings (Tw) was divided by their total number (c) to estimate the average dry weight of an individual sapling.

**Fig 5:**
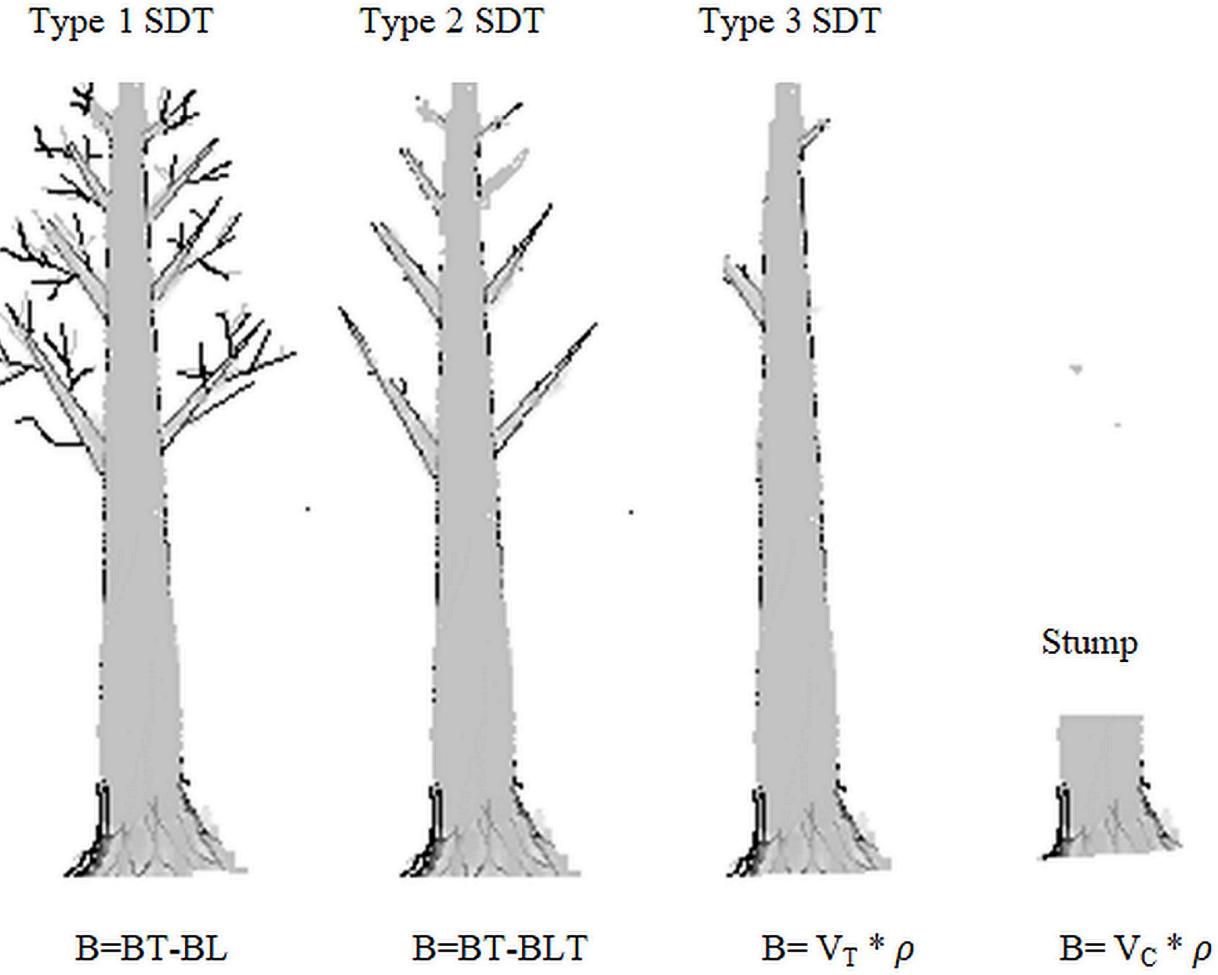
Typical sketches for the different types of standing dead trees (SDT) in Sele-Nono forest and estimation method of their above ground biomass (SDT refers to standing dead tree, B refers to AGB of dead trees, BT is AGB of live tree, BL is biomass of leaves (equivalent to 3% of BT), BLT is biomass of leaves and twigs (equivalent to 20% of BT), is wood density of ‘sound’ dead wood, VT is volume of truncated cone, Vc is volume of cylinder. Sketch is by the authors.

**Standing dead woods (SDT)** were measured for their DBH and height (H) simultaneously with live trees in the appropriate nests but marked as ‘Dead’ on the datasheet. In the current study area, standing dead trees were categorized into three types as used in [52] (Fig 5).

*Estimation of total above-ground biomass of a plot and its carbon stock*. Table 3 summarizes the basic field-measured parameters and the methods used for estimating the AGB of all components. The total above-ground biomass density was converted from kilograms per square meter (Kg/m^2^) to tons per hectare (t/ha) using a conversion rate of 10 [53]. Then, the total AGB for each sample plot was calculated by adding up the biomass of All AGB components (t/ha).


$$AGB_{total\;(\;per\;plot\;)}=AGB_{trees}+AGB_{palms}+AGB_{tree\;ferns}+AGB_{lianas}+AGB_{bamboos}+AGB_{shrubs}+AGB_{saplings}+AGB_{standing\;dead\;woods}$$ (1)

**Table 3:** Field measurements and biomass estimation of AGB component of the studied forest.

S/N	AGB component	Field measurement	Biomass estimation method Eq. No.	Reference	Remark
**Standing live woods**					
1	Tree (‘true’ trees)	DBH, H & *ρ*	AGB = 0.0673(*ρ*(DBH)^2^H)^0.976 …^……….….…. (2)	[54]	*ρ* is from [55–58]
2	Palm	Height (h)	AGB = 0.3999+7.907*H ……………………. . .. . ..(3)	[59]	
3	Tree fern	Height (h)	AGB=1423.4*exp(0.3233*H)…. . .…. (4)	[60]	
4	Liana ramet	Computing DBH at 1.3 m of the liana ramet from its vertical DBH as follow: $d_{1.3}=0.070+1.02*d_{vert}$	AGB = exp (-1.484+2.657ln (DBH)) ………. . .. . .(5)	[49]	Here we use not vert. DBH, but converted to DBH at 1.3 m (d_1.3_)
5	Bamboo	DBH	AGB(<1year) = exp (0.172*DBH) ……………. (6)	[47,48,61]	
			AGB(1-3year) = exp (0.289*DBH) …………. . . . (7)		
			AGB(>3year) = exp (0.30*DBH) ……….….…. (8)		
6	Shrubs	Volume from DBH and shrub height $V=\frac{1}{3}{\pi r}^{2}h$	$AGB=(\frac{1}{3}\pi r^{2}h)*\rho$…………………..… (9)	[41,46,62,63]	*ρ* is from published data
7	Saplings	Counting saplings	*AGB* = *Average dry weight of a sapling X* *total number of samplings*………….. (10)	[46]	
**Standing dead trees (SDT)**					
8	SDT Type 1	DBH, H & *ρ*	AGB = 97% of AGB of live trees……………. (11)	[64]	We utilized deduction method [64]
9	SDT Type 2	DBH, H & *ρ*	AGB = 80% of AGB of live trees………….…. (12)	[64]	
10	SDT Type 3	Volume of bole assuming it is a truncate cone using this formula where r_1_ computed from DBH and r_2_ computed from D_top_ $\left(V_{T})=\frac{1}{3}\pi h(r_{1}^{2}+r_{2}^{2}+r_{1}Xr_{2}\right)$ $D_{top}=D_{base}-[H*\frac{D_{base}-DBH}{130/100}]$	$AGB=\frac{1}{3}\pi h(r_{1}^{2}+r_{2}^{2}+r_{1}Xr_{2})*\rho$…….. (13)	[64]	
11	Stumps	Volume of stump assuming it look cylinder (*V*_*c*_) = *πr*^2^*h*	$AGB=(\pi r^{2}h)*\rho$………………… ……… (14)	[64]	*ρ* is ‘sound’ density of LDWs

*Estimation of belowground biomass and carbon stock*. Below-ground biomass (BGB) is often estimated from root-shoot ratios (R/S) by taking 20% of aboveground biomass [10,45,65]. Thus, the total BGB on each plot (t/ha) was computed by summing all BGB for all AGB components. Then, this was converted to root carbon stock using a conversion factor of 0.47 [10].


BGB=0.20*AGB (15)

*Herbs and litter*. All herbaceous vegetation within the designated plot areas for herbaceous biomass data collection (1m^2^) was cut at ground level. Likewise, all litter, including forest floor material and lying dead wood with diameters less than 10 cm, within the same plots was collected and weighed after completely clearing the herbs. Subsequently, approximately 200–500 grams of evenly mixed composite samples of herbs and litters were sub-sampled separately into plastic bags and transported to the Ecophysiology laboratory at Addis Ababa University. In the laboratory, these samples were oven-dried at 70°C until a constant weight was achieved [66] to determine their moisture content, from which their total dry mass could be deduced. The amount of biomass in herbs (HB) and/or litters (LB) per unit area was estimated as follows as used by Pearson *et al*. [65].


$$HB=\frac{Sub\;sample\;dry\;weight\;(gm)}{Sub\;sample\;fresh\;weight\;(gm)}X\frac{Total\;fresh\;weight\;of\;the\;sub\;plot\;(Kg)}{Sample\;area\;of\;the\;subplots\;(m^{2})}$$ (16)

Thus, the dry mass was extrapolated to biomass density (t/ha) using a conversion factor of 10 [53]. The carbon stock in herbaceous and litter biomass was calculated by multiplying their biomass by 0.47 [64].

*Lying dead woods*. The lying dead woods (LDWs) of the study forest were categorized into three decay classes: sound (recently fallen), intermediate (partly rotten), and rotten (very decayed but not completely decomposed) using a machete test following Walker *et al*. [46]. If the blade rebounded, producing sound, it was classified as ’sound.’ If the blade entered slightly, it was categorized as ‘intermediate’. If the strike caused the wood to break apart into pieces, it was assigned as ’rotten’ [10,45,67]. To estimate the biomass of lying dead woods (LDWs), two crucial sets of data were gathered on the field (diameters and wood densities of each class of LDWs) using line-transect methods as recommended by Warren and Olsen [68].

The line transect method utilized in this research entailed placing two 50 m transect lines on a flat terrain basis (total length L = 100 m), positioned at right angles outside the main plot. The actual length of the transect line in the field varied based on the slope of the terrain (S1 Table). The direction of the first line was randomly established after walking 100 paces from the center of the main plot, and the second line was positioned at a right angle to the starting point of the first line (S6 Appendix). Then, the diameter of each piece of LDWs (d_1_, d_2_, d_3_…….d_n_) that were intersected along the length of the line was measured. The measured diameters were then used to calculate the volume (m^3^/ha) of each class of LDWs as follows [68–70].

$${Volume\;of\;LDWs=\pi }^{2}\times [\frac{{d1}^{2}+{d2}^{2}+{d3}^{2}\ldots \ldots ..{dn}^{2}}{8L}]$$ (17)

Wood density for each LDWs class was determined through the Machete test in the field (for identifying LDWs classes) and Archimedes principle in the lab (for measuring LDWs volume). To achieve this, 90 samples of dead wood discs were collected in each density class using a hand saw. These samples were taken to the Addis Ababa University Ecophysiology laboratory to measure their dry mass using oven drying and their volume from the floatation method following [69].

$$Density\;of\;wood\;discs\;of\;LDWs=\frac{Dry\;mass\;of\;the\;disc}{Volume\;of\;the\;disc}$$ (18)

Finally, the wood densities of each LDWs class were averaged to compute their respective mean wood densities. The biomass in lying dead woods (LDWB) per plot was computed by multiplying the volume of each class with its respective wood density. To determine the carbon stock within lying dead woods, overall biomass was multiplied by a conversion factor of 0.47 [64].


LDWB=VolumeofLDWsXWooddensity (19)

#### Estimation of soil carbon stock

This study collected two types of soil samples: composite soil samples (for assessing soil organic carbon concentration) and core soil samples (for determining bulk density). Both types of soil samples were obtained from nested plots specifically designed for this purpose at a depth of 30 cm. Composite soil samples were derived from subsamples taken from all five nested plots, thoroughly mixed, and about 150 grams of the composite samples were collected in cloth bags to represent each main plot. From these samples, the percentage of organic carbon were analysed.

Additionally, undisturbed core soil samples (with a core volume of 98 cm^3^) were collected from the center of the plot following the method outlined by Berenguer et al. [71] for soil bulk density analysis. This involved drying the core samples at 105°C for 12 hours and dividing the resulting dry weight by the volume of the core sampler, which is 98 cm^3^.

Then, the amount of carbon stock (SOC) per unit area of soil was determined following the method of IPCC [10] as follows:

SOC(tC/ha)=%C*ρ*D*100 (20)

Where %C is soil organic carbon concentration as determined from the laboratory analysis, and expressed as a decimal fraction, *ρ* is soil bulk density (g/cm^3^) calculated from undisturbed soil using core sampler, D represents the depth of the sample soil layer (in cm), which was set at 30 cm since a significant portion of soil carbon is expected to be stored within the top 30 cm [72]; and 100 is a conversion factor unit from g/cm^2^ to t/ha.

#### Estimation of the total carbon stock and amount of atmospheric carbon dioxide equivalence removed by Sele-Nono forest

The total carbon stock density of the forest was calculated by summing all the carbon stock densities of each carbon pool using the following formula, provided that all carbon values were reported on a horizontal projection.

I. The carbon stock density of a plot was calculated as follows:

$$C_{plot}=C_{AGB}+C_{BGB}+C_{LB}+C_{HB}+C_{LDWB}+C_{soil}$$ (21)

Where, C plot = Carbon Stock Density [t ha^-1^]

CAGB = Carbon Stock in Above Ground Biomass [t ha^-1^]

CBGB = Carbon Stock in Below Ground Biomass [t ha^-1^]

C LB = Carbon Stock in Litter Biomass [tha^-1^]

CLDWB = Carbon Stock in lying dead woods Biomass [t ha^-1^]

C HB = Carbon Stock in Herbs Biomass [t ha^-1^]

C _soil_ = Carbon stock in soil (Soil Organic Carbon) [t ha^-1^]

II. Then, the carbon stock (tons C/ha) for each stratum was computed by multiplying the average carbon stock of a plot by its area (ha).

$$C_{stratum}=\left(\frac{\sum C_{plot}}{n_{plot}}\right)X\;Area\;of\;stratum$$ (22)

Where *C*_*stratum*_ refers to the carbon stock of a stratum, ∑*C*_*plot*_ refers to the summation of the total carbon stock of each plots within the stratum, *n*_*plot*_ refers to the number of plots within the stratum.

III. To obtain the total carbon stock of the forest, we summed up all the carbon densities in each stratum.

$$C_{Whole\;forest}=\sum C_{stratum}$$ (23)

Where *C*_*Whole forest*_ refers to the carbon stock of the studied forest, ∑*C*_*stratum*_ refers to the summation of the carbon stock of each strata of the studied forest.

IV. Ultimately, to estimate the amount of carbon dioxide equivalence (CO_2_eq.) that has been effectively removed from the atmosphere and stored in the forest, we multiplied the total carbon stock by the conversion factor of 3. 67 assuming that each ton of stored carbon is equivalent to 3.67 ton of CO_2_ removed from the atmosphere [46].


$${CO}_{2}eq.=C_{Whole\;forest}\times 3.67$$ (24)

### Statistical analysis

The gathered data on estimated biomass and/or carbon stocks underwent analysis via both descriptive and inferential statistical methods using R-software version 4.3.2. Descriptive statistics, such as frequency, percentage means, standard deviation, or standard errors, were used to summarize the biomass and/or carbon stocks in each pool of the study area. Analysis of Variance (ANOVA) and Post hoc analysis using Tukey’s pairwise tests were conducted to assess the significant differences in carbon stocks among carbon pools and across environmental gradients (Altitude, slope, and disturbance levels). Altitude categories and disturbance levels were considered in this study in the same way as used by the previous study in the Sele-Nono forest [42]. Similarly, slope categorization was considered in this study, as used by Berhanu et al. [73]. A correlation test was used to test the relationships between carbon stocks in above-ground biomasses and their dendrometric variables, such as height and DBH.

## Results

### Above-ground biomass and carbon stock of Sele-Nono forest

#### Contribution of components to the total above-ground biomass

In this research, saplings were treated collectively as one species, while the four distinct types of standing dead woods were individually regarded as separate species. This approach aimed to simplify the estimation of carbon stocks. Consequently, the study identified 206 species, organized into eight components; trees, palms, tree ferns, lianas, bamboos, shrubs, saplings, and standing dead trees (SDTs) (S2 Table). Results of this study showed that trees mainly accounted for the total mean above-ground biomass (TAGB) of the forest, whereas tree ferns contributed the least (Table 4).

**Table 4:** Contribution of above-ground biomass components to the total above-ground biomass of Sele-Nono forest.

S/N	AGB component	Species No.	Mean AGB (t/ha) per component	Contribution (%) of AGB components to the TAGB (t/ha)
1	Trees (‘true’ trees) (T)	111	178.22	94.2
2	Palms	1	4.35	2.30
3	Tree ferns	1	0.26	0.14
4	Lianas (L)	25	1.32	0.70
5	Bamboos (B)	1	0.41	0.22
6	Shrubs (S)	62	2.89	1.53
7	Saplings	1	0.66	0.35
8	SDTs	4	1.05	0.56

#### Above-ground biomass of plant species

Among the 206 species documented in this study, the majority of carbon was found to be stored in species like *Schefflera abyssinica*, *Ficus vasta*, *Schefflera volkensii*, *Ficus ovata*, *Manilkara butugi*, *Olea welwitschii*, *Pouteria adolfi-friederici*, *Trilepisium madagascariense*, *Morus mesozygia*, and similar species. Conversely, shrubs such as *Dombeya torrida*, *Senna petersiana*, *Pentas lanceolata*, *Triumfetta brachyceras*, *Ricinus communis*, *Solanum anguivi*, and related species exhibited the least recorded carbon stock (S2 Table).

#### Carbon sequestration of typical woody plants

This research investigated how much a typical woody species could store carbon. The findings revealed that a typical tree species in the Sele-Nono forest stores an average of 0.99±0.16 tons of carbon per plant in its AGB. In contrast, the average AGB per sapling was almost negligible. Additionally, a typical woody plant within the AGB category can sequester approximately 1.28±0.29 tons of carbon dioxide equivalent. The mean values (mean ± standard error) for AGB, AGC, and the sequestered amount of CO_2_ eq per a single woody plant are detailed below (Table 5).

**Table 5:** Role of individual woody plants in mitigating atmospheric CO_2_ in Sele-Nono forest.

Components of AGB	Abundance (Total number of individuals)	Species richness (N)	Av. AGB (ton) per individual plant	Av. AGC (ton) per individual plant	Av. CO2eq. (ton) removed by individual plants
Trees	4789	111	0.99±0.16	0.47±0.07	1.71±0.27
Palms	81	1	0.19	0.09	0.32
Tree ferns	72	1	0.01	0.01	0.01
Lianas	509	25	0.03±0.01	0.01±0.01	0.05±0.01
Bamboos	109	1	0.01	0.01	0.02
Shrubs	761	62	0.011±0.01	0.011±0.00	0.01±0.00
Saplings	5958	1	≈0.00	≈0.00	5.0022E-06
SDT	240	4	0.03±0.01	0.01±0.01	0.05±0.01
**total**	12410	206			
**Average**	1773	206	0.74±0.17	0.35±0.08	1.28± 0.29

**Fig 6:**
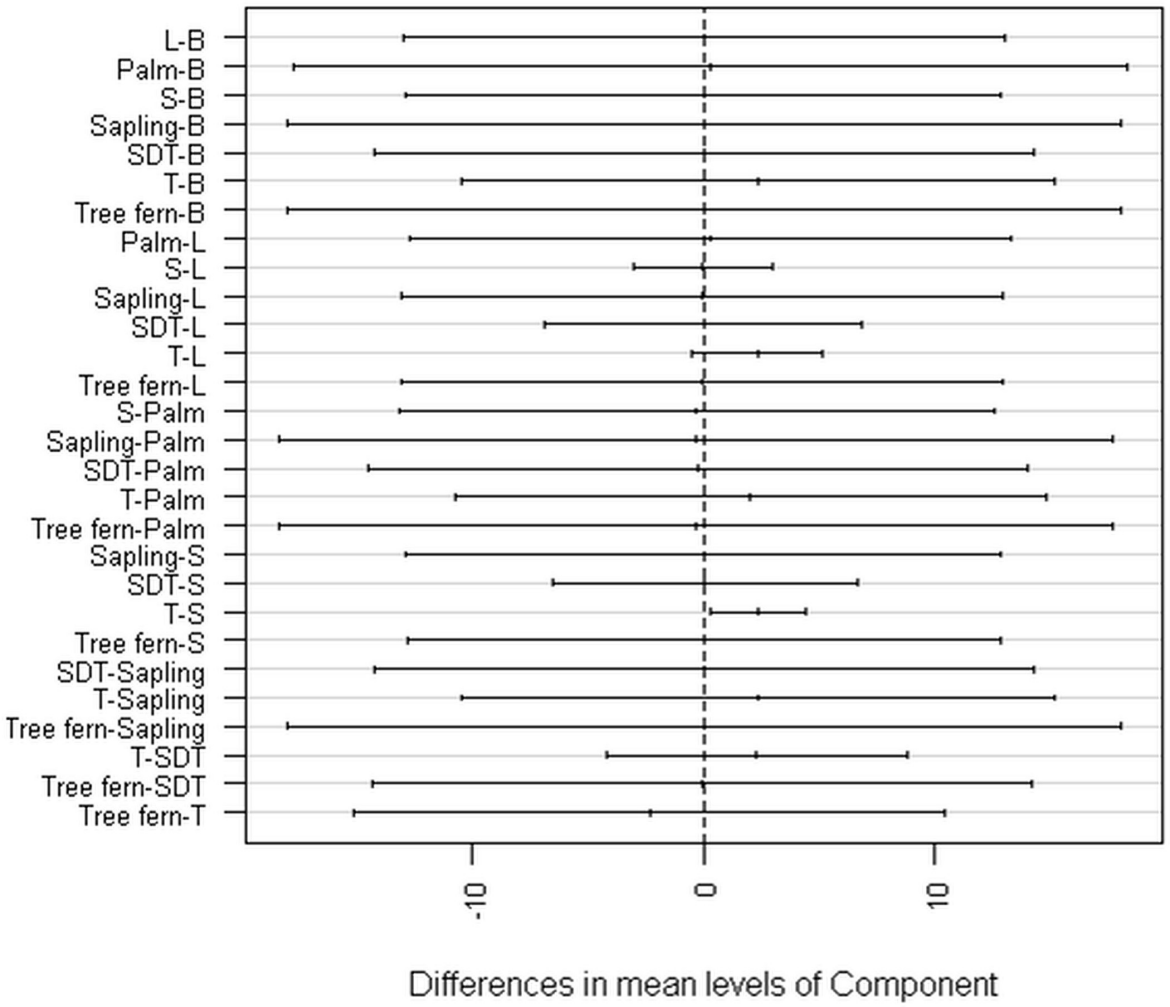
Tukey test–95% family-wise confidence level. The graph shows the value of the difference between the means and their respective 95% CI for each combination of groups. The vertical dashed line indicates the point where the difference between the means is equal to zero, that is, the means are equal. It is important to note that those intervals crossing zero indicate that the mean differences between the compared groups are insignificant. (Note that L = liana, B = bamboo, T = true tree, SDT = standing dead tree, S = shrub).

Analysis of variance indicates that there are differences in terms of carbon stock among the AGB components (F = 2.31, P = 0.03). However, post hoc analysis using Tukey’s HSD test confirmed that the prominent difference is between trees and shrubs, and there is no significant variation in the remaining AGB components regarding their carbon stock or the carbon mitigation role of the individual woody plant is concerned (Fig 6).

This finding implies that the carbon offsetting capacity of a woody plant does not mainly vary because of its classification as a tree, palm, bamboo, or liana. However, a three-way ANOVA analysis confirmed that the carbon content within the AGB of woody plants significantly fluctuates with height (H) and DBH (P<0.01), but not due to their wood density (***ρ***) (P>0.05) (Table 6).

**Table 6:** Three-way ANOVA for three independent variables on one dependent (AGC) variable.

Response: AGC					
	**Df**	**Sum Sq**	**Mean Sq**	**F value**	**Pr(>F)**
DBH	1	184.8	184.8	691.5	<2.2e-16 ***
H	1	41.1	41.1	153.7	<2.2e-16 ***
WD	1	0.1	0.1	0.5	0.5
Residuals	174	46.5	0.3		

Note: Signif. codes: 0 ‘***’ 0.001 ‘**’ 0.01 ‘*’ 0.05 ‘.’ 0.1 ‘ ‘ 1.

**Fig 7:**
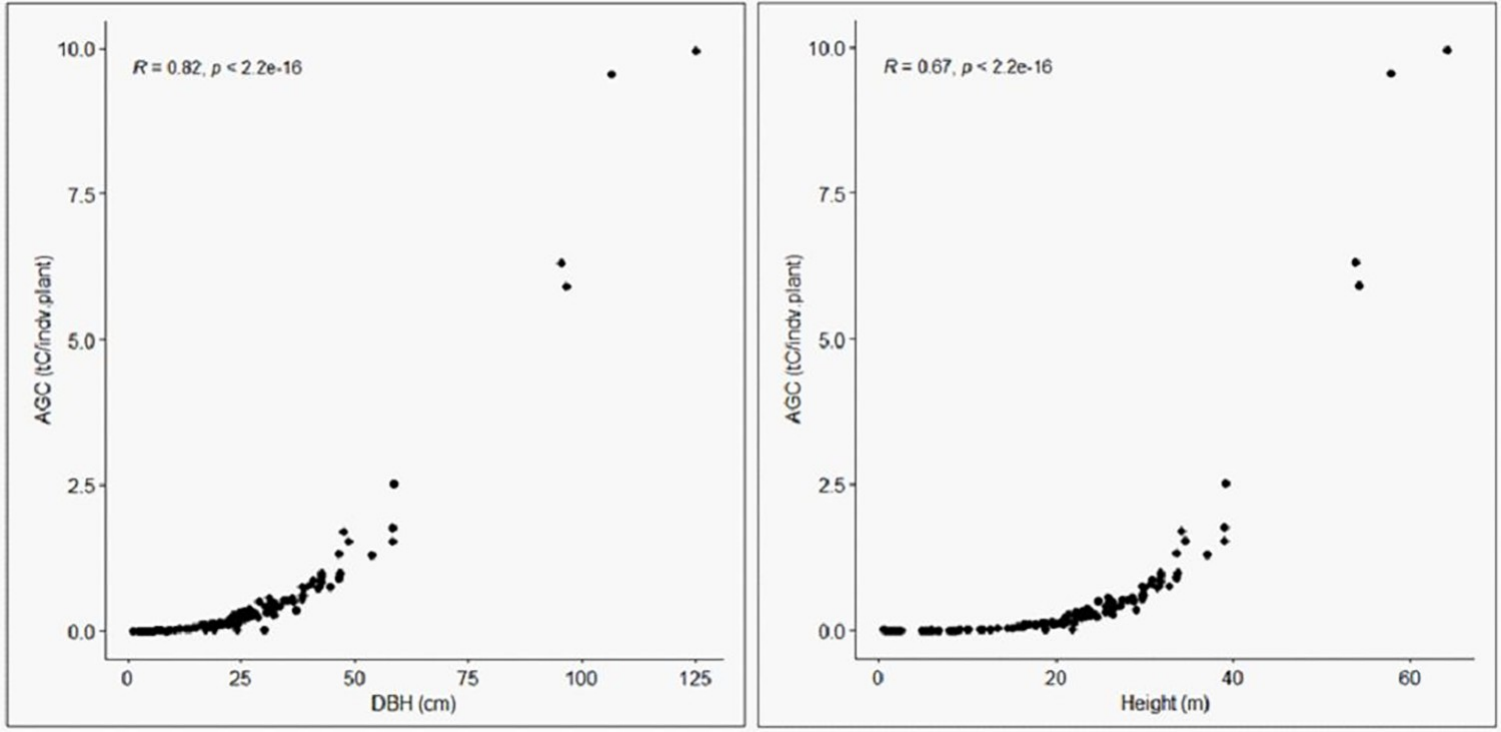
Correlation plots showing the relationship between AGC with DBH and height.

Although DBH and height of the woody component of Sele-Nono forest contribute significantly to the carbon stock, their DBHt was observed to be very detrimental (R = 0.82) as shown by the correlation plot), even more than their height (R = 0.67) (Fig 7).

#### Wood density of lying dead woods

**Fig 8:**
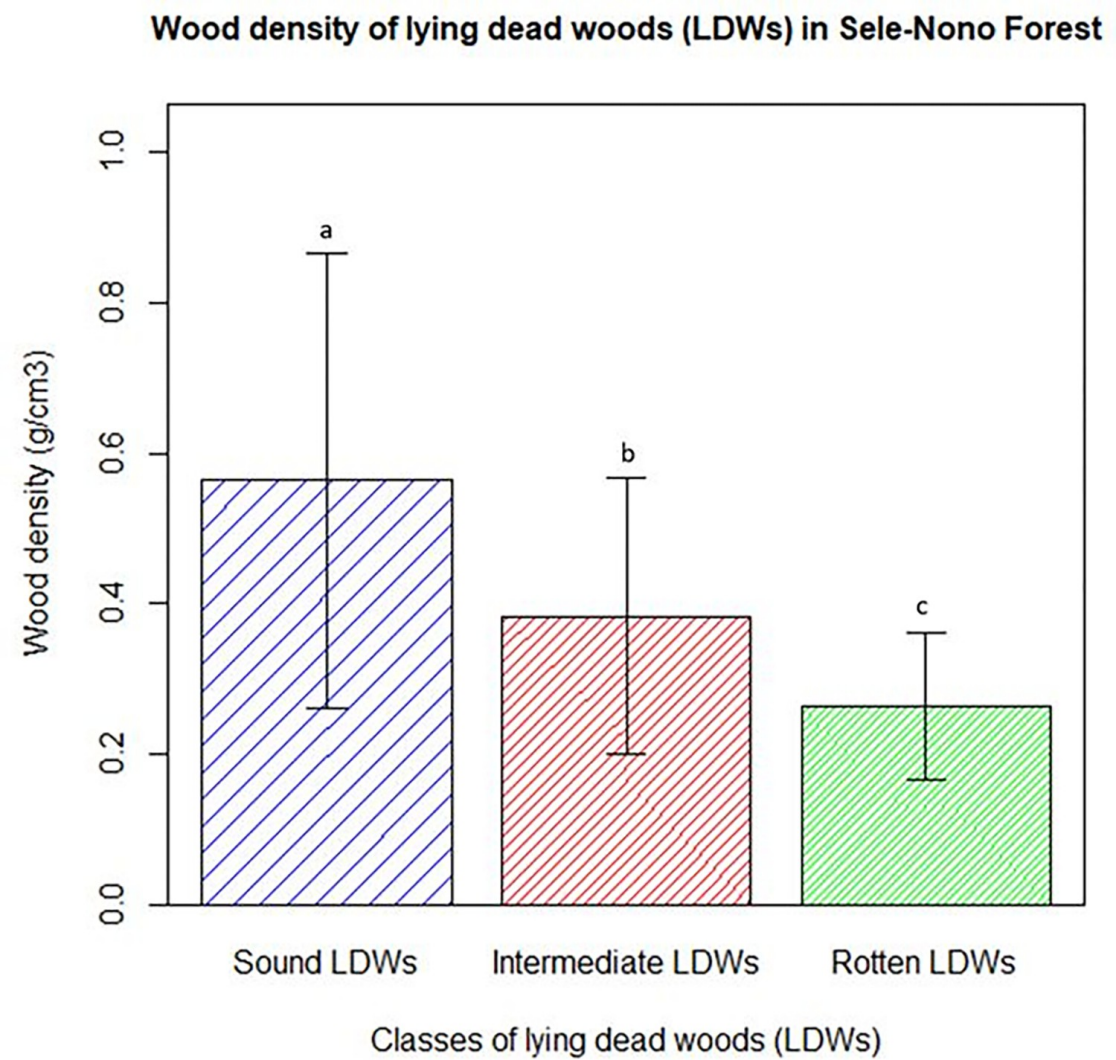
Wood density report for LDWs in Sele-Nono forest.

This study produced wood density measurements for the three categories of lying dead woods (LDWs). The densities identified for sound LDWs (0.56 g/cm^3^), intermediate LDWs (0.38 g/cm^3^), and rotten LDWs (0.26 g/cm^3^) were used to estimate the biomass of LDWs in the studied forest (Detailed result is in S3 Table). The distribution of the wood density data for each class of LDWs is visually displayed as follows (Fig 8).

#### Biomass of the studied forest

**Fig 9:**
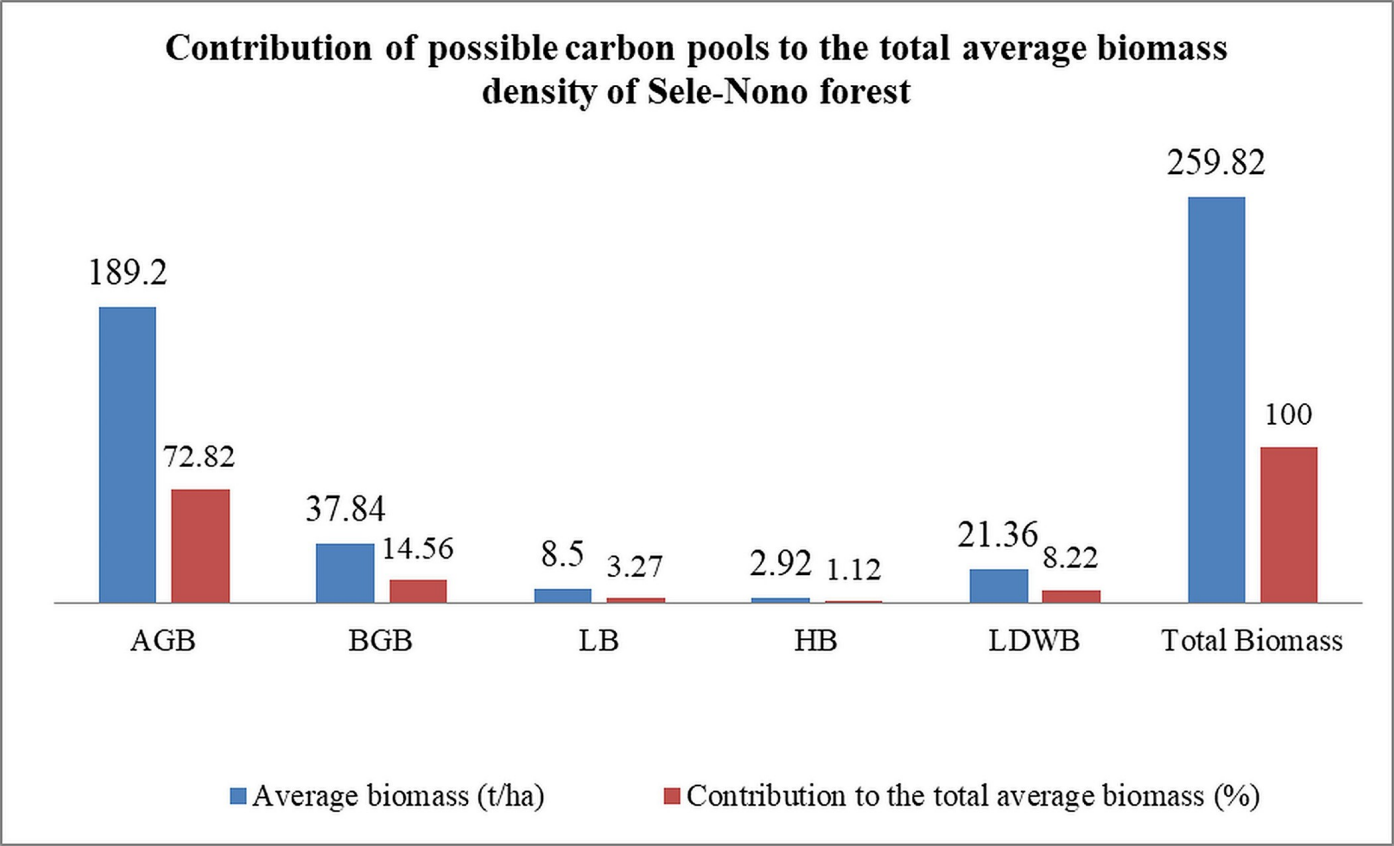
Contribution of possible carbon pools to the total average biomass density of the study forest.

The study’s results show that the mean total biomass of the forest is estimated at 259.82±122.59 t ha-1 (S4 Table). This total includes the mean (mean± SD) values of above-ground biomass (AGB), below-ground biomass (BGB), litter biomass (LB), herb biomass (HB), and lying dead wood biomass (LDWB) (S4 Table). The largest portion of this biomass comes from AGB (72.82%), followed by BGB (14.56%), lying dead wood (8.22%), and litter (3.27%). Herbaceous vegetation contributes minimally to the forest’s total biomass (1.12%) (Fig 9).

*Carbon stock in soil*. The outcome of this study also revealed that Sele-Nono forest harbors carbon in its soil with a range of 36.38tha^-1^ to 356.49tha^-1^, and the mean amount of carbon stored in the soil (SOC) is 162.69 ± 78.73 tha^-1^ (S5 Table).

*Carbon stock density of the studied forest*. The total carbon stock density of the study forest was computed by summing all the carbon stock densities of each pool for all plot samples of the study area. Accordingly, the total carbon stock density of the study forest ranged from 90.99 to 484.88 t ha^-1^ (S4 Table), with a mean carbon stock density of 284.81 ± 107.81 t ha^-1^. The detailed carbon stock of each carbon pool is shown below (Table 7).

**Table 7:** Mean carbon stock of each carbon pool in Sele-Nono forest.

Carbon pools in Sele-Nono forest	Abbreviations given for carbon stock in each C-pools	Mean carbon stock density (t ha^-1^)
Above-ground biomass (AGB)	AGC	88.92±43.06
Below ground biomass (BGB)	BGC	17.78±8.61
Litters	LC	4.01±1.76
Herbs	HC	1.37±0.48
Lying dead wood (LDWs)	LDWC	10.04±6.35
Soil	SOC	162.69±78.73
**Total Mean carbon stock**		**284.81±107.81**

**Fig 10:**
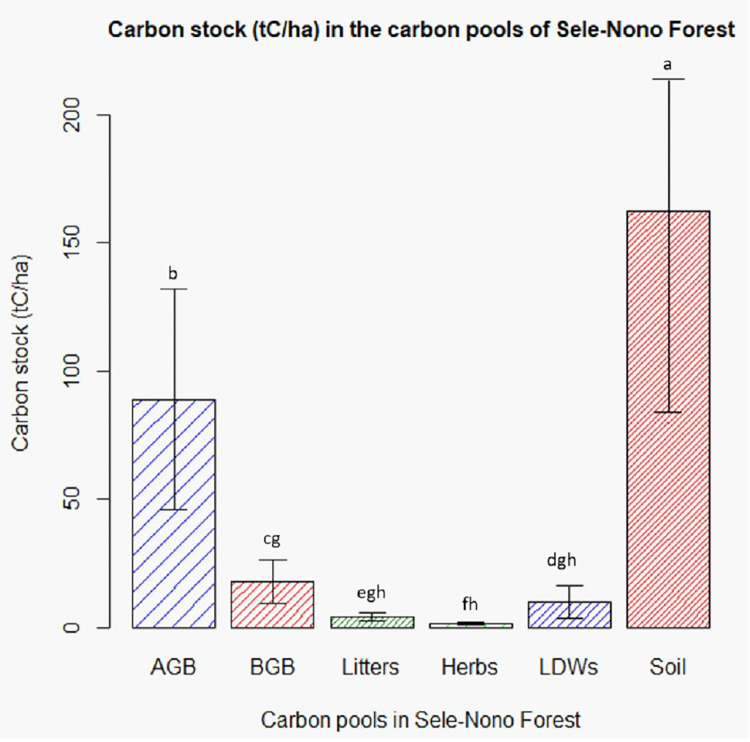
Carbon stock in the various carbon pools of Sele-Nono forest (error bars of the bar plots with the same letter are not significantly different (P > 0.05).

Results from ANOVA examination reveal a notable difference in carbon stock among different carbon pools of the studied forest (F = 281.44, p<0.05). Post hoc analysis through Tukey’s pairwise test revealed a significantly higher carbon stock in soils and AGB, which differ significantly from each other and with the remaining carbon pools. The study also indicated that the carbon stored in BGB, litters, herbs, and lying dead woods (LDWs) did not exhibit significant differences among themselves (Fig 10).

**Fig 11:**
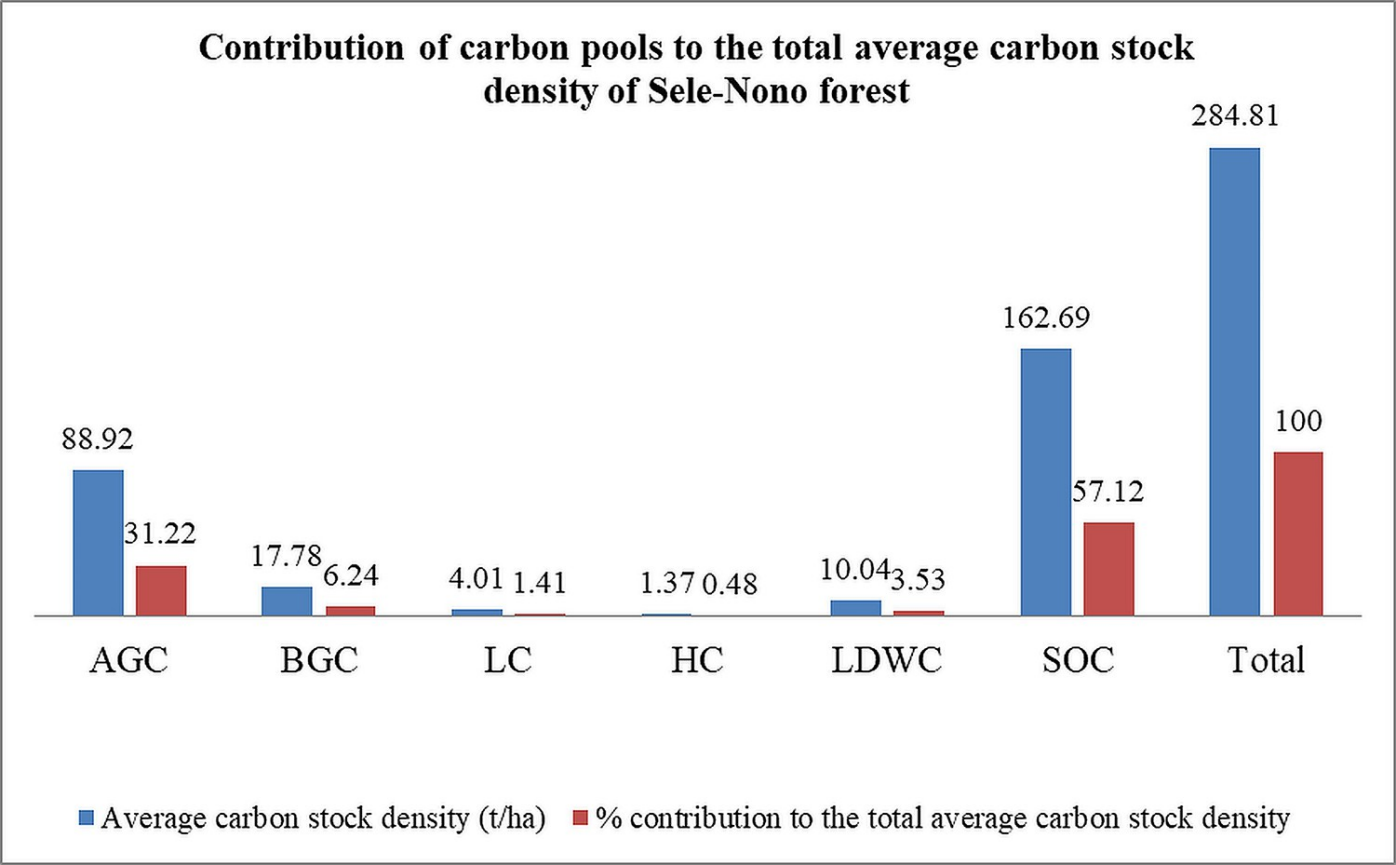
Contribution of carbon pools to the total carbon stock density in Sele-Nono forest.

The study also reveals that most carbon stored in the forest is primarily from soil carbon (57.12%) and above-ground biomass (31.22%). Fig 11 illustrates the percentage distribution of average carbon stock density (t/ha) among AGB, BGB, LB, HB, LDWB, and soil organic matter.

*Carbon stock and economic value of the studied forest for climate change mitigation*. Overall results show that the studied forest has stored 42.81 Mt of carbon. This was obtained by summing all the carbon stocks across each stratum (Table 8), which was computed following the state of the art (Eq 22).

**Table 8:** Total current carbon stock of Sele-Nono forest in each stratum (S refers to strata number, ∑ denotes summation).

Strata code	Number of plots (*n*_*plot*_)	Sum of carbon stock (∑*C*_*plot*_) (t/ha)	Area of stratum (ha)	Carbon stock of each stratum (*C*_*stratum*_) ton Mega ton (Mt)	
**S-1**	11	1801.8	18373.1	3009492.0	3.01
**S-2**	22	6969.5	36746.2	11641014.0	11.64
**S-3**	22	6761.1	36746.2	11292998.6	11.29
**S-4**	20	7552.7	33405.6	12615087.7	12.62
**S-5**	14	2420.7	23383.9	4043323.1	4.03
**S-6**	1	127.2	1670.3	212443.0	0.21
**Total**	90	25633.0	150325.3	42814366.7	42.81

The estimated atmospheric CO_2_eq reduced due to the present stand of the forest was calculated following the state of the art as CO_2_eq. = *carbon stock of the forest*×3.67. Consequently, it was possible to deduce that about 157, 128, 725.68 tons of atmospheric CO_2_eq have been sequestered and mitigated by Sele-Nono forest. If this amount of air is marketed to individuals, communities, companies and/ or governments at an average price of 11 dollars per tCO_2_ eq. [74], the anticipated financial gain for local communities as a benefit from CO_2_ mitigation would exceed one billion dollars. This economic study of the forest is computed as follows:

$$Economic\;benefit\;of\;the\;study\;forest=\frac{157,128,725.68\;ton\;of\;{CO}_{2}eq.X11\;\$}{1ton\;of\;{CO}_{2}eq.}=1.73billion\;\$$$

**Fig 12:**
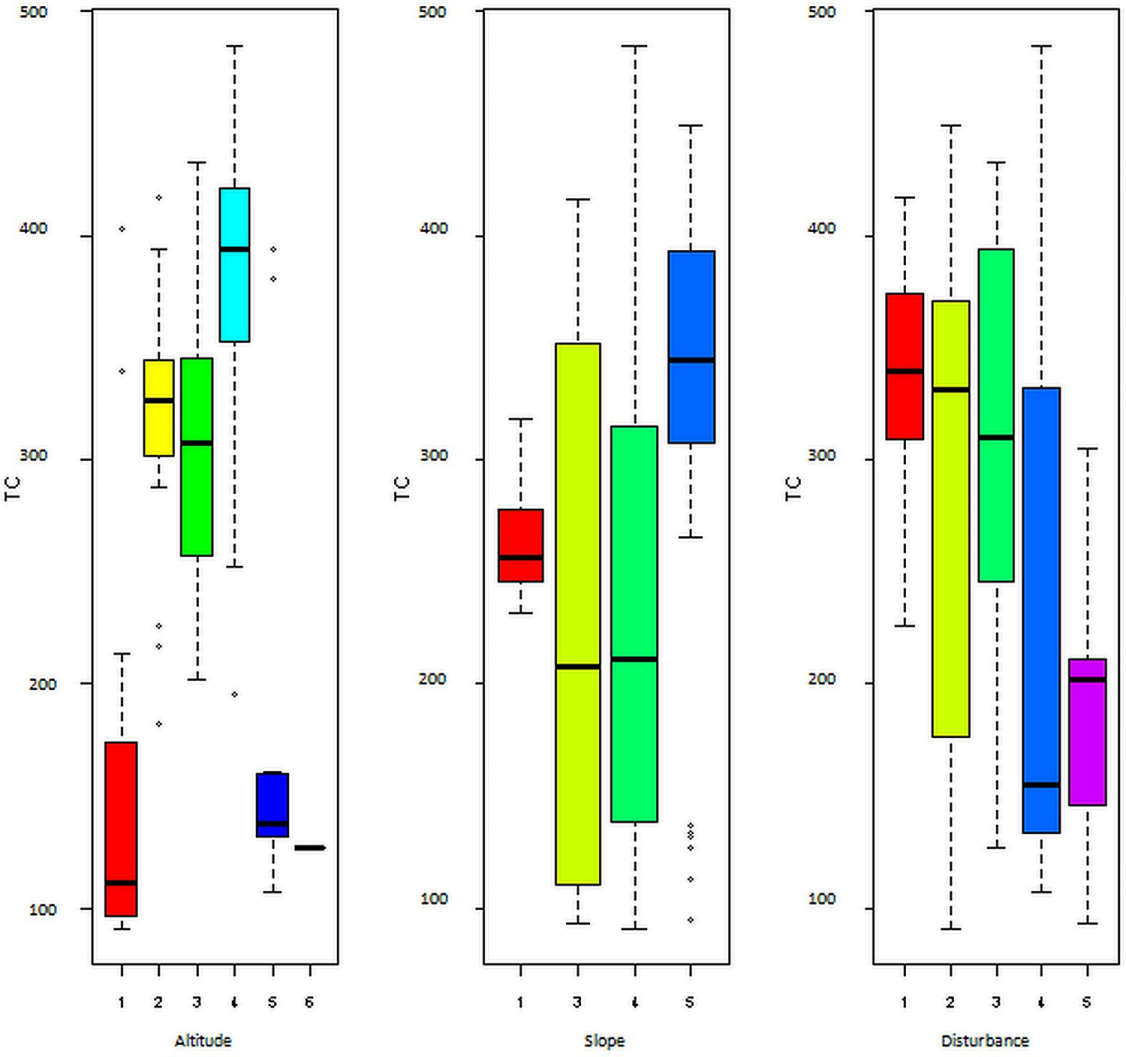
Spatial variation of total carbon stock (TC, tC/ha) in Sele-Nono forest across three environmental factors (Altitude, 1: 2182–2448, 2: 1994–2181, 3: 1646–1993, 4: 1378–1645, 5: 1109–1377, 6: 840–1108; Slope, 1: 0–3%, 2: 3.1–8%, 3: 8.1–15%, 4: 15.1–30%, 5: 30.1–50%; Disturbance: 0–20% of the sample plot disturbed, 2: 21–40% plot disturbed, 3: when 41–60% of the plot disturbed, 4: 61–80% of the plot disturbed, 5: 81–100% of the plot disturbed).

*Carbon stock variation along environmental gradient*. Results of this study revealed that the three environmental factors that were detected to be significant to influence the structure of plant communities of Sele-Nono forest [42] were also found to produce significant variation (P<0.05) in carbon storage (Fig 12).

**Fig 13:**
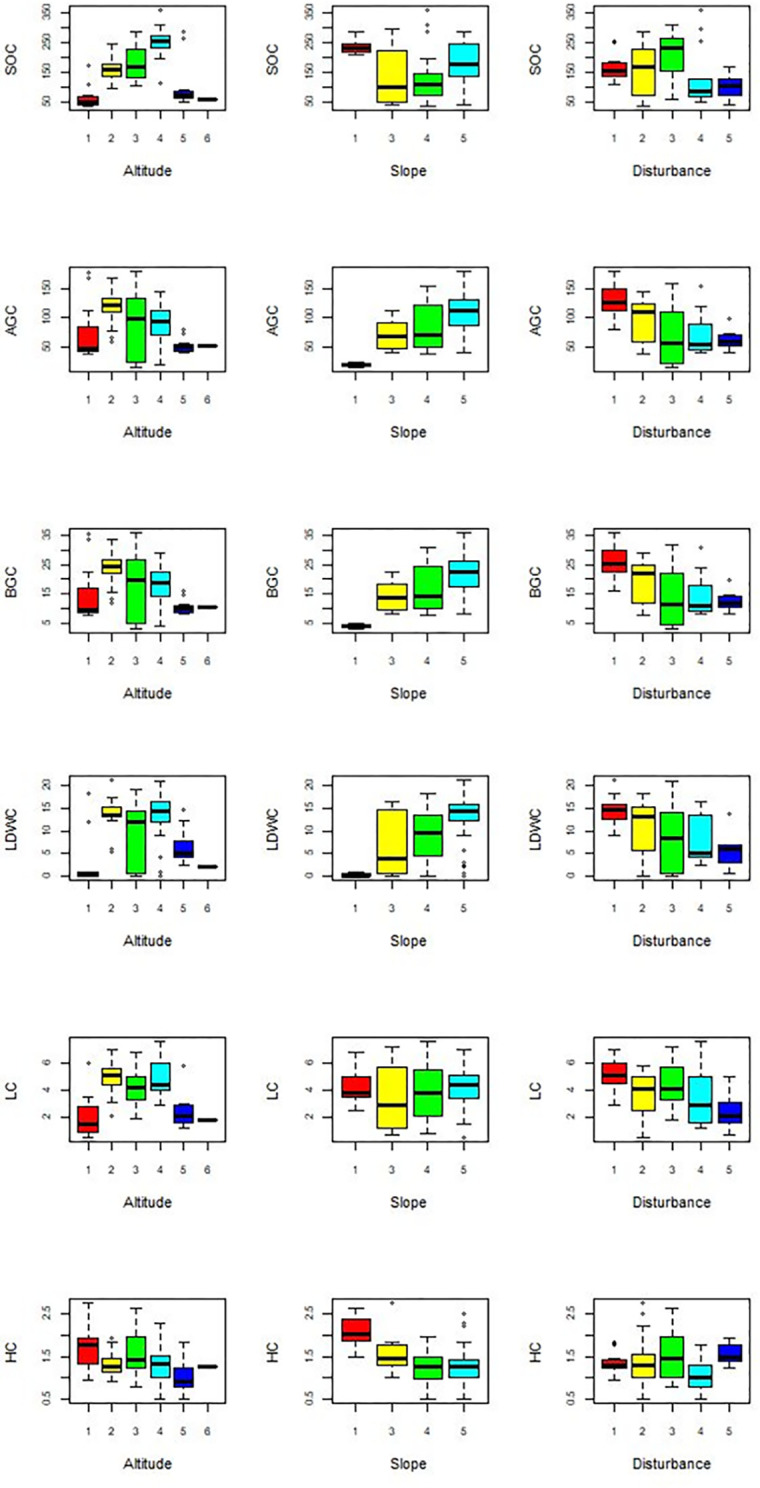
The impact of various environmental factors on the carbon stock of different carbon pools in the Sele-Nono. Figure (a) displays the variations in SOC, AGC, and BGC across different altitudes, slopes, and disturbance levels. Figure (b) shows the variations in LDWC, LC, and HC across the same environmental gradients.

Moreover, the collected data shows the different carbon pools have different roles in the total carbon sum when these environmental factor vary. The detail result of the carbon stock of each of the studied the carbon pools is displayed below (Fig 13). From the results (Figs 12 and 13), it can be revealed that carbon stock in Sele-Nono forest varies not only within its carbon pools but also across environmental gradients.

## Discussion

### Carbon stock disparities among above-ground biomass components

This study reveals that all the seven components of the AGB analyzed for Sele-Nono forest do not equally contribute to the total carbon stock of the forests’ ABG. Similar findings were noted in other studies conducted in different regions of Ethiopia [24–27,75–78]. In Sele-Nono Forest, the highest percentage of AGB and/or carbon stock comes from trees (also denoted in this study as ‘true’ trees), particularly species like *Schefflera abyssinica*, *Ficus vasta*, *Schefflera volkensii*, *Ficus ovata*, *Manilkara butugi*, *Olea welwitschii*, *Pouteria adolfi-friederici*, *Trilepisium madagascariense*, *Morus mesozygia*, and others. The high carbon stock of those tree species in the study area may be linked with their high abundances, roughly higher DBH and height, which were highly correlated with the AGB and carbon stock of plants [76,79]. The high contribution of trees to carbon stock potential and/or mitigation of climate change has also been evident in other studies [77], which highlighted the importance of a specific tree species in regulating carbon emissions from fossils. Thus, it could be deduced that trees act as a significant carbon pool of AGB components, making them essential in regulating carbon emissions and mitigating climate change.

Shrubby species have been noticed to have lower carbon storage compared to trees in the study area. This could be related to the short height and average canopy diameter of the shrubby components of the study forest. A similar pattern was observed in other rainforests of Ethiopia [17,27] and elsewhere in the wider tropical rainforests [8,60].

Similarly, the low mean carbon stock of lianas compared to trees in the same study area might be attributed to their lower DBH classes and less abundance of lianas with a DBH greater than 10 cm. Although saplings were the most abundant in Sele-Nono Forest, they made the least contribution to the total carbon stock of the forest. This may be attributed to their small size and age. Similar findings were noted by Neupane and Sharma [80], who found saplings contributed the least to the total carbon stock in the Nepal Forest.

Tree ferns, bamboo, and palm trees contributed relatively little to the total carbon stock of the above-ground biomass (AGC) of Sele-Nono Forest, which is about 0.14% to 2.3%. This could be due to their smaller abundance than other AGB components in the studied forest. Similar observations were noted in other montane rain forests [81]. For instance, these AGB components are estimated to account for less than 5% of the entire AGC of Atlantic Forests [82] and other neotropical forests [83,84]. Although these components contribute poorly to the entire AGC, Palms shows a relatively better contribution. Similar observations were noted elsewhere. For instance, the contribution of palms to the total AGC was found to be close to 1% in the Central Amazonia Forest [85], whereas it reached close to 5% in the Colombian tropical moist montane forest [86] and 5–7% in Costa Rican tropical wet forests [87]. Biological limits to their abundances and their height & DBH for those AGB components (palms, tree ferns & bamboo) might have made them contribute little to the total AGC of the forest.

This study also indicated that a typical single woody plant that belongs to the AGB component in Sele-Nono forest has a role in mitigating an average of 1.28 tons of CO2eq from the atmosphere. The highest removal was achieved by trees (1.71tCO2eq.), and the lowest was by saplings (5gm of CO2eq). Wardle et al. [88] also showed the substantial effect of a single woody plant on removing carbon dioxide from the atmosphere.

### Current carbon stock density in Sele-Nono forest

This study revealed that the mean (mean±SD) AGC of Sele-Nono forest was 88.92 ± 43.06tC/ha. This is the cumulative result of the carbon stocks in all components of AGB (trees, palm trees, bamboo, tree ferns, standing dead trees, shrubs, lianas, and saplings). This report is closer to the values of other moist southwest forests, such as Masha, Anderacha, and Gesha forests, which were estimated to have above-ground carbon at 76.83tC/ha [89]. Including saplings, lianas, and standing dead trees as part of AGC might have made the mean carbon stock density of Sele-Nono forest a bit higher than the value reported for Masha, Aderacha and Gesha forest. The carbon stock density of Sele-Nono Forest is found to be higher than the carbon stock density of the Ethiopian high forest, which was estimated at 101 tC/ha [35,36]. Similarly, the carbon stock density of Sele-Nono forest is very high when compared with the gross carbon stock report of the Ethiopian vegetation, which is 37 tC/ha as reported by Brown [35] and/or 47 tC/ha as reported by FAO [90]. This is likely because the report by Brown [35] and FAO [90] is a country-wide report and includes bushlands and scrublands that significantly reduce the reported mean carbon stock of the country when compared with the carbon stock report for forests.

However, our finding on the carbon stock of Sele-Nono forest is consistent with the Africa-wide estimates of 30–200 tC/ha [91] and global forest ranges of 20–400 tC/ha [92]. Our finding is also more comparable with the IPCC [8] estimation of AGC of tropical mountainous system of Africa and the tropical rain forest of Africa, estimated at 54.1 tC/ha and 145.7 tC/ha, respectively. The variation between our report for Sele-Nono forest and the estimate made for the other compared studies could be attributed to geographical variation. It may also be attributed to the variation in the allometric equations that were used for the carbon stock estimates of the compared forests. Moreover, variations in the methods for calculation and sampling techniques could also be another reason for the varying results.

The mean carbon stock in the litter pool (LC) of the present study was estimated at 4.01±1.76 tC/ha. This report on Sele-Nono forest is higher than other similar moist southwest forests, such as that of Gebre Dima forest, which was estimated at 0.027tC/ha [93], and Gesha-Sayilem forest, which was estimated at 1.27tC/ha [94]. The higher LC reported for Sele-Nono forest signifies the presence of a high diversity of litter types [95], including woody litters (Diameter <10cm) apart from leaf litters that were only considered as litters in Gebre Dima and Gesha-Sayilem forest. Similarly, the LC report in our study is greater than values reported for other montane forests of Ethiopia, such as the Gedo forest (0.41±0.008 tC/ha) as reported by Yohannes et al. [76], Adaba-Dodola community forest (1.06±0.31tC/ha) as reported by Nega et al. [27], Forest belt of Simen Mountains National Park (0.019±0.008tC/ha) as reported by Yelemfrhet and Soromessa [78]. These differences in LC with Sele-Nono forest may be attributed to the difference in vegetation type, which influences the composition of litter falls and its carbon stock. However, the LC of Sele-Nono forest is less compared to the LC estimated for selected church forests in Addis Ababa (4.95tC/ha) [96] and dry montane forests such as Mount Zequalla Monastery (6.5 tC/ha) [26]. This low LC may be linked to the higher moisture content of litter in Sele-Nono forest than in those dry forests. On the other hand, the low LC in our study area may also be due to the high litter decomposition rate as it is a moist forest, which exacerbates litter decomposition more than in dry forests. In relation to this, Sahle et al. [97] have said that biophysical features such as geographic and climatic variation impact the differences in litter biomass and carbon contents of forests.

This study also indicated quite low mean carbon stock density of herbaceous vegetation (HC) (0.96±0.44tC/ha) compared to the other carbon pools in Sele-Nono forest. This may be associated with the fact that the studied forest is highly dominated by tall trees and shrubs having bigger canopies, which compete with the dominance of herbs even if they are rich in their species composition [98]. Moreover, the low HC may be related to the fact that herbaceous vegetation does not have lignified tissues that are supposed to harbor more nutrients, including carbon [99]. In addition, herbaceous vegetation normally has a short life span as many of them are annuals and hence are unable to capture and store carbon for long periods [100]. Because of these facts, some other studies did not include the herbaceous vegetation when they studied the carbon stock of the carbon pools [41,46,76,78,89,101].

The findings in this study also showed that lying dead wood (LDWs) is the third most important carbon pool that contributes mainly to the entire carbon stock of Sele-Nono Forest next to the AGB and BGB. Although LDWs are often neglected in the assessment of the carbon stock of a particular forest [102], it yielded 10.04±6.35tC/ha, equivalent to 11% of the AGC in the current study forest. This high carbon stock value in LDWs in the studied forest may indicate that Sele-Nono forest is likely an old forest [103] that has been involved in mitigating atmospheric carbon dioxide. This result goes with the finding of Keller et al. [104], who reported the carbon stock of LDWs at 16% of the AGC in the eastern Amazonia Forest.

This study estimated the mean soil organic carbon (SOC) stored in Sele-Nono forest at 162.69 tC/ha (ranging from 36.38 tC/ha to 356.49 tC/ha). Nearly similar results were reported in other southwest forests of Ethiopia. For example, the estimated soil carbon stock density of Jimma Forest [62], Bita Forest [39], and Gera Forest [40] were reported at 150 tC/ha, 120 tC/ha, and 95 tC/ha, respectively. Our finding is also slightly higher than the default value of 130tC/ha given by IPCC for volcanic soils [6]. These differences in SOC may be attributed to differences in soil bulk densities, resulting from differences in soil types, vegetation types, and climatic and forest management practices.The mean soil bulk density of Sele-Nono forest was low (1 gcm^-3^, ranging between 0.11 to 5.7 gcm^-3^), which indicates that the study forest has high organic matter (OM) content in the soil [105]. Thus, the higher mean SOC stock may be due to high SOM and fast litter decomposition, which results in maximum storage of soil carbon stock. The carbon concentration (%C) within the Sele-Nono forest soil ranges from 1.22% to 11.37%, with a mean carbon content of 6.03%. This may indicate that the soil of the study forest belongs to the organic soil category rather than mineral soil [106], which could be the consequence of the high drainage of carbon from organic materials of the forest, such as plant and animal residues.

Overall, this study revealed that the soil of the study forest has the largest carbon stocking capacity and was found to store almost twice the carbon stored in AGB. This exactly goes with the Peruvian moist tropical forest result, which had twice as much carbon in the soil as in above-ground biomass [107]. In the same way, the total SOC obtained in the Hanang forest of Tanzania accounted for 72.9% of the total forest carbon [108]. In line with those findings wider survey on the carbon contents of tropical forests reported the occurrence of 2/3 of the terrestrial carbon in soils [6]. These seem reasonable as forest soils are less utilizable than forest woods and may account for higher carbon storage. However, other studies found contradictory results. For example, Djomo et al. [109] suggested that an African moist tropical forest had more than three times as much carbon in above-ground biomass as in soil, whereas Dixon et al. [110] suggested almost equal proportion (1:1) of carbon storage in the entire tropical forests worldwide. The differences between such reports with respect to our current study in Sele-Nono forest might be due to the variation in several factors, including vegetation composition, disturbance history and successional stage, climate, and soil fertility.

This study also reveals not only the variation of carbon stock among carbon pools but also the spatial variation of carbon stock along environmental gradients. This could be because the microclimate associated with the environmental gradients induces variation in soil biophysical features and vegetation cover, ultimately produce a variation in carbon stock quantity. Similar findings were noted elsewhere in Ethiopia [18,24,26,31,46,61,75,76,78] and abroad [82,100].

### Influence of environmental factors on the carbon stock variation

The finding showed a significant variation in carbon stock across different environmental factors. For most forest carbon pools (Soil, AGB, BGB, LDWs, and LB), medium elevation ranges had higher carbon stocks compared to lower and higher elevations. This is likely due to less human interference and the presence of more abundant vegetation with larger diameters and heights in these areas of the forest [42,111]. Other studies have also found higher carbon stocks in soil and tree biomass at medium elevations [31,32,75,76,78]. The study also indicated a clear trend of decreasing carbon stock in AGB, BGB, LDWs, and LB with increasing disturbance levels. This underscores the need for effective forest management to mitigate disturbances and sustain the forest’s climate mitigation potential [112]. Regarding slope, carbon stock in AGB, BGB, LDWs, and LB was often higher in steeper areas than in gentle slopes or flat areas. This may be because steeper areas are less accessible for logging and thinning [42], and consequently preserve the carbon stock. Conversely, gentler slopes and lower elevation parts of the forest are more vulnerable to human activities [42], subsequently reducing their carbon storage capacity.

However, the effects of altitude, slope, and disturbance levels on the carbon stock trends of herbaceous vegetation were found to be opposite to those observed in the other carbon pools. The finding revealed that the HC increases with higher disturbance levels and lower slope levels. This is likely because areas with gentler slopes are more susceptible to human activities, such as selective logging and thinning of larger trees, as reported in previous studies [42]. These conditions may have allowed herbaceous plants to become more abundant in less steep areas compared to steeper areas dominated by trees [37]. Consequently, herbaceous vegetation in less steep (more disturbed) areas would have a higher carbon stock than in steeper (less disturbed) parts of the forest.

### The role of Sele-Nono forest in mitigating climate change

The findings of this study reveal that soils, AGB (especially trees), and LDWs are the most important carbon pools that store most of the carbon in Sele-Nono Forest. Similar results were observed in other studies conducted elsewhere [113]. Even among these three pools, the first two pools (soil and AGB) influence the current overall carbon storage of the forest, which is in concordance with other studies conducted elsewhere in Ethiopia [24,26,27,75,76,78]. However, this result contrasts with the patterns observed by those studies that regarded AGB’s contribution as higher than the soil contribution in the climate change mitigation target. In this current study, the overall carbon stock of Sele-Nono Forest is substantially dependent on the SOC followed by AGC. The contribution of soil to higher total carbon stock density than AGB has also been indicated in other forests of Ethiopia [75,77] and elsewhere outside Ethiopia [114].

In a nutshell, this study indicates that Sele-Nono forest has a considerable effect on mitigating global climate change in general and supports the country’s (Ethiopia) effort toward climate change mitigation. It is estimated that Sele-Nono forest currently sequesters an average of 1045.25 tCO2eq per hectare. The total average carbon stock of this forest was estimated at 284.81±107.81tC/ha. This value seems comparable to Lewis et al. [115] report, which estimated the mean carbon stocks of intact African forests at 202 tC/ha. This may be attributed to the use of quality assurance/quality control tools during the life span of this study for Sele-Nono forest (S2 Appendix). While compared with other studies (Table 9), the mean total carbon stock density of Sele-Nono forest is more comparable with the Gera native moist Afromontane Forest of Ethiopia, which was estimated at 230.09 tC/ha [40] to 336.96 tC/ha [41]. This might be because Gera is structurally and compositionally more similar to Sele-Nono and geographically more proximal than others. The Jimma high forest showed higher carbon stock potential than Sele-Nono; and this might be due to their more intact nature and protection from human interference [62]. In addition, the observed anthropogenic threats, including previous logging and encroachment, could be a reason for the low carbon amounts in Sele-Nono forest compared to the intact Jimma Forest (Table 9).

**Table 9:** Comparison of Sele-Nono forest carbon stock density with some other moist montane forests of the country.

S/N	Moist Afromontane Forest	Author/s	Mean total carbon stock (tC/ha)	Mean total stored Carbon-dioxide (tCO_2(_eq/ha)	Remark (included pools)
1	Sele-Nono	This study	284.81	1045.25	All pools
			106.7	391.58	AGB, BGB
2	Gera	Abaoli and Lemma [40]	230.09	844.4303	AGB, BGB, SOC
		Vanderhaegen *et al*. [41]	336.96	1236.6432	All pools
3	Jimma	De Beenhouwer *et al*.[62]	413	1515.71	All pools
4	Masha/Anderacha/Gesha	EWNRA [89]	76.83	281.9661	AGB, BGB
5	Bale (moist nondegraded forest part	Watson *et al*. [101]	289	1060.63	AGB, BGB
6	Bale (moist degraded forest part	Watson *et al*. [101]	199	730.33	AGB, BGB

However, when the comparison is only on AGB and BGB, the carbon stock of Sele-Nono forest would be more related to the Masha, Anderacha, and Gesha forests. Although Watson’s report on Bale moist Afromontane Forest included only AGB and BGB, its carbon stock looks much higher than the current report on Sele-Nono forest. When comparing only these two pools, the carbon stock of Sele-Nono forest is three times lower than the carbon stock of the un-degraded Bale moist forest and two times lower than the degraded Bale moist forest. This may show that Bale’s moist forests harbor more abundant, bigger, and mature trees than Sele-Nono [101]. In line with this assumption, Grace (2004) claims that forest types with larger trees accumulate more biomass and carbon stock than forests with smaller-sized trees. The differences in their AGC and BGC may also be attributed to the differences in allometric equations used to change the measurable parameters to carbon stocks. Geographical variation between Sele-Nono and Bale Forest could also be another source of factors for the variation in their AGC and BGC report.

### Economic valuation of Sele-Nono forest from its carbon stock potential

The finding of this study reveals that more than 157 million tCO_2_eq have so far been absorbed from the atmosphere by the current stand of Sele-Nono forest. This time, a new business area called carbon market/carbon trade, where units of CO_2_ emission reductions (also known as carbon credits or tons of carbon dioxide equivalents, tCO_2_eq) have become a tradable commodity [116,117]. So, if this large absorption of atmospheric CO_2_ is known by the REDD + projects and by compliance and voluntary carbon markets, they would financially reward the forest/local people for making it a more economically viable land option. This business area arises either because of restrictions to emissions imposed on nations and businesses, or from green businesses and consumers that want to offset their own private emissions.

Upon the sale of the absorbed atmospheric CO_2_, the quantity of financial prize for enhancing the conservation of Sele-Nono forest would be expected to reach more than 1 billion dollars. Hence, the government of Ethiopia has to strive to negotiate this much money of emissions reduction payments from the sale of carbon and should deploy the local people of the district for justifiable forest management and climate change mitigation practices. This suggestion also goes with the climate-resilient growth economy (CRGE) strategy of our country’s developmental agenda as shown by the report of the Ministry of Environment and Forestry of Ethiopia (MoEF) [118]. Similar kinds of business have been exercised elsewhere based on their carbon impacts. For instance, Norway has provided 1 billion US dollars to the government of Indonesia and about 250 million dollars to Guyana in return for conserving their forest for carbon stock purposes [111].

## Conclusions

This study demonstrates that Sele-Nono Forest plays a substantial role in stabilizing atmospheric CO_2_ concentrations. This first quantification of carbon stocks in Sele-Nono Forest indicates significant variation among different carbon pools, with soil containing a larger portion of carbon compared to above-ground biomass. This study also found lying dead woods to be an important source of carbon stock that accounts for 8% of the total biomass. This highlights the need to convert lying dead wood into alternative usable carbon storage forms, such as biochar, before it decays and becomes a source of greenhouse gas emissions. As a baseline research effort for the Sele-Nono Forest, this study suggests that periodic assessments should be conducted to evaluate the impact of possible land use changes on biomass carbon stock and soil organic carbon (SOC) dynamics.

## Supporting information

S1 Appendix Geographical grids drawn on the map of Sele-Nono forest to establish the location of sample plots (the map is reprinted from Kefalew et al. [42] under a CC BY license, with permission from [Springer Nature], and with the original copyright [2022]). (JPG)

S2 Appendix Method of quality assurance/quality control implemented during the life span of this study in Sele-Nono forest. (DOC)

S3 Appendix Nested sample plot type (shape and size) used for the measurement of trees in the study area (modified from SOP plot design of Walker et al. [45]). (JPG)

S4 Appendix Plot design within the 25 m X 25 m nest plots for shrubs and soil data. (JPG)

S5 Appendix Example of locating four plots of size 1 m X 1 m radiating 100 m away from the center of the main plots for collecting data related to herbs and litter biomass. (JPG)

S6 Appendix Example of layout of line transects method used on flat terrain to estimate the volume of lying dead woods in Sele-Nono Forest. (JPG)

S1 Table Corrected projected horizontal length of plots and/or nested subplots for various slope gradients. (DOC)

S2 Table Biomass, Carbon stored, and amount of carbon dioxide removed by AGB components in Sele-Nono forest. (DOC)

S3 Table Wood density determination for all three classes of lying dead woods (LDWs) in Sele-Nono forest. (DOC)

S4 Table Total biomass, carbon stock, and soil organic carbon of Sele-Nono forest at plot level (t/ha). (DOC)

S5 Table Bulk density calculation and soil organic carbon storage in Sele-Nono forest at each sample plots. (DOC)
