# Supplementary figures and images for: Carbon stock quantification and climate mitigation potential of a tropical moist forest in Ethiopia
Source: PLoS One. 2025 Jan 24;20(1):e0316886. doi: 10.1371/journal.pone.0316886 (PMC11760618; doi:10.1371/journal.pone.0316886)

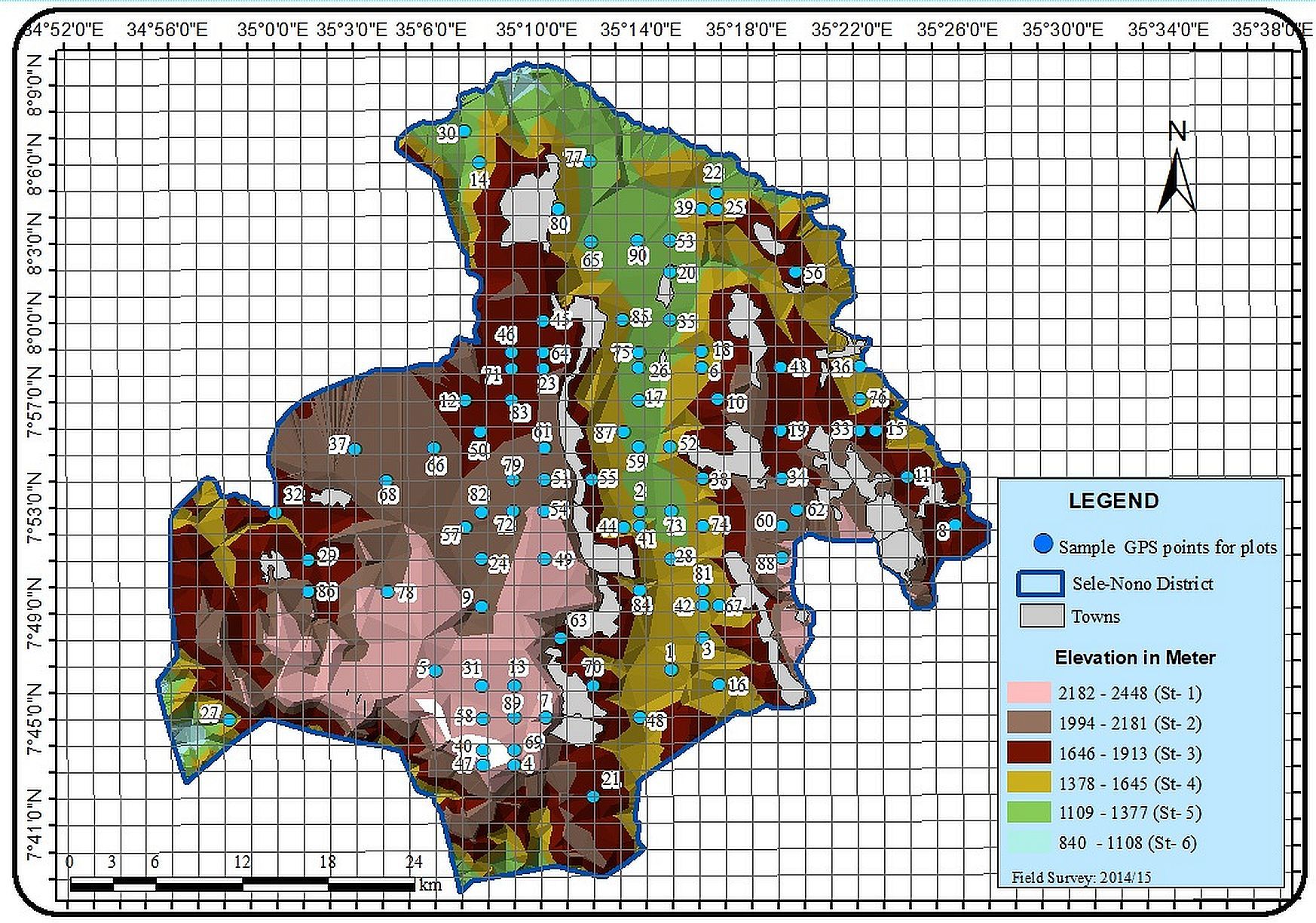

Supplement: S1 Appendix — (JPG) [file pone.0316886.s001.jpg]

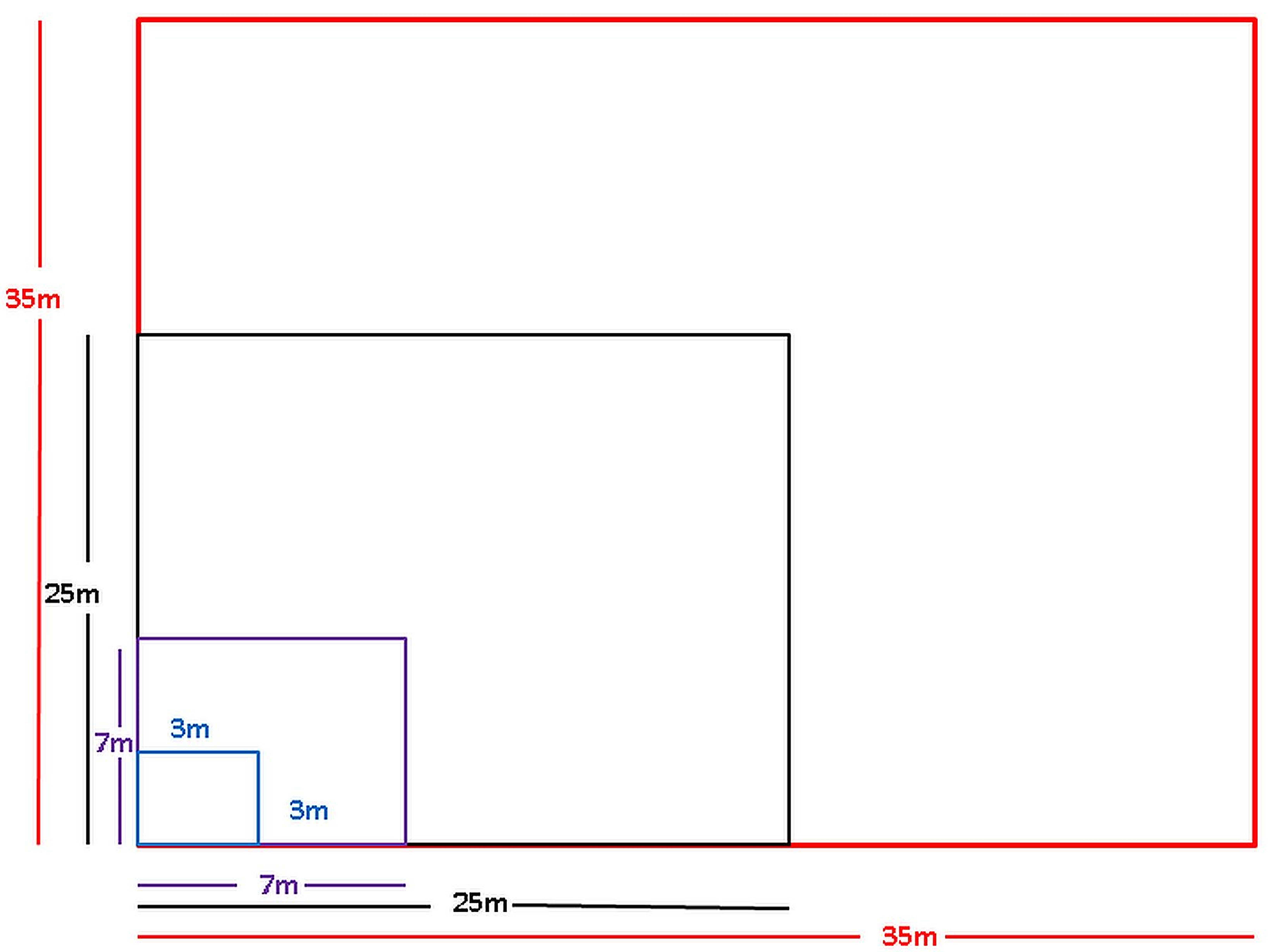

Supplement: S3 Appendix — (JPG) [file pone.0316886.s003.jpg]

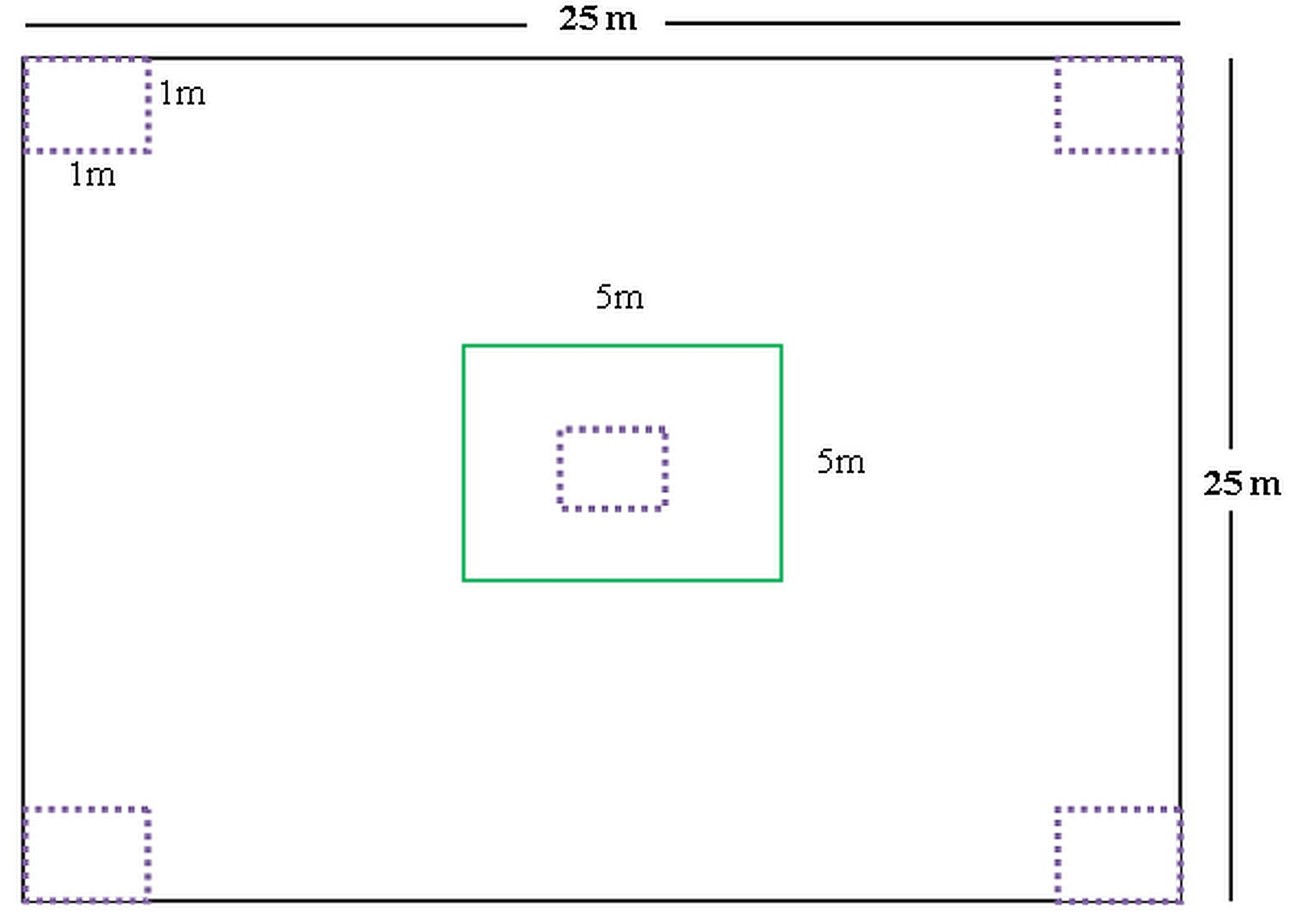

Supplement: S4 Appendix — (JPG) [file pone.0316886.s004.jpg]

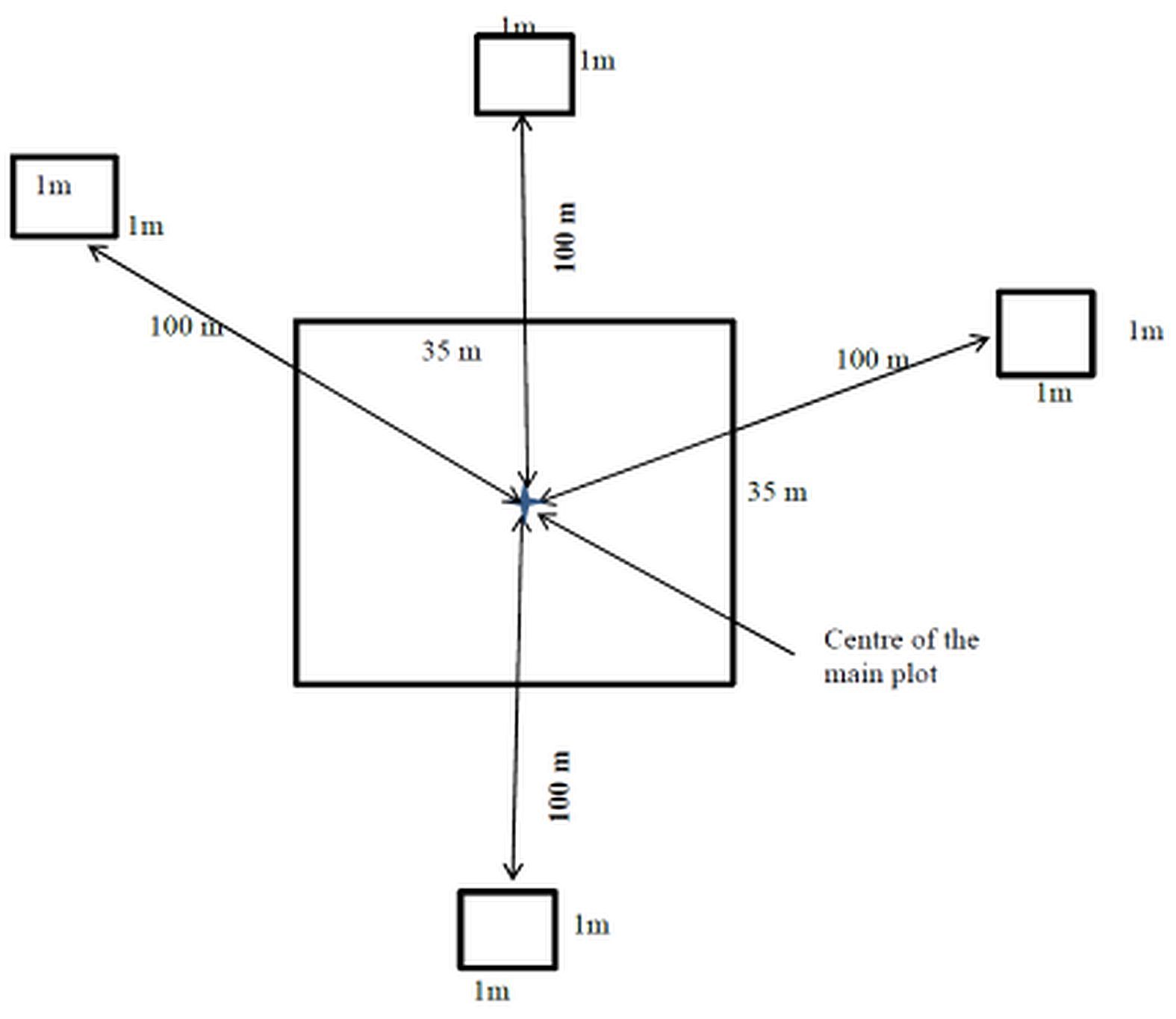

Supplement: S5 Appendix — (JPG) [file pone.0316886.s005.jpg]

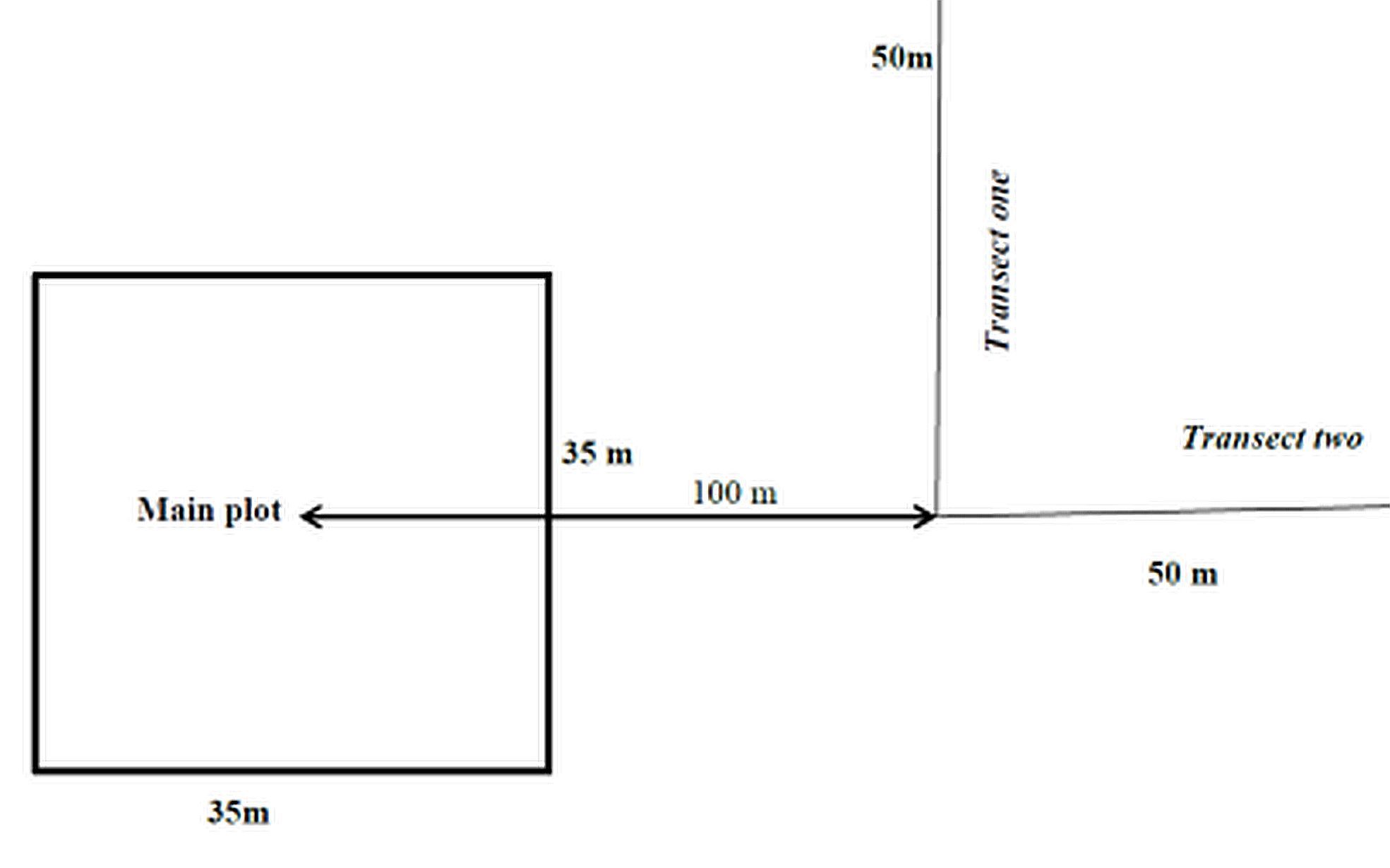

Supplement: S6 Appendix — (JPG) [file pone.0316886.s006.jpg]
