# Supplementary material for: Carbon stock quantification and climate mitigation potential of a tropical moist forest in Ethiopia
Source: PLoS One. 2025 Jan 24;20(1):e0316886. doi: 10.1371/journal.pone.0316886 (PMC11760618; doi:10.1371/journal.pone.0316886)
Supplement: S2 Appendix — (DOC) [file pone.0316886.s002.doc]

S2 Appendix: Method of quality assurance/quality control implemented during the life span of this study in Sele-Nono forest

| S/N | Kind of error | Source of error | Actions applied to reduce the error |
| --- | --- | --- | --- |
| 1 | Sampling error | Selection of forest plots | The shape and size of plots for each component of the carbon pools were determined based on a design created by a group of scientists [46]. The plots were strategically placed using a stratified sampling method to ensure accurate representation of the forest [45, 53]. |
| 2 | Field measurement error | Measurements of relevant parameters for all C-pools | Researchers and District administrators hired literate crew members and trained them to accurately collect data, such as DBH, in the field. A pilot field measurement was conducted near the study forest to evaluate the training's effectiveness. Potential errors were identified and corrected before the actual data collection in Sele-Nono Forest. Measures were taken to ensure that all carbon pools were assessed using state-of-the-art methods, avoiding double-counting of trees or mistaking dead trees for living ones. |
| 3 | Data taking and entry error | Filling the data sheet and entering them into a computer | All the field data recordings were made by the researcher with great patience and care. Data entry was also made by the researcher himself immediately after a return to the office. Data entry for each plot was rechecked with the original hard copy data sheet before we entered data for the next plot. |
| 4 | Estimation error | Application of allometric equations | We validated the models using local data to identify those most suitable for the study area, selecting the ones with the lowest error bias [62]. The errors associated with the allometric equation we employed in this study were estimated less than 10% [62]. |
|  |  | Application of wet-dry mass ratio technique for biomass estimation | All balances used for measuring sub-sample fresh weights in the field and dry weights in the lab were calibrated using known weights. |
|  |  | Biomass estimation at the plot level | Biomass density at the plot level was estimated using appropriate techniques. The conversion to a per-hectare basis was performed using slope-corrected areas to report biomass on horizontal projection levels, following the SOP manual by [46]. |
|  |  | Application biomass to  carbon ratio | The IPCC [8] provides a default value of 0.47 for tropical and subtropical forests, with an interval estimate ranging from 0.44 to 0.49. This represents an improvement over the previously suggested value of 0.5 by many authors. |
