# Supplementary material for: Carbon stock quantification and climate mitigation potential of a tropical moist forest in Ethiopia
Source: PLoS One. 2025 Jan 24;20(1):e0316886. doi: 10.1371/journal.pone.0316886 (PMC11760618; doi:10.1371/journal.pone.0316886)

**S1 Table**: Corrected projected horizontal length of plots and/or nested subplots for various slope gradients (Ls is the corrected length of the plot and/or subplot parallel to the slope on the ground, and L_field_ is correct lying dead wood transect for various slope gradients based on a 50 m transect on flat ground).

| Slope  degree (θ) | Cos (θ) | Plot area correction due to slope=L_s_ X L, where L_s_=L_field_ X cos(θ) | | | | | | | | | | | | 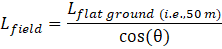 |
| --- | --- | --- | --- | --- | --- | --- | --- | --- | --- | --- | --- | --- | --- | --- |
|  |  | Sapling  (3 m * 3 m) | | Shrub  (5 m * 5 m) | | Liana  (7 m * 7 m) | | Small tree  (7 m * 7 m) | | Medium tree  (25 m * 25 m) | | Large tree  (35 m * 35 m) | |  |
|  |  | L_s_ | Area | L_s_ | Area | L_s_ | Area | L_s_ | Area | L_s_ | Area | L_s_ | Area |  |
| 10 | 0.99 | 2.97 | 8.91 | 4.95 | 24.7 | 6.93 | 48.5 | 6.93 | 48.5 | 24.75 | 618.7 | 34.6 | 1212.7 | 50.50 |
| 15 | 0.97 | 2.91 | 8.73 | 4.85 | 24.2 | 6.79 | 47.5 | 6.79 | 47.5 | 24.25 | 606.2 | 33.9 | 1188.2 | 51.54 |
| 20 | 0.94 | 2.82 | 8.46 | 4.7 | 23.5 | 6.58 | 46.0 | 6.58 | 46.0 | 23.5 | 587.5 | 32.9 | 1151.5 | 53.19 |
| 25 | 0.91 | 2.73 | 8.19 | 4.55 | 22.7 | 6.37 | 44.5 | 6.37 | 44.5 | 22.75 | 568.7 | 31.8 | 1114.7 | 54.94 |
| 30 | 0.87 | 2.61 | 7.83 | 4.35 | 21.7 | 6.09 | 42.6 | 6.09 | 42.6 | 21.75 | 543.7 | 30.4 | 1065.7 | 57.47 |
| 35 | 0.81 | 2.43 | 7.29 | 4.05 | 20.2 | 5.67 | 39.6 | 5.67 | 39.6 | 20.25 | 506.2 | 28.3 | 992.2 | 61.72 |
| 40 | 0.76 | 2.28 | 6.84 | 3.8 | 19 | 5.32 | 37.2 | 5.32 | 37.2 | 19 | 475 | 26.6 | 931 | 65.78 |
| 45 | 0.7 | 2.1 | 6.3 | 3.5 | 17.5 | 4.9 | 34.3 | 4.9 | 34.3 | 17.5 | 437.5 | 24.5 | 857.5 | 71.42 |
| 50 | 0.64 | 1.92 | 5.76 | 3.2 | 16 | 4.48 | 31.3 | 4.48 | 31.3 | 16 | 400 | 22.4 | 784 | 78.12 |
| 55 | 0.57 | 1.71 | 5.13 | 2.85 | 14.2 | 3.99 | 27.9 | 3.99 | 27.9 | 14.25 | 356.2 | 19.9 | 698.2 | 87.71 |
| 60 | 0.5 | 1.5 | 4.5 | 2.5 | 12.5 | 3.5 | 24.5 | 3.5 | 24.5 | 12.5 | 312.5 | 17.5 | 612.5 | 100 |
| 65 | 0.42 | 1.26 | 3.78 | 2.1 | 10.5 | 2.94 | 20.5 | 2.94 | 20.5 | 10.5 | 262.5 | 14.7 | 514.5 | 119.04 |
| 70 | 0.34 | 1.02 | 3.06 | 1.7 | 8.5 | 2.38 | 16.6 | 2.38 | 16.6 | 8.5 | 212.5 | 11.9 | 416.5 | 147.05 |
| 75 | 0.25 | 0.75 | 2.25 | 1.25 | 6.25 | 1.75 | 12.2 | 1.75 | 12.2 | 6.25 | 156.2 | 8.75 | 306.2 | 200 |
| 80 | 0.17 | 0.51 | 1.53 | 0.85 | 4.25 | 1.19 | 8.33 | 1.19 | 8.33 | 4.25 | 106.2 | 5.95 | 208.2 | 294.11 |
| 85 | 0.08 | 0.24 | 0.72 | 0.4 | 2 | 0.56 | 3.92 | 0.56 | 3.92 | 2 | 50 | 2.8 | 98 | 625 |

Then, these slopes corrected area would be extrapolated to a hectare basis during data analysis using the following scaling factor.


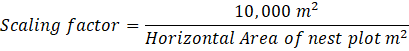

Supplement: S1 Table — (DOC) [file pone.0316886.s007.doc]
