# Supplementary material for: Carbon stock quantification and climate mitigation potential of a tropical moist forest in Ethiopia
Source: PLoS One. 2025 Jan 24;20(1):e0316886. doi: 10.1371/journal.pone.0316886 (PMC11760618; doi:10.1371/journal.pone.0316886)
Supplement: S2 Table — (DOC) [file pone.0316886.s008.doc]

**S2 Table**: Biomass, Carbon stored, and amount of carbon dioxide removed by AGB components in Sele-Nono Forest

| **S/N** | **Species** | **AGB**  **components** | **Abundance**  **(individuals** | **Av.**  **DBH** | **Av.**  **H** | **Av .wood**  **density** | **Av.AGB (t/indv.)** | **Av.AGC**  **(t/indv.)** | **Av.tCO2 eq./indv.** | **Av.AGB (t/sp.)** | **Av.AGC**  **(t/sp.)** | **Av.tCO2**  **eq./sp** |
| --- | --- | --- | --- | --- | --- | --- | --- | --- | --- | --- | --- | --- |
| **1** | *Schefflera abyssinica* | T | 65 | 124.8 | 64.13 | 0.43 | 21.17 | 9.95 | 36.52 | 1376.29 | 646.85 | 2373.96 |
| **2** | *Ficus vasta* | T | 30 | 106.4 | 57.80 | 0.63 | 20.34 | 9.56 | 35.09 | 610.30 | 286.84 | 1052.71 |
| **3** | *Schefflera volkensii* | T | 28 | 95.46 | 53.86 | 0.55 | 13.46 | 6.32 | 23.21 | 376.78 | 177.09 | 649.90 |
| **4** | *Ficus ovata* | T | 24 | 96.38 | 54.20 | 0.5 | 12.57 | 5.91 | 21.68 | 301.66 | 141.78 | 520.33 |
| **5** | *Manilkara butugi* | T | 70 | 47.39 | 34.13 | 0.92 | 3.63 | 1.71 | 6.26 | 254.13 | 119.44 | 438.35 |
| **6** | *Olea welwitschii* | T | 72 | 48.42 | 34.61 | 0.78 | 3.27 | 1.54 | 5.64 | 235.23 | 110.56 | 405.74 |
| **7** | *Pouteria adolfi-friederici* | T | 108 | 40.62 | 30.87 | 0.68 | 1.81 | 0.85 | 3.13 | 195.89 | 92.07 | 337.89 |
| **8** | *Trilepisium madagascariense* | T | 93 | 46.4 | 33.66 | 0.5 | 1.90 | 0.89 | 3.27 | 176.30 | 82.86 | 304.10 |
| **9** | *Morus mesozygia* | T | 88 | 42.6 | 31.84 | 0.58 | 1.76 | 0.83 | 3.03 | 154.57 | 72.65 | 266.62 |
| **10** | *Deinbollia kilimandscharica* | T | 67 | 42.5 | 31.79 | 0.7 | 2.10 | 0.99 | 3.62 | 140.54 | 66.05 | 242.42 |
| **11** | *Ekebergia capensis* | T | 49 | 46.3 | 33.62 | 0.75 | 2.80 | 1.32 | 4.83 | 137.22 | 64.49 | 236.69 |
| **12** | *Croton macrostachyus* | T | 81 | 42.17 | 31.63 | 0.54 | 1.60 | 0.75 | 2.75 | 129.26 | 60.75 | 222.95 |
| **13** | *Syzygium guineense Subsp. afromontanum* | T | 74 | 38.4 | 29.76 | 0.7 | 1.61 | 0.76 | 2.78 | 119.38 | 56.11 | 205.92 |
| **14** | *Hallea rubrostipulata* | T | 56 | 46.6 | 33.76 | 0.55 | 2.10 | 0.99 | 3.63 | 117.81 | 55.37 | 203.21 |
| **15** | *Ilex mitis* | T | 68 | 40.3 | 30.71 | 0.65 | 1.70 | 0.80 | 2.93 | 115.63 | 54.35 | 199.46 |
| **16** | *Ficus sur* | T | 37 | 53.7 | 37.03 | 0.5 | 2.77 | 1.30 | 4.77 | 102.37 | 48.11 | 176.58 |
| **17** | *Albizia gummifera* | T | 79 | 36.2 | 28.64 | 0.6 | 1.19 | 0.56 | 2.06 | 94.12 | 44.24 | 162.35 |
| **18** | *Elaeodendron buchananii* | T | 103 | 33.5 | 27.23 | 0.55 | 0.90 | 0.42 | 1.54 | 92.24 | 43.35 | 159.10 |
| **19** | *Ficus thonningii* | T | 52 | 44.51 | 32.76 | 0.47 | 1.60 | 0.75 | 2.76 | 83.33 | 39.17 | 143.74 |
| **20** | *Trichilia dregeana* | T | 39 | 42.5 | 31.79 | 0.65 | 1.95 | 0.92 | 3.37 | 76.10 | 35.77 | 131.26 |
| **21** | *Ficus sycomorus* | T | 23 | 58.27 | 39.05 | 0.48 | 3.28 | 1.54 | 5.67 | 75.54 | 35.51 | 130.30 |
| **22** | *Macaranga capensis* | T | 66 | 34.2 | 27.60 | 0.65 | 1.11 | 0.52 | 1.92 | 73.39 | 34.50 | 126.60 |
| **23** | *Alstonia boonei* | T | 87 | 37.1 | 29.10 | 0.36 | 0.77 | 0.36 | 1.33 | 67.09 | 31.53 | 115.72 |
| **24** | *Milicia excelsa* | T | 12 | 58.6 | 39.19 | 0.78 | 5.35 | 2.52 | 9.23 | 64.24 | 30.19 | 110.80 |
| **25** | *Bersama abyssinica* | T | 63 | 32.1 | 26.48 | 0.7 | 1.01 | 0.48 | 1.75 | 63.92 | 30.04 | 110.26 |
| **26** | *Diospyros abyssinica* | T | 58 | 31.7 | 26.27 | 0.72 | 1.01 | 0.47 | 1.74 | 58.56 | 27.52 | 101.01 |
| **27** | *Celtis africana* | T | 57 | 30.67 | 25.71 | 0.72 | 0.93 | 0.44 | 1.60 | 52.83 | 24.83 | 91.13 |
| **28** | *Dombeya torrida* | T | 34 | 38.57 | 29.85 | 0.54 | 1.27 | 0.60 | 2.18 | 43.07 | 20.24 | 74.29 |
| **29** | *Apodytes dimidiata* | T | 73 | 24.6 | 22.27 | 0.72 | 0.52 | 0.25 | 0.90 | 38.24 | 17.97 | 65.96 |
| **30** | *Sapium ellipticum* | T | 69 | 28.42 | 24.46 | 0.52 | 0.55 | 0.26 | 0.96 | 38.22 | 17.96 | 65.93 |
| **31** | *Allophylus abyssinicus* | T | 58 | 24.6 | 22.27 | 0.9 | 0.65 | 0.31 | 1.12 | 37.77 | 17.75 | 65.16 |
| **32** | *Garcinia ovalifolia* | T | 58 | 27.44 | 23.91 | 0.67 | 0.65 | 0.30 | 1.12 | 37.57 | 17.66 | 64.81 |
| **33** | *Albizia grandibracteata* | T | 41 | 31.6 | 26.21 | 0.61 | 0.85 | 0.40 | 1.47 | 34.92 | 16.41 | 60.24 |
| **34** | *Prunus africana* | T | 51 | 26.68 | 23.48 | 0.75 | 0.67 | 0.32 | 1.16 | 34.30 | 16.12 | 59.16 |
| **35** | *Baphia abyssinica* | T | 30 | 28.9 | 24.73 | 1 | 1.10 | 0.51 | 1.89 | 32.85 | 15.44 | 56.67 |
| **36** | *Cordia africana* | T | 28 | 36.4 | 28.74 | 0.54 | 1.09 | 0.51 | 1.88 | 30.53 | 14.35 | 52.66 |
| **37** | *Fagaropsis angolensis* | T | 27 | 35.2 | 28.12 | 0.6 | 1.11 | 0.52 | 1.91 | 29.92 | 14.06 | 51.61 |
| **38** | *Mimusops kummel* | T | 38 | 26.8 | 23.54 | 0.86 | 0.78 | 0.37 | 1.34 | 29.55 | 13.89 | 50.97 |
| **39** | *Pittosporum viridiflorum* | T | 53 | 25.8 | 22.97 | 0.65 | 0.54 | 0.25 | 0.92 | 28.42 | 13.36 | 49.02 |
| **40** | *Lepidotrichilia volkensii* | T | 61 | 26.5 | 23.37 | 0.52 | 0.46 | 0.22 | 0.80 | 28.20 | 13.25 | 48.64 |
| **41** | *Anthocleista schweinfurthii* | T | 24 | 38.4 | 29.76 | 0.5 | 1.16 | 0.55 | 2.00 | 27.88 | 13.10 | 48.09 |
| **42** | *Trema orientalis* | T | 59 | 24.59 | 22.26 | 0.6 | 0.44 | 0.21 | 0.76 | 25.84 | 12.15 | 44.57 |
| **43** | *Chionanthus mildbraedii* | T | 44 | 23.3 | 21.49 | 0.9 | 0.57 | 0.27 | 0.98 | 24.90 | 11.70 | 42.95 |
| **44** | *Albizia schimperiana* | T | 26 | 30.36 | 25.54 | 0.7 | 0.88 | 0.41 | 1.52 | 22.84 | 10.73 | 39.39 |
| **45** | *Strychnos mitis* | T | 55 | 22.3 | 20.89 | 0.67 | 0.38 | 0.18 | 0.65 | 20.83 | 9.79 | 35.93 |
| **46** | *Polyscias fulva* | T | 75 | 20.62 | 19.85 | 0.55 | 0.26 | 0.12 | 0.44 | 19.13 | 8.99 | 33.00 |
| **47** | *Ocotea kenyensis* | T | 16 | 31 | 25.89 | 0.9 | 1.18 | 0.56 | 2.04 | 18.96 | 8.91 | 32.70 |
| **48** | *Brucea antidysenterica* | T | 31 | 28.4 | 24.45 | 0.55 | 0.58 | 0.27 | 1.01 | 18.11 | 8.51 | 31.23 |
| **49** | *Celtis philippensis* | T | 25 | 25.8 | 22.97 | 0.86 | 0.70 | 0.33 | 1.22 | 17.62 | 8.28 | 30.39 |
| **50** | *Celtis gomphophylla* | T | 33 | 27.4 | 23.89 | 0.54 | 0.52 | 0.25 | 0.90 | 17.25 | 8.11 | 29.76 |
| **51** | *Ritchiea albersii Gilg.* | T | 44 | 23.1 | 21.37 | 0.55 | 0.34 | 0.16 | 0.59 | 15.06 | 7.08 | 25.97 |
| **52** | *Phoenix reclinata* | Palm | 81 | 23.4 | 16.40 | 0.55 | 0.19 | 0.09 | 0.32 | 15.02 | 7.06 | 25.91 |
| **53** | *Celtis toka* | T | 18 | 32.51 | 26.70 | 0.55 | 0.83 | 0.39 | 1.43 | 14.92 | 7.01 | 25.73 |
| **54** | *Garcinia buchananii* | T | 54 | 18.92 | 18.77 | 0.67 | 0.25 | 0.12 | 0.43 | 13.37 | 6.28 | 23.06 |
| **55** | *Combretum molle* | T | 8 | 41.7 | 31.40 | 0.55 | 1.58 | 0.74 | 2.72 | 12.63 | 5.93 | 21.78 |
| **56** | *Pouteria altissima* | T | 8 | 38.9 | 30.01 | 0.66 | 1.58 | 0.74 | 2.72 | 12.60 | 5.92 | 21.74 |
| **57** | *Lecaniodiscus fraxinifolius* | T | 27 | 22.6 | 21.07 | 0.79 | 0.46 | 0.22 | 0.79 | 12.43 | 5.84 | 21.45 |
| **58** | *Millettia ferruginea* | T | 46 | 17.2 | 17.64 | 0.85 | 0.24 | 0.11 | 0.42 | 11.22 | 5.28 | 19.36 |
| **59** | *Sterculia africana* | T | 3 | 58.2 | 39.02 | 0.55 | 3.74 | 1.76 | 6.45 | 11.22 | 5.27 | 19.35 |
| **60** | *Euphorbia ampliphylla* | T | 19 | 32 | 26.43 | 0.4 | 0.58 | 0.27 | 1.01 | 11.08 | 5.21 | 19.10 |
| **61** | *Vepris dainellii* | T | 83 | 14.37 | 15.69 | 0.73 | 0.13 | 0.06 | 0.23 | 10.96 | 5.15 | 18.91 |
| **62** | *Pouteria alnifolia* | T | 22 | 28.7 | 24.62 | 0.42 | 0.46 | 0.22 | 0.80 | 10.15 | 4.77 | 17.50 |
| **63** | *Combretum adenogonium* | T | 7 | 38.4 | 29.76 | 0.55 | 1.27 | 0.60 | 2.20 | 8.92 | 4.19 | 15.39 |
| **64** | *Dracaena steudneri* | T | 45 | 18.49 | 18.49 | 0.55 | 0.19 | 0.09 | 0.33 | 8.66 | 4.07 | 14.93 |
| **65** | *Erythrina brucei* | T | 6 | 38.6 | 29.86 | 0.55 | 1.29 | 0.61 | 2.23 | 7.75 | 3.64 | 13.37 |
| **66** | *Blighia unijugata* | T | 43 | 17.4 | 17.77 | 0.6 | 0.18 | 0.08 | 0.31 | 7.70 | 3.62 | 13.27 |
| **67** | *Stereospermum kunthianum* | T | 13 | 28.37 | 24.43 | 0.55 | 0.58 | 0.27 | 1.00 | 7.57 | 3.56 | 13.06 |
| **68** | *Allophylus macrobotrys* | T | 25 | 21.8 | 20.58 | 0.55 | 0.29 | 0.14 | 0.51 | 7.36 | 3.46 | 12.70 |
| **69** | *Erythrina abyssinica* | T | 4 | 42.38 | 31.73 | 0.61 | 1.82 | 0.86 | 3.14 | 7.28 | 3.42 | 12.56 |
| **70** | *Antiaris toxicaria* | T | 11 | 30.48 | 25.60 | 0.5 | 0.64 | 0.30 | 1.10 | 7.03 | 3.30 | 12.12 |
| **71** | *Olea capensis* | T | 35 | 16.2 | 16.96 | 0.81 | 0.20 | 0.09 | 0.34 | 6.98 | 3.28 | 12.04 |
| **72** | *Cassipourea malosana* | T | 22 | 20.18 | 19.57 | 0.72 | 0.31 | 0.15 | 0.54 | 6.90 | 3.24 | 11.91 |
| **73** | *Urera hypselodendron* | L | 41 | 11.83 |  |  | 0.16 | 0.08 | 0.28 | 6.59 | 3.10 | 11.38 |
| **74** | *Alangium chinense* | T | 21 | 24.2 | 22.03 | 0.4 | 0.28 | 0.13 | 0.49 | 5.94 | 2.79 | 10.25 |
| **75** | *Maytenus arbutifolia* | T | 32 | 17.4 | 17.77 | 0.55 | 0.16 | 0.08 | 0.28 | 5.26 | 2.47 | 9.07 |
| **76** | *Ficus exasperata* | T | 26 | 20.3 | 19.65 | 0.45 | 0.20 | 0.09 | 0.35 | 5.24 | 2.46 | 9.03 |
| **77** | *Celtis zenkeri* | T | 21 | 18.4 | 18.43 | 0.72 | 0.25 | 0.12 | 0.43 | 5.19 | 2.44 | 8.95 |
| **78** | *Maesa lanceolata* | T | 67 | 12.8 | 14.55 | 0.55 | 0.07 | 0.03 | 0.13 | 4.98 | 2.34 | 8.58 |
| **79** | *Dracaena afromontana* | T | 152 | 8.94 | 11.52 | 0.55 | 0.03 | 0.01 | 0.05 | 4.46 | 2.10 | 7.69 |
| **80** | *Cyathea manniana* | T | 68 | 13.5 | 15.06 | 0.4 | 0.06 | 0.03 | 0.11 | 4.25 | 2.00 | 7.33 |
| **81** | *Kigelia africana* | T | 5 | 31 | 25.89 | 0.55 | 0.73 | 0.34 | 1.26 | 3.66 | 1.72 | 6.32 |
| **82** | *Lannea schimperi* | T | 16 | 22.3 | 20.89 | 0.4 | 0.23 | 0.11 | 0.39 | 3.66 | 1.72 | 6.32 |
| **83** | Standing dead tree (type 3) | SDT | 77 | 17 | 6.00 | 0.55 | 0.05 | 0.02 | 0.08 | 3.66 | 1.72 | 6.31 |
| **84** | *Nuxia congesta* | T | 14 | 21.4 | 20.33 | 0.5 | 0.26 | 0.12 | 0.44 | 3.58 | 1.68 | 6.18 |
| **85** | *Ehertia cymosa* | L | 24 | 10.28 |  |  | 0.11 | 0.05 | 0.19 | 2.66 | 1.25 | 4.59 |
| **86** | *Zanthoxylum usambarense* | T | 8 | 20.38 | 19.70 | 0.69 | 0.31 | 0.15 | 0.53 | 2.47 | 1.16 | 4.26 |
| **87** | *Lannea schweinfurthii* | T | 13 | 20.5 | 19.77 | 0.4 | 0.18 | 0.09 | 0.32 | 2.39 | 1.13 | 4.13 |
| **88** | *Teclea noblis* | T | 58 | 9.3 | 11.82 | 0.7 | 0.04 | 0.02 | 0.07 | 2.38 | 1.12 | 4.11 |
| **89** | *Lobelia giberroa* | T | 21 | 14.9 | 16.06 | 0.55 | 0.11 | 0.05 | 0.19 | 2.31 | 1.09 | 3.99 |
| **90** | *Phytolacca dodecandra* | L | 29 | 8.42 |  |  | 0.07 | 0.03 | 0.11 | 1.89 | 0.89 | 3.26 |
| **91** | *Pseudocedrela kotschyi* | T | 4 | 25.6 | 22.85 | 0.55 | 0.45 | 0.21 | 0.77 | 1.79 | 0.84 | 3.08 |
| **92** | Standing dead tree (stumps) | SDT | 78 | 30 | 0.50 | 0.55 | 0.02 | 0.01 | 0.03 | 1.52 | 0.71 | 2.61 |
| **93** | *Galiniera saxifraga* | T | 24 | 11.32 | 13.43 | 0.55 | 0.05 | 0.03 | 0.09 | 1.30 | 0.61 | 2.24 |
| **94** | *Catha edulis* | T | 2 | 28.3 | 24.39 | 0.55 | 0.58 | 0.27 | 1.00 | 1.16 | 0.54 | 2.00 |
| **95** | *Embelia schimperi* | L | 19 | 7.9 |  |  | 0.06 | 0.03 | 0.09 | 1.05 | 0.49 | 1.80 |
| **96** | *Dalbergia lactea* | L | 43 | 5.7 |  |  | 0.02 | 0.01 | 0.04 | 0.99 | 0.47 | 1.71 |
| **97** | Standing dead tree (type 2) | SDT | 62 | 19 | 18.82 | 0.55 | 0.01 | 0.01 | 0.03 | 0.92 | 0.43 | 1.58 |
| **98** | *Psychotria orophila* | T | 44 | 7.35 | 10.14 | 0.55 | 0.02 | 0.01 | 0.03 | 0.78 | 0.37 | 1.34 |
| **99** | *Bamboo* | B | 109 | 5.9 |  |  | 0.01 | 0.00 | 0.01 | 0.76 | 0.36 | 1.31 |
| **100** | Standing dead tree (type 1) | SDT | 23 | 24 | 21.91 | 0.55 | 0.03 | 0.02 | 0.06 | 0.75 | 0.35 | 1.30 |
| **101** | *Canthium oligocarpum* | T | 34 | 7.1 | 9.91 | 0.7 | 0.02 | 0.01 | 0.04 | 0.70 | 0.33 | 1.20 |
| **102** | *Combretum paniculatum* | L | 30 | 5.7 |  |  | 0.02 | 0.01 | 0.04 | 0.69 | 0.33 | 1.20 |
| **103** | *Paullinia pinnata* | L | 25 | 6.1 |  |  | 0.03 | 0.01 | 0.05 | 0.69 | 0.33 | 1.19 |
| **104** | *Maerua oblongifolia* | T | 5 | 15.3 | 16.34 | 0.55 | 0.12 | 0.06 | 0.20 | 0.59 | 0.28 | 1.02 |
| **105** | *Gouania longispicta* | L | 28 | 5.5 |  |  | 0.02 | 0.01 | 0.04 | 0.59 | 0.28 | 1.02 |
| **106** | *Dioscorea praehensilis* | L | 28 | 5.2 |  |  | 0.02 | 0.01 | 0.03 | 0.51 | 0.24 | 0.87 |
| **107** | *Rothmannia urcelliformis* | T | 52 | 5.22 | 8.11 | 0.6 | 0.01 | 0.00 | 0.01 | 0.41 | 0.19 | 0.71 |
| **108** | *Coffea arabica* | T | 52 | 5.33 | 8.22 | 0.55 | 0.01 | 0.00 | 0.01 | 0.40 | 0.19 | 0.69 |
| **109** | *Oxyanthus speciosus* | T | 38 | 5.8 | 8.69 | 0.55 | 0.01 | 0.00 | 0.02 | 0.36 | 0.17 | 0.63 |
| **110** | *Saba comorensis* | L | 21 | 5 |  |  | 0.02 | 0.01 | 0.03 | 0.34 | 0.16 | 0.59 |
| **111** | *Hippocratea pallens* | L | 11 | 6.3 |  |  | 0.03 | 0.01 | 0.05 | 0.33 | 0.16 | 0.57 |
| **112** | *Clerodendrum myricoides* | L | 27 | 4.24 |  |  | 0.01 | 0.00 | 0.02 | 0.28 | 0.13 | 0.49 |
| **113** | *Bridelia micranta* | T | 33 | 5.12 | 8.01 | 0.67 | 0.01 | 0.00 | 0.01 | 0.28 | 0.13 | 0.48 |
| **114** | *Hippocratea goetzei* | L | 18 | 4.8 |  |  | 0.01 | 0.01 | 0.03 | 0.26 | 0.12 | 0.45 |
| **115** | *Hippocratea africana* | L | 16 | 5 |  |  | 0.02 | 0.01 | 0.03 | 0.26 | 0.12 | 0.45 |
| **116** | *Jasminum abyssinicum* | L | 37 | 3.6 |  |  | 0.01 | 0.00 | 0.01 | 0.25 | 0.12 | 0.44 |
| **117** | *Gardenia ternifolia* | T | 25 | 5.6 | 8.49 | 0.55 | 0.01 | 0.00 | 0.02 | 0.22 | 0.10 | 0.38 |
| **118** | *Cyathea manniana* | Tree fern | 72 | 8.44 | 2.30 |  | 0.00 | 0.00 | 0.01 | 0.22 | 0.10 | 0.37 |
| **119** | *Schefflera myriantha* | L | 8 | 6 |  |  | 0.03 | 0.01 | 0.05 | 0.21 | 0.10 | 0.37 |
| **120** | *Maytenus gracilipes* | T | 55 | 3.9 | 6.71 | 0.55 | 0.00 | 0.00 | 0.01 | 0.19 | 0.09 | 0.33 |
| **121** | *Tiliacora troupinii* | L | 31 | 3.4 |  |  | 0.01 | 0.00 | 0.01 | 0.18 | 0.09 | 0.31 |
| **122** | *Terminalia schimperiana* | T | 4 | 10.33 | 12.65 | 0.48 | 0.04 | 0.02 | 0.06 | 0.15 | 0.07 | 0.26 |
| **123** | *Vernonia auriculifera* | T | 38 | 3.87 | 6.67 | 0.55 | 0.00 | 0.00 | 0.01 | 0.13 | 0.06 | 0.22 |
| **124** | *Rhamnus prinoides* | T | 11 | 6.2 | 9.07 | 0.55 | 0.01 | 0.01 | 0.02 | 0.13 | 0.06 | 0.22 |
| **125** | *Flacourtia indica* | T | 73 | 2.58 | 5.13 | 0.61 | 0.00 | 0.00 | 0.00 | 0.10 | 0.04 | 0.16 |
| **126** | *Maytenus obscura* | T | 47 | 3.15 | 5.84 | 0.55 | 0.00 | 0.00 | 0.00 | 0.09 | 0.04 | 0.16 |
| **127** | *Landolphia buchananii* | L | 13 | 3.5 |  |  | 0.01 | 0.00 | 0.01 | 0.08 | 0.04 | 0.14 |
| **128** | *Oncinotis tenuiloba* | L | 17 | 2.9 |  |  | 0.00 | 0.00 | 0.01 | 0.07 | 0.03 | 0.11 |
| **129** | *Ricinus communis* | T | 26 | 3.36 | 6.09 | 0.55 | 0.00 | 0.00 | 0.00 | 0.06 | 0.03 | 0.10 |
| **130** | *Maytenus undata* | T | 40 | 2.77 | 5.37 | 0.55 | 0.00 | 0.00 | 0.00 | 0.06 | 0.03 | 0.10 |
| **131** | *Oncoba spinosa* | T | 43 | 2.66 | 5.23 | 0.55 | 0.00 | 0.00 | 0.00 | 0.05 | 0.03 | 0.09 |
| **132** | *Solanecio mannii* | T | 47 | 2.55 | 5.09 | 0.55 | 0.00 | 0.00 | 0.00 | 0.05 | 0.03 | 0.09 |
| **133** | *Solanecio gigas* | T | 43 | 2.63 | 5.19 | 0.55 | 0.00 | 0.00 | 0.00 | 0.05 | 0.02 | 0.09 |
| **134** | *Vernonia amygdalina* | T | 33 | 2.61 | 5.16 | 0.55 | 0.00 | 0.00 | 0.00 | 0.04 | 0.02 | 0.07 |
| **135** | *Stiotocardia beraviensis* | L | 7 | 3.2 |  |  | 0.00 | 0.00 | 0.01 | 0.03 | 0.02 | 0.06 |
| **136** | *Clematis hirsuta* | L | 8 | 2.9 |  |  | 0.00 | 0.00 | 0.01 | 0.03 | 0.01 | 0.05 |
| **137** | *Mikaniopsis clematoides* | L | 8 | 2.8 |  |  | 0.00 | 0.00 | 0.01 | 0.03 | 0.01 | 0.05 |
| **138** | *Turraea holstii* | T | 18 | 2.7 | 5.28 | 0.55 | 0.00 | 0.00 | 0.00 | 0.02 | 0.01 | 0.04 |
| **139** | *Tylophora sylvatica* | L | 7 | 2.76 |  |  | 0.00 | 0.00 | 0.01 | 0.02 | 0.01 | 0.04 |
| **140** | *Balanites aegyptiaca* | T | 14 | 2.75 | 5.34 | 0.55 | 0.00 | 0.00 | 0.00 | 0.02 | 0.01 | 0.03 |
| **141** | *Senna petersiana* | T | 21 | 2.35 | 4.82 | 0.55 | 0.00 | 0.00 | 0.00 | 0.02 | 0.01 | 0.03 |
| **142** | *Smilax anceps* | L | 7 | 2.5 |  |  | 0.00 | 0.00 | 0.00 | 0.02 | 0.01 | 0.03 |
| **143** | Tree species with DBH <5 cm | Sapling | 5958 | 1.1 | 1.40 | ……………. | 0.00 | 0.00 | 0.00 | 0.02 | 0.01 | 0.03 |
| **144** | *Clematis simensis* | L | 6 | 2.6 |  |  | 0.00 | 0.00 | 0.00 | 0.02 | 0.01 | 0.03 |
| **145** | *Dracaena afromontana* | S | 43 | 5.3 | 1.80 | 0.55 | 0.00 | 0.00 | 0.00 | 0.01 | 0.00 | 0.01 |
| **146** | *Acanthus eminens* | S | 39 | 2.88 | 1.45 | 0.55 | 0.00 | 0.00 | 0.00 | 0.00 | 0.00 | 0.01 |
| **147** | *Argomuellera macrophylla* | S | 23 | 2.8 | 2.00 | 0.55 | 0.00 | 0.00 | 0.00 | 0.00 | 0.00 | 0.01 |
| **148** | *Whitfieldia elongata* | S | 15 | 3.55 | 1.75 | 0.55 | 0.00 | 0.00 | 0.00 | 0.00 | 0.00 | 0.00 |
| **149** | *Coffea arabica* | S | 61 | 3.4 | 1.10 | 0.55 | 0.00 | 0.00 | 0.00 | 0.00 | 0.00 | 0.00 |
| **150** | *Erythrococca trichogyne Prain* | S | 22 | 3.8 | 2.30 | 0.55 | 0.00 | 0.00 | 0.00 | 0.00 | 0.00 | 0.00 |
| **151** | *Oxyanthus speciosus* | S | 32 | 2.75 | 1.60 | 0.55 | 0.00 | 0.00 | 0.00 | 0.00 | 0.00 | 0.00 |
| **152** | *Acalypha acrogyna* | S | 13 | 3.1 | 1.80 | 0.55 | 0.00 | 0.00 | 0.00 | 0.00 | 0.00 | 0.00 |
| **153** | *Pavetta abyssinica* | S | 16 | 4.7 | 1.80 | 0.55 | 0.00 | 0.00 | 0.00 | 0.00 | 0.00 | 0.00 |
| **154** | *Vernonia amygdalina* | S | 24 | 3.4 | 1.95 | 0.55 | 0.00 | 0.00 | 0.00 | 0.00 | 0.00 | 0.00 |
| **155** | *Justicia schimperiana* | S | 21 | 4.2 | 1.68 | 0.55 | 0.00 | 0.00 | 0.00 | 0.00 | 0.00 | 0.00 |
| **156** | *Acalypha ornata* | S | 18 | 3.3 | 1.50 | 0.55 | 0.00 | 0.00 | 0.00 | 0.00 | 0.00 | 0.00 |
| **157** | *Grewia mollis* | S | 13 | 4.56 | 2.50 | 0.55 | 0.00 | 0.00 | 0.00 | 0.00 | 0.00 | 0.00 |
| **158** | *Ehertia cymosa* | S | 14 | 3 | 1.25 | 0.55 | 0.00 | 0.00 | 0.00 | 0.00 | 0.00 | 0.00 |
| **159** | *Pavetta oliveriana* | S | 14 | 3.8 | 1.90 | 0.55 | 0.00 | 0.00 | 0.00 | 0.00 | 0.00 | 0.00 |
| **160** | *Calpurina aurea* | S | 12 | 4.2 | 1.90 | 0.55 | 0.00 | 0.00 | 0.00 | 0.00 | 0.00 | 0.00 |
| **161** | *Vangueria madagascariensis* | S | 17 | 3.2 | 1.70 | 0.55 | 0.00 | 0.00 | 0.00 | 0.00 | 0.00 | 0.00 |
| **162** | *Embelia schimperi* | S | 23 | 2.6 | 1.40 | 0.55 | 0.00 | 0.00 | 0.00 | 0.00 | 0.00 | 0.00 |
| **163** | *Psychotria orophila* | S | 13 | 2.6 | 1.60 | 0.55 | 0.00 | 0.00 | 0.00 | 0.00 | 0.00 | 0.00 |
| **164** | *Maytenus gracilipes* | S | 14 | 2.87 | 1.40 | 0.55 | 0.00 | 0.00 | 0.00 | 0.00 | 0.00 | 0.00 |
| **165** | *Bridelia micranta* | S | 9 | 3 | 1.70 | 0.55 | 0.00 | 0.00 | 0.00 | 0.00 | 0.00 | 0.00 |
| **166** | *Bridelia scleroneura* | S | 10 | 3 | 2.10 | 0.55 | 0.00 | 0.00 | 0.00 | 0.00 | 0.00 | 0.00 |
| **167** | *Psidium guajava* | S | 18 | 4.2 | 1.70 | 0.55 | 0.00 | 0.00 | 0.00 | 0.00 | 0.00 | 0.00 |
| **168** | *Maesa lanceolata* | S | 6 | 4.33 | 1.80 | 0.55 | 0.00 | 0.00 | 0.00 | 0.00 | 0.00 | 0.00 |
| **169** | *Clausena anisata* | S | 8 | 4.1 | 1.65 | 0.55 | 0.00 | 0.00 | 0.00 | 0.00 | 0.00 | 0.00 |
| **170** | *Solanecio gigas* | S | 15 | 2.61 | 1.44 | 0.55 | 0.00 | 0.00 | 0.00 | 0.00 | 0.00 | 0.00 |
| **171** | *Vernonia hochstetteri* | S | 19 | 2.7 | 1.26 | 0.55 | 0.00 | 0.00 | 0.00 | 0.00 | 0.00 | 0.00 |
| **172** | *Vernonia auriculifera* | S | 9 | 3.2 | 1.80 | 0.55 | 0.00 | 0.00 | 0.00 | 0.00 | 0.00 | 0.00 |
| **173** | *Rytigynia neglecta* | S | 11 | 3.8 | 1.40 | 0.55 | 0.00 | 0.00 | 0.00 | 0.00 | 0.00 | 0.00 |
| **174** | *Rubus apetalus* | S | 9 | 3.5 | 1.30 | 0.55 | 0.00 | 0.00 | 0.00 | 0.00 | 0.00 | 0.00 |
| **175** | *Vernonia karaguensis* | S | 13 | 2.5 | 1.40 | 0.55 | 0.00 | 0.00 | 0.00 | 0.00 | 0.00 | 0.00 |
| **176** | *Rinorea friisii* | S | 3 | 5.8 | 1.90 | 0.55 | 0.00 | 0.00 | 0.00 | 0.00 | 0.00 | 0.00 |
| **177** | *Rothmannia urcelliformis* | S | 11 | 2.6 | 1.50 | 0.55 | 0.00 | 0.00 | 0.00 | 0.00 | 0.00 | 0.00 |
| **178** | *Vernonia leopoldi* | S | 7 | 2.8 | 1.70 | 0.55 | 0.00 | 0.00 | 0.00 | 0.00 | 0.00 | 0.00 |
| **179** | *Entada abyssinica* | S | 4 | 5.8 | 1.80 | 0.55 | 0.00 | 0.00 | 0.00 | 0.00 | 0.00 | 0.00 |
| **180** | *Myrsine africana* | S | 9 | 2.8 | 1.10 | 0.55 | 0.00 | 0.00 | 0.00 | 0.00 | 0.00 | 0.00 |
| **181** | *Canthium oligocarpum* | S | 8 | 2.7 | 1.60 | 0.55 | 0.00 | 0.00 | 0.00 | 0.00 | 0.00 | 0.00 |
| **182** | *Monothecium glandulosum* | S | 8 | 2.5 | 0.90 | 0.55 | 0.00 | 0.00 | 0.00 | 0.00 | 0.00 | 0.00 |
| **183** | *Maytenus undata* | S | 8 | 2.55 | 1.10 | 0.55 | 0.00 | 0.00 | 0.00 | 0.00 | 0.00 | 0.00 |
| **184** | *Bothriocline schimperi* | S | 11 | 2.4 | 1.40 | 0.55 | 0.00 | 0.00 | 0.00 | 0.00 | 0.00 | 0.00 |
| **185** | *Premna schimperi* | S | 9 | 3.25 | 1.45 | 0.55 | 0.00 | 0.00 | 0.00 | 0.00 | 0.00 | 0.00 |
| **186** | *Maytenus obscura* | S | 8 | 2.6 | 1.60 | 0.55 | 0.00 | 0.00 | 0.00 | 0.00 | 0.00 | 0.00 |
| **187** | *Senna septemtrionali* | S | 6 | 2.6 | 1.55 | 0.55 | 0.00 | 0.00 | 0.00 | 0.00 | 0.00 | 0.00 |
| **188** | *Lippia adoensis* | S | 9 | 3.11 | 0.95 | 0.55 | 0.00 | 0.00 | 0.00 | 0.00 | 0.00 | 0.00 |
| **189** | *Rhus glutinosa* | S | 5 | 3.8 | 1.60 | 0.55 | 0.00 | 0.00 | 0.00 | 0.00 | 0.00 | 0.00 |
| **190** | *Gardenia ternifolia* | S | 5 | 2.3 | 1.50 | 0.55 | 0.00 | 0.00 | 0.00 | 0.00 | 0.00 | 0.00 |
| **191** | *Phyllanthus limmuensis* | S | 6 | 3.9 | 0.90 | 0.55 | 0.00 | 0.00 | 0.00 | 0.00 | 0.00 | 0.00 |
| **192** | *Turraea holstii* | S | 8 | 3.51 | 1.10 | 0.55 | 0.00 | 0.00 | 0.00 | 0.00 | 0.00 | 0.00 |
| **193** | *Solanecio mannii* | S | 6 | 2.62 | 0.70 | 0.55 | 0.00 | 0.00 | 0.00 | 0.00 | 0.00 | 0.00 |
| **194** | *Flacourtia indica* | S | 6 | 2.51 | 0.80 | 0.55 | 0.00 | 0.00 | 0.00 | 0.00 | 0.00 | 0.00 |
| **195** | *Indigofera atriceps* | S | 7 | 4.2 | 0.90 | 0.55 | 0.00 | 0.00 | 0.00 | 0.00 | 0.00 | 0.00 |
| **196** | *Balanites aegyptiaca* | S | 3 | 2.54 | 1.80 | 0.55 | 0.00 | 0.00 | 0.00 | 0.00 | 0.00 | 0.00 |
| **197** | *Oncoba spinosa* | S | 4 | 2.53 | 0.90 | 0.55 | 0.00 | 0.00 | 0.00 | 0.00 | 0.00 | 0.00 |
| **198** | *Phyllanthus ovalifolius* | S | 4 | 4.8 | 1.20 | 0.55 | 0.00 | 0.00 | 0.00 | 0.00 | 0.00 | 0.00 |
| **199** | *Triumfetta rhomboidea* | S | 4 | 3.1 | 0.86 | 0.55 | 0.00 | 0.00 | 0.00 | 0.00 | 0.00 | 0.00 |
| **200** | *Rhamnus prinoides* | S | 5 | 2.8 | 2.10 | 0.55 | 0.00 | 0.00 | 0.00 | 0.00 | 0.00 | 0.00 |
| **201** | *Dombeya torrida* | S | 5 | 3.6 | 1.10 | 0.55 | 0.00 | 0.00 | 0.00 | 0.00 | 0.00 | 0.00 |
| **202** | *Senna petersiana* | S | 4 | 2.55 | 1.30 | 0.55 | 0.00 | 0.00 | 0.00 | 0.00 | 0.00 | 0.00 |
| **203** | *Pentas lanceolata* | S | 4 | 2.74 | 0.90 | 0.55 | 0.00 | 0.00 | 0.00 | 0.00 | 0.00 | 0.00 |
| **204** | *Triumfetta brachyceras* | S | 3 | 2.52 | 0.90 | 0.55 | 0.00 | 0.00 | 0.00 | 0.00 | 0.00 | 0.00 |
| **205** | *Ricinus communis* | S | 4 | 2.53 | 0.90 | 0.55 | 0.00 | 0.00 | 0.00 | 0.00 | 0.00 | 0.00 |
| **206** | *Solanum anguivi* | S | 3 | 2.55 | 0.80 | 0.55 | 0.00 | 0.00 | 0.00 | 0.00 | 0.00 | 0.00 |
|  | **Average** |  | **59.60** | **17.20** | **14.83** | **0.58** | **0.74** | **0.35** | **1.28** | **30.68** | **14.42** | **52.91** |
