# Supplementary material for: Carbon stock quantification and climate mitigation potential of a tropical moist forest in Ethiopia
Source: PLoS One. 2025 Jan 24;20(1):e0316886. doi: 10.1371/journal.pone.0316886 (PMC11760618; doi:10.1371/journal.pone.0316886)
Supplement: S3 Table — (DOC) [file pone.0316886.s009.doc]

**S3 Table**: Wood density determination for all three classes of lying dead woods (LDWs) in Sele-Nono forest. Note that S refers to “Sound” LDW sample, I refers to “Intermediate” LDW sample, and R refers to “Rotten” LDW sample. M is oven-dry mass (gm), and V is saturated/fresh volume (cm^3^).

| **Plot No.** | **Wood density determination of “Sound” LDWs** | | | | **Wood density determination of “Intermediate” LDWs** | | | | **Wood density determination of “Rotten” LDWs** | | | |
| --- | --- | --- | --- | --- | --- | --- | --- | --- | --- | --- | --- | --- |
|  | Code | M | V | Density | Code | M | V | Density | Code | M | V | Density |
| **1** | S1 | 171.5 | 140 | 1.225 | I1 | 122.7 | 220 | 0.557727 | R1 | 22 | 350 | 0.062857 |
| **2** | S2 | 38 | 65 | 0.584615 | I2 | 56 | 125 | 0.448 | R2 | 18.4 | 130 | 0.141538 |
| **3** | S3 | 37 | 60 | 0.616667 | I3 | 47 | 90 | 0.522222 | R3 | 10.2 | 50 | 0.204 |
| **4** | S4 | 0 |  | 0 | I4 | 0 |  | 0 | R4 | 18.8 | 90 | 0.208889 |
| **5** | S5 | 101.5 | 180 | 0.563889 | I5 | 75.5 | 150 | 0.503333 | R5 | 22.3 | 85 | 0.262353 |
| **6** | S6 | 0 |  | 0 | I6 | 0 |  | 0 | R6 | 11.6 | 45 | 0.257778 |
| **7** | S7 | 0 |  | 0 | I7 | 0 |  | 0 | R7 | 25.9 | 75 | 0.345333 |
| **8** | S8 | 70 | 65 | 1.076923 | I8 | 58 | 100 | 0.58 | R8 | 19.4 | 65 | 0.298462 |
| **9** | S9 | 32.5 | 45 | 0.722222 | I9 | 60 | 100 | 0.6 | R9 | 28 | 115 | 0.243478 |
| **10** | S10 | 29.8 | 45 | 0.662222 | I10 | 68.5 | 150 | 0.456667 | R10 | 12.5 | 50 | 0.25 |
| **11** | S11 | 59.7 | 75 | 0.796 | I11 | 43.6 | 75 | 0.581333 | R11 | 19 | 65 | 0.292308 |
| **12** | S12 | 29 | 25 | 1.16 | I12 | 47 | 150 | 0.313333 | R12 | 12.5 | 60 | 0.208333 |
| **13** | S13 | 16.8 | 25 | 0.672 | I13 | 40 | 95 | 0.421053 | R13 | 8.2 | 30 | 0.273333 |
| **14** | S14 | 40 | 75 | 0.533333 | I14 | 39.3 | 175 | 0.224571 | R14 | 4.9 | 20 | 0.245 |
| **15** | S15 | 0 |  | 0 | I15 | 0 |  | 0 | R15 | 22 | 65 | 0.338462 |
| **16** | S16 | 229 | 260 | 0.880769 | I16 | 52 | 150 | 0.346667 | R16 | 16.8 | 60 | 0.28 |
| **17** | S17 | 46 | 50 | 0.92 | I17 | 40.7 | 100 | 0.407 | R17 | 23 | 45 | 0.511111 |
| **18** | S18 | 17.8 | 15 | 1.186667 | I18 | 67 | 120 | 0.558333 | R18 | 17.3 | 45 | 0.384444 |
| **19** | S19 | 29.5 | 15 | 1.966667 | I19 | 38 | 100 | 0.38 | R19 | 16.5 | 40 | 0.4125 |
| **20** | S20 | 24 | 50 | 0.48 | I20 | 15 | 45 | 0.333333 | R20 | 27 | 70 | 0.385714 |
| **21** | S21 | 20 | 50 | 0.4 | I21 | 40 | 125 | 0.32 | R21 | 15 | 45 | 0.333333 |
| **22** | S22 | 23 | 25 | 0.92 | I22 | 33.5 | 125 | 0.268 | R22 | 5 | 65 | 0.076923 |
| **23** | S23 | 39 | 50 | 0.78 | I23 | 26.5 | 140 | 0.189286 | R23 | 7.5 | 40 | 0.1875 |
| **24** | S24 | 92.5 | 150 | 0.616667 | I24 | 37.5 | 125 | 0.3 | R24 | 3.5 | 30 | 0.116667 |
| **25** | S25 | 81.5 | 150 | 0.543333 | I25 | 19 | 100 | 0.19 | R25 | 20.6 | 80 | 0.2575 |
| **26** | S26 | 6 | 10 | 0.6 | I26 | 20 | 50 | 0.4 | R26 | 15.9 | 60 | 0.265 |
| **27** | S27 | 113 | 200 | 0.565 | I27 | 28.5 | 125 | 0.228 | R27 | 15.1 | 60 | 0.251667 |
| **28** | S28 | 32 | 150 | 0.213333 | I28 | 15.5 | 50 | 0.31 | R28 | 2.4 | 35 | 0.068571 |
| **29** | S29 | 90.5 | 190 | 0.476316 | I29 | 36.3 | 120 | 0.3025 | R29 | 20.8 | 150 | 0.138667 |
| **30** | S30 | 67 | 100 | 0.67 | I30 | 103.5 | 150 | 0.69 | R30 | 25.4 | 160 | 0.15875 |
| **31** | S31 | 60.5 | 90 | 0.672222 | I31 | 35.5 | 85 | 0.417647 | R31 | 25 | 75 | 0.333333 |
| **32** | S32 | 58.7 | 150 | 0.391333 | I32 | 29.5 | 110 | 0.268182 | R32 | 15.4 | 80 | 0.1925 |
| **33** | S33 | 109.2 | 210 | 0.52 | I33 | 42 | 90 | 0.466667 | R33 | 16.2 | 50 | 0.324 |
| **34** | S34 | 37 | 80 | 0.4625 | I34 | 22 | 50 | 0.44 | R34 | 23 | 65 | 0.353846 |
| **35** | S35 | 39 | 85 | 0.458824 | I35 | 15 | 50 | 0.3 | R35 | 10.6 | 30 | 0.353333 |
| **36** | S36 | 49 | 90 | 0.544444 | I36 | 53 | 125 | 0.424 | R36 | 24 | 60 | 0.4 |
| **37** | S37 | 91 | 140 | 0.65 | I37 | 31 | 75 | 0.413333 | R37 | 23 | 80 | 0.2875 |
| **38** | S38 | 171.5 | 225 | 0.762222 | I38 | 20 | 40 | 0.5 | R38 | 15.1 | 80 | 0.18875 |
| **39** | S39 | 42.8 | 60 | 0.713333 | I39 | 11.5 | 30 | 0.383333 | R39 | 10.5 | 35 | 0.3 |
| **40** | S40 | 0 |  | 0 | I40 | 0 |  | 0 | R40 | 21 | 100 | 0.21 |
| **41** | S41 | 35.7 | 80 | 0.44625 | I41 | 20.5 | 50 | 0.41 | R41 | 17 | 60 | 0.283333 |
| **42** | S42 | 99.7 | 250 | 0.3988 | I42 | 9 | 10 | 0.9 | R42 | 25.7 | 95 | 0.270526 |
| **43** | S43 | 0 |  | 0 | I43 | 0 |  | 0 | R43 | 10.4 | 40 | 0.26 |
| **44** | S44 | 160.9 | 210 | 0.76619 | I44 | 64.7 | 140 | 0.462143 | R44 | 12.3 | 55 | 0.223636 |
| **45** | S45 | 68.7 | 125 | 0.5496 | I45 | 52 | 135 | 0.385185 | R45 | 12 | 50 | 0.24 |
| **46** | S46 | 29.5 | 70 | 0.421429 | I46 | 70.6 | 155 | 0.455484 | R46 | 18 | 65 | 0.276923 |
| **47** | S47 | 39.3 | 60 | 0.655 | I47 | 71 | 110 | 0.645455 | R47 | 15.2 | 80 | 0.19 |
| **48** | S48 | 69.5 | 110 | 0.631818 | I48 | 76.9 | 95 | 0.809474 | R48 | 20.3 | 110 | 0.184545 |
| **49** | S49 | 32.1 | 65 | 0.493846 | I49 | 52 | 80 | 0.65 | R49 | 5.6 | 25 | 0.224 |
| **50** | S50 | 0 |  | 0 | I50 | 0 | 0 | 0 | R50 | 18.4 | 60 | 0.306667 |
| **51** | S51 | 45.8 | 85 | 0.538824 | I51 | 80.6 | 130 | 0.62 | R51 | 32 | 230 | 0.13913 |
| **52** | S52 | 65.7 | 120 | 0.5475 | I52 | 72 | 120 | 0.6 | R52 | 5 | 25 | 0.2 |
| **53** | S53 | 218 | 410 | 0.531707 | I53 | 53 | 85 | 0.623529 | R53 | 25.7 | 100 | 0.257 |
| **54** | S54 | 50 | 125 | 0.4 | I54 | 86 | 140 | 0.614286 | R54 | 19.5 | 65 | 0.3 |
| **55** | S55 | 15.6 | 35 | 0.445714 | I55 | 37 | 105 | 0.352381 | R55 | 11.5 | 50 | 0.23 |
| **56** | S56 | 27.4 | 55 | 0.498182 | I56 | 44 | 155 | 0.283871 | R56 | 24 | 90 | 0.266667 |
| **57** | S57 | 23.4 | 35 | 0.668571 | I57 | 78 | 125 | 0.624 | R57 | 30 | 250 | 0.12 |
| **58** | S58 | 18 | 45 | 0.4 | I58 | 64 | 165 | 0.387879 | R58 | 21.5 | 65 | 0.330769 |
| **59** | S59 | 21.3 | 25 | 0.852 | I59 | 53 | 110 | 0.481818 | R59 | 20.5 | 70 | 0.292857 |
| **60** | S60 | 39 | 90 | 0.433333 | I60 | 48 | 80 | 0.6 | R60 | 14.5 | 60 | 0.241667 |
| **61** | S61 | 92.5 | 250 | 0.37 | I61 | 20.5 | 75 | 0.273333 | R61 | 36.5 | 260 | 0.140385 |
| **62** | S62 | 85.5 | 140 | 0.610714 | I62 | 8 | 25 | 0.32 | R62 | 24 | 60 | 0.4 |
| **63** | S63 | 24 | 55 | 0.436364 | I63 | 57 | 115 | 0.495652 | R63 | 26 | 70 | 0.371429 |
| **64** | S64 | 109 | 365 | 0.29863 | I64 | 28.3 | 95 | 0.297895 | R64 | 33 | 150 | 0.22 |
| **65** | S65 | 53.6 | 145 | 0.369655 | I65 | 15.7 | 105 | 0.149524 | R65 | 38 | 205 | 0.185366 |
| **66** | S66 | 93.4 | 225 | 0.415111 | I66 | 33 | 85 | 0.388235 | R66 | 19 | 45 | 0.422222 |
| **67** | S67 | 64 | 120 | 0.533333 | I67 | 16.7 | 80 | 0.20875 | R67 | 26 | 50 | 0.52 |
| **68** | S68 | 58.7 | 95 | 0.617895 | I68 | 24 | 75 | 0.32 | R68 | 25 | 85 | 0.294118 |
| **69** | S69 | 0 |  | 0 | I69 | 0 |  | 0 | R69 | 14 | 60 | 0.233333 |
| **70** | S70 | 106.5 | 350 | 0.304286 | I70 | 20.5 | 80 | 0.25625 | R70 | 20.5 | 70 | 0.292857 |
| **71** | S71 | 34.8 | 80 | 0.435 | I71 | 43 | 160 | 0.26875 | R71 | 14.5 | 40 | 0.3625 |
| **72** | S72 | 34.9 | 85 | 0.410588 | I72 | 130 | 270 | 0.481481 | R72 | 36.5 | 210 | 0.17381 |
| **73** | S73 | 45 | 90 | 0.5 | I73 | 30 | 80 | 0.375 | R73 | 21 | 45 | 0.466667 |
| **74** | S74 | 88 | 110 | 0.8 | I74 | 32 | 75 | 0.426667 | R74 | 17 | 40 | 0.425 |
| **75** | S75 | 172 | 340 | 0.505882 | I75 | 39 | 85 | 0.458824 | R75 | 13 | 40 | 0.325 |
| **76** | S76 | 74.7 | 70.5 | 1.059574 | I76 | 20 | 65 | 0.307692 | R76 | 22 | 90 | 0.244444 |
| **77** | S77 | 32.5 | 74.5 | 0.436242 | I77 | 18 | 60 | 0.3 | R77 | 15 | 55 | 0.272727 |
| **78** | S78 | 28.8 | 55.5 | 0.518919 | I78 | 47 | 115 | 0.408696 | R78 | 9 | 40 | 0.225 |
| **79** | S79 | 55.4 | 80.5 | 0.688199 | I79 | 34 | 70 | 0.485714 | R79 | 12 | 65 | 0.184615 |
| **80** | S80 | 34 | 55 | 0.618182 | I80 | 15 | 55 | 0.272727 | R80 | 25 | 150 | 0.166667 |
| **81** | S81 | 17 | 60 | 0.283333 | I81 | 11 | 35 | 0.314286 | R81 | 22 | 40 | 0.55 |
| **82** | S82 | 40 | 60 | 0.666667 | I82 | 28.4 | 65 | 0.436923 | R82 | 20.6 | 75 | 0.274667 |
| **83** | S83 | 66 | 110 | 0.6 | I83 | 23.2 | 60 | 0.386667 | R83 | 11.5 | 75 | 0.153333 |
| **84** | S84 | 225 | 300 | 0.75 | I84 | 5 | 20 | 0.25 | R84 | 2 | 10 | 0.2 |
| **85** | S85 | 44.7 | 70 | 0.638571 | I85 | 130.4 | 280 | 0.465714 | R85 | 12.5 | 85 | 0.147059 |
| **86** | S86 | 16.5 | 40 | 0.4125 | I86 | 52.5 | 235 | 0.223404 | R86 | 21.5 | 65 | 0.330769 |
| **87** | S87 | 30.4 | 30 | 1.013333 | I87 | 50 | 120 | 0.416667 | R87 | 20.5 | 60 | 0.341667 |
| **88** | S88 | 23.4 | 50 | 0.468 | I88 | 65 | 155 | 0.419355 | R88 | 14.5 | 60 | 0.241667 |
| **89** | S89 | 23 | 48 | 0.479167 | I89 | 78 | 140 | 0.557143 | R89 | 11 | 70 | 0.157143 |
| **90** | S90 | 87 | 110 | 0.790909 | I90 | 64 | 110 | 0.581818 | R90 | 21 | 130 | 0.161538 |
| **Average** |  |  |  | **0.563515** |  |  |  | **0.383302** |  |  |  | **0.263616** |
