# Supplementary material for: Carbon stock quantification and climate mitigation potential of a tropical moist forest in Ethiopia
Source: PLoS One. 2025 Jan 24;20(1):e0316886. doi: 10.1371/journal.pone.0316886 (PMC11760618; doi:10.1371/journal.pone.0316886)
Supplement: S4 Table — (DOC) [file pone.0316886.s010.doc]

**S4 Table**: Total biomass, carbon stock, and soil organic carbon of Sele-Nono forest at plot level (t/ha)

| **Plot No.** | **Strata No.** | **AGB** | **AGC** | **BGB** | **BGC** | **LB** | **LC** | **HB** | **HC** | **LDWB** | **LDWC** | **SOC** | **TB** | **TC** |
| --- | --- | --- | --- | --- | --- | --- | --- | --- | --- | --- | --- | --- | --- | --- |
| **1** | 4 | 309.4 | 145.42 | 61.882 | 29.084 | 8.82 | 4.1454 | 2.78 | 1.3066 | 30.14 | 14.1658 | 227.6 | 413.032 | 421.72 |
| **2** | 5 | 87.27 | 41.016 | 17.454 | 8.2033 | 2.56 | 1.2032 | 1.05 | 0.4935 | 9.67 | 4.5449 | 66.58 | 118.004 | 122.04 |
| **3** | 4 | 201.4 | 94.686 | 40.292 | 18.937 | 6.19 | 2.9093 | 2.58 | 1.2126 | 18.84 | 8.8548 | 247.89 | 269.362 | 374.49 |
| **4** | 1 | 118.4 | 55.685 | 23.696 | 11.137 | 2.97 | 1.3959 | 4.05 | 1.9035 | 0 | 0 | 64.4 | 149.196 | 134.52 |
| **5** | 1 | 360.8 | 169.58 | 72.164 | 33.917 | 12.86 | 6.0442 | 1.96 | 0.9212 | 38.63 | 18.1561 | 174.02 | 486.434 | 402.64 |
| **6** | 4 | 40.07 | 18.832 | 8.014 | 3.7665 | 8.51 | 3.9997 | 4.51 | 2.1197 | 0 | 0 | 246.82 | 61.104 | 275.53 |
| **7** | 1 | 98 | 46.06 | 19.6 | 9.212 | 2.01 | 0.9447 | 5.88 | 2.7636 | 0 | 0 | 52.11 | 125.49 | 111.09 |
| **8** | 3 | 268.6 | 126.27 | 53.736 | 25.255 | 7.04 | 3.3088 | 1.67 | 0.7849 | 31.96 | 15.0212 | 262.04 | 363.086 | 432.69 |
| **9** | 2 | 360.6 | 169.49 | 72.124 | 33.898 | 14.93 | 7.0171 | 2.42 | 1.1374 | 45.03 | 21.1641 | 184.6 | 495.124 | 417.30 |
| **10** | 3 | 43.24 | 20.322 | 8.648 | 4.0645 | 9.58 | 4.5026 | 4.16 | 1.9552 | 0.5 | 0.235 | 208.17 | 66.128 | 239.25 |
| **11** | 3 | 120.5 | 56.644 | 24.104 | 11.328 | 5.16 | 2.4252 | 3.67 | 1.7249 | 14.83 | 6.9701 | 122.62 | 168.284 | 201.71 |
| **12** | 3 | 37.48 | 17.615 | 7.496 | 3.5231 | 7.83 | 3.6801 | 5.08 | 2.3876 | 1.46 | 0.6862 | 227.36 | 59.346 | 255.25 |
| **13** | 1 | 87.53 | 41.139 | 17.506 | 8.2278 | 1.42 | 0.6674 | 3.91 | 1.8377 | 1.2 | 0.564 | 40.47 | 111.566 | 92.906 |
| **14** | 4 | 185.8 | 87.326 | 37.16 | 17.465 | 14.83 | 6.9701 | 3.16 | 1.4852 | 23.84 | 11.2048 | 308.38 | 264.79 | 432.83 |
| **15** | 3 | 45.28 | 21.281 | 9.056 | 4.2563 | 11.74 | 5.5178 | 5.13 | 2.4111 | 0 | 0 | 284.68 | 71.206 | 318.14 |
| **16** | 4 | 45.14 | 21.215 | 9.028 | 4.2431 | 7.69 | 3.6143 | 3.74 | 1.7578 | 2.24 | 1.0528 | 220.53 | 67.838 | 252.41 |
| **17** | 5 | 110.8 | 52.113 | 22.176 | 10.427 | 6.11 | 2.8717 | 1.74 | 0.8178 | 11.46 | 5.3862 | 89.42 | 152.366 | 161.03 |
| **18** | 4 | 188.2 | 88.477 | 37.65 | 17.695 | 16.28 | 7.6516 | 3.07 | 1.4429 | 27.93 | 13.1271 | 356.49 | 273.18 | 484.88 |
| **19** | 3 | 253.9 | 119.35 | 50.788 | 23.870 | 8.93 | 4.1971 | 2.78 | 1.3066 | 37.91 | 17.8177 | 142.35 | 354.348 | 308.89 |
| **20** | 5 | 90.52 | 42.544 | 18.104 | 8.5088 | 4.37 | 2.0539 | 1.86 | 0.8742 | 6.28 | 2.9516 | 74.82 | 121.134 | 131.75 |
| **21** | 3 | 302.6 | 142.22 | 60.52 | 28.444 | 10.83 | 5.0901 | 2.86 | 1.3442 | 38.48 | 18.0856 | 147.65 | 415.29 | 342.83 |
| **22** | 5 | 115.6 | 54.369 | 23.136 | 10.873 | 5.24 | 2.4628 | 1.66 | 0.7802 | 10.84 | 5.0948 | 86.37 | 156.556 | 159.95 |
| **23** | 3 | 200.2 | 94.131 | 40.056 | 18.826 | 8.66 | 4.0702 | 2.31 | 1.0857 | 40.62 | 19.0914 | 275.52 | 291.926 | 412.72 |
| **24** | 2 | 307.3 | 144.46 | 61.474 | 28.892 | 10.93 | 5.1371 | 3.04 | 1.4288 | 26.47 | 12.4409 | 152.13 | 409.284 | 344.49 |
| **25** | 4 | 230.8 | 108.50 | 46.174 | 21.701 | 9.06 | 4.2582 | 1.04 | 0.4888 | 34.62 | 16.2714 | 232.04 | 321.764 | 383.26 |
| **26** | 5 | 120.6 | 56.705 | 24.13 | 11.341 | 3.18 | 1.4946 | 1.64 | 0.7708 | 5.11 | 2.4017 | 61.04 | 154.71 | 133.75 |
| **27** | 6 | 112.4 | 52.860 | 22.494 | 10.572 | 3.74 | 1.7578 | 2.69 | 1.2643 | 4.67 | 2.1949 | 58.54 | 146.064 | 127.19 |
| **28** | 4 | 200.1 | 94.079 | 40.034 | 18.815 | 11.53 | 5.4191 | 3.27 | 1.5369 | 36.88 | 17.3336 | 224.71 | 291.884 | 361.89 |
| **29** | 3 | 340.6 | 160.12 | 68.138 | 32.024 | 9.96 | 4.6812 | 3.66 | 1.7202 | 30.52 | 14.3444 | 149.53 | 452.968 | 362.42 |
| **30** | 5 | 113.6 | 53.401 | 22.724 | 10.680 | 2.42 | 1.1374 | 1.25 | 0.5875 | 16.89 | 7.9383 | 64.61 | 156.904 | 138.35 |
| **31** | 1 | 103.6 | 48.696 | 20.722 | 9.7393 | 4.72 | 2.2184 | 2.81 | 1.3207 | 0.78 | 0.3666 | 36.38 | 132.642 | 98.721 |
| **32** | 3 | 287.1 | 134.97 | 57.438 | 26.995 | 7.94 | 3.7318 | 2.68 | 1.2596 | 28.66 | 13.4702 | 132.99 | 383.908 | 313.42 |
| **33** | 3 | 157.6 | 74.109 | 31.536 | 14.821 | 3.98 | 1.8706 | 3.13 | 1.4711 | 14.93 | 7.0171 | 105.84 | 211.256 | 205.13 |
| **34** | 3 | 263.7 | 123.94 | 52.742 | 24.788 | 11.74 | 5.5178 | 4.71 | 2.2137 | 24.22 | 11.3834 | 116.52 | 357.122 | 284.36 |
| **35** | 5 | 157.6 | 74.109 | 31.536 | 14.821 | 12.39 | 5.8233 | 3.94 | 1.8518 | 26.04 | 12.2388 | 284.77 | 231.586 | 393.61 |
| **36** | 4 | 240.3 | 112.98 | 48.078 | 22.596 | 14.93 | 7.0171 | 2.11 | 0.9917 | 44.83 | 21.0701 | 267.8 | 350.338 | 432.45 |
| **37** | 2 | 248.7 | 116.90 | 49.746 | 23.380 | 11.04 | 5.1888 | 3.11 | 1.4617 | 32.74 | 15.3878 | 158.84 | 345.366 | 321.16 |
| **38** | 4 | 136.5 | 64.164 | 27.304 | 12.832 | 10.63 | 4.9961 | 1.03 | 0.4841 | 38.62 | 18.1514 | 238.38 | 214.104 | 339.00 |
| **39** | 4 | 168.9 | 79.401 | 33.788 | 15.880 | 13.82 | 6.4954 | 3.21 | 1.5087 | 34.86 | 16.3842 | 296.16 | 254.618 | 415.83 |
| **40** | 1 | 82.94 | 38.981 | 16.588 | 7.7963 | 1.47 | 0.6909 | 4.18 | 1.9646 | 0 | 0 | 41.56 | 105.178 | 90.993 |
| **41** | 2 | 166.9 | 78.443 | 33.38 | 15.688 | 6.64 | 3.1208 | 1.92 | 0.9024 | 32.54 | 15.2938 | 218.84 | 241.38 | 332.28 |
| **42** | 4 | 233.9 | 109.94 | 46.786 | 21.989 | 7.87 | 3.6989 | 2.17 | 1.0199 | 26.67 | 12.5349 | 252.31 | 317.426 | 401.50 |
| **43** | 3 | 37.25 | 17.507 | 7.45 | 3.5015 | 6.99 | 3.2853 | 4.18 | 1.9646 | 0 | 0 | 231.33 | 55.87 | 257.58 |
| **44** | 4 | 130.6 | 61.405 | 26.13 | 12.281 | 9.73 | 4.5731 | 3.75 | 1.7625 | 8.68 | 4.0796 | 111.48 | 178.94 | 195.58 |
| **45** | 3 | 210.4 | 98.916 | 42.092 | 19.783 | 9.27 | 4.3569 | 3.94 | 1.8518 | 26.49 | 12.4503 | 128.16 | 292.252 | 265.51 |
| **46** | 3 | 330.2 | 155.23 | 66.056 | 31.046 | 10.64 | 5.0008 | 2.53 | 1.1891 | 28.52 | 13.4044 | 126.53 | 438.026 | 332.40 |
| **47** | 1 | 101.4 | 47.695 | 20.296 | 9.5391 | 3.11 | 1.4617 | 3.36 | 1.5792 | 1.23 | 0.5781 | 52.34 | 129.476 | 113.19 |
| **48** | 4 | 255.7 | 120.19 | 51.148 | 24.039 | 11.75 | 5.5225 | 2.83 | 1.3301 | 30.8 | 14.476 | 283.55 | 352.268 | 449.11 |
| **49** | 1 | 378.4 | 177.89 | 75.698 | 35.578 | 7.48 | 3.5156 | 3.75 | 1.7625 | 25.17 | 11.8299 | 108.69 | 490.588 | 339.26 |
| **50** | 3 | 30.6 | 14.382 | 6.12 | 2.8764 | 5.24 | 2.4628 | 3.16 | 1.4852 | 0 | 0 | 210.26 | 45.12 | 231.46 |
| **51** | 2 | 267.3 | 125.64 | 53.464 | 25.128 | 9.88 | 4.6436 | 2.66 | 1.2502 | 36.92 | 17.3524 | 164.81 | 370.244 | 338.82 |
| **52** | 4 | 120.7 | 56.761 | 24.154 | 11.352 | 9.31 | 4.3757 | 4.87 | 2.2889 | 29.74 | 13.9778 | 255.34 | 188.844 | 344.09 |
| **53** | 5 | 90.45 | 42.511 | 18.09 | 8.5023 | 4.26 | 2.0022 | 1.93 | 0.9071 | 24.48 | 11.5056 | 73.59 | 139.21 | 139.01 |
| **54** | 2 | 240.8 | 113.17 | 48.16 | 22.635 | 9.99 | 4.6953 | 3.89 | 1.8283 | 32.35 | 15.2045 | 182.05 | 335.19 | 339.58 |
| **55** | 2 | 140.8 | 66.213 | 28.176 | 13.242 | 4.33 | 2.0351 | 3.07 | 1.4429 | 12.64 | 5.9408 | 93.71 | 189.096 | 182.58 |
| **56** | 3 | 210.3 | 98.869 | 42.072 | 19.773 | 10.63 | 4.9961 | 2.64 | 1.2408 | 29.53 | 13.8791 | 166.62 | 295.232 | 305.37 |
| **57** | 2 | 318.3 | 149.63 | 63.676 | 29.927 | 12.84 | 6.0348 | 2.85 | 1.3395 | 31.36 | 14.7392 | 159.43 | 429.106 | 361.10 |
| **58** | 1 | 99.36 | 46.699 | 19.872 | 9.3398 | 2.78 | 1.3066 | 2.88 | 1.3536 | 1.53 | 0.7191 | 50.06 | 126.422 | 109.47 |
| **59** | 5 | 89.35 | 41.994 | 17.87 | 8.3989 | 6.33 | 2.9751 | 2.74 | 1.2878 | 8.98 | 4.2206 | 75.8 | 125.27 | 134.67 |
| **60** | 2 | 288.5 | 135.60 | 57.704 | 27.120 | 10.85 | 5.0995 | 3.01 | 1.4147 | 33.6 | 15.792 | 121.59 | 393.684 | 306.62 |
| **61** | 2 | 286.7 | 134.76 | 57.346 | 26.952 | 13.68 | 6.4296 | 3.11 | 1.4617 | 28.28 | 13.2916 | 118.5 | 389.146 | 301.39 |
| **62** | 2 | 170.3 | 80.055 | 34.066 | 16.011 | 11.95 | 5.6165 | 2.63 | 1.2361 | 28.89 | 13.5783 | 109.47 | 247.866 | 225.96 |
| **63** | 3 | 270.3 | 127.04 | 54.062 | 25.409 | 5.84 | 2.7448 | 2.55 | 1.1985 | 24.36 | 11.4492 | 204.87 | 357.122 | 372.71 |
| **64** | 3 | 382.7 | 179.88 | 76.546 | 35.976 | 14.6 | 6.862 | 2.9 | 1.363 | 33.7 | 15.839 | 153.05 | 510.476 | 392.97 |
| **65** | 5 | 96.36 | 45.289 | 19.272 | 9.0578 | 2.48 | 1.1656 | 1.03 | 0.4841 | 6.64 | 3.1208 | 48.02 | 125.782 | 107.13 |
| **66** | 2 | 265.4 | 124.78 | 53.098 | 24.956 | 8.92 | 4.1924 | 2.04 | 0.9588 | 28.74 | 13.5078 | 142.86 | 358.288 | 311.25 |
| **67** | 4 | 236.6 | 111.23 | 47.332 | 22.246 | 8.45 | 3.9715 | 2.63 | 1.2361 | 27.75 | 13.0425 | 258.18 | 322.822 | 409.90 |
| **68** | 2 | 288.3 | 135.53 | 57.676 | 27.107 | 9.42 | 4.4274 | 2.46 | 1.1562 | 29.14 | 13.6958 | 174.93 | 387.076 | 356.85 |
| **69** | 1 | 87.62 | 41.181 | 17.524 | 8.2362 | 0.88 | 0.4136 | 5.37 | 2.5239 | 0 | 0 | 42.73 | 111.394 | 95.085 |
| **70** | 3 | 285.7 | 134.31 | 57.156 | 26.863 | 6.98 | 3.2806 | 2.74 | 1.2878 | 22.63 | 10.6361 | 168.93 | 375.286 | 345.31 |
| **71** | 3 | 170.4 | 80.102 | 34.086 | 16.020 | 8.92 | 4.1924 | 2.08 | 0.9776 | 28.7 | 13.489 | 185.93 | 244.216 | 300.71 |
| **72** | 2 | 267.8 | 125.90 | 53.578 | 25.181 | 12.04 | 5.6588 | 2.99 | 1.4053 | 28.98 | 13.6206 | 178.6 | 365.478 | 350.37 |
| **73** | 5 | 169.3 | 79.594 | 33.87 | 15.918 | 12.43 | 5.8421 | 3.04 | 1.4288 | 31.46 | 14.7862 | 263.53 | 250.15 | 381.10 |
| **74** | 4 | 208.6 | 98.070 | 41.732 | 19.614 | 7.16 | 3.3652 | 2.15 | 1.0105 | 34.08 | 16.0176 | 251.83 | 293.782 | 389.90 |
| **75** | 5 | 116.6 | 54.802 | 23.32 | 10.960 | 4.24 | 1.9928 | 1.92 | 0.9024 | 12.42 | 5.8374 | 62.71 | 158.5 | 137.20 |
| **76** | 4 | 268.4 | 126.10 | 53.694 | 25.236 | 8.63 | 4.0561 | 2.07 | 0.9729 | 38.78 | 18.2266 | 194.6 | 371.644 | 369.27 |
| **77** | 5 | 104.5 | 49.143 | 20.912 | 9.8286 | 4.29 | 2.0163 | 2.17 | 1.0199 | 8.64 | 4.0608 | 65.93 | 140.572 | 131.99 |
| **78** | 2 | 260.8 | 122.61 | 52.178 | 24.523 | 11.85 | 5.5695 | 2.62 | 1.2314 | 27.94 | 13.1318 | 164.02 | 355.478 | 331.09 |
| **79** | 2 | 266.9 | 125.45 | 53.386 | 25.091 | 10.63 | 4.9961 | 3.86 | 1.8142 | 26.15 | 12.2905 | 145.51 | 360.956 | 315.15 |
| **80** | 2 | 235.1 | 110.53 | 47.036 | 22.106 | 7.44 | 3.4968 | 2.34 | 1.0998 | 28.42 | 13.3574 | 242.94 | 320.416 | 393.53 |
| **81** | 4 | 242.7 | 114.08 | 48.548 | 22.817 | 9.56 | 4.4932 | 2.11 | 0.9917 | 31.88 | 14.9836 | 263.04 | 334.838 | 420.41 |
| **82** | 2 | 230.6 | 108.38 | 46.122 | 21.677 | 13.77 | 6.4719 | 2.03 | 0.9541 | 34.35 | 16.1445 | 155.37 | 326.882 | 309.00 |
| **83** | 3 | 48.83 | 22.950 | 9.766 | 4.5900 | 14.63 | 6.8761 | 5.64 | 2.6508 | 1.26 | 0.5922 | 242.55 | 80.126 | 280.20 |
| **84** | 2 | 232.8 | 109.45 | 46.578 | 21.891 | 9.58 | 4.5026 | 2.64 | 1.2408 | 31.41 | 14.7627 | 179.51 | 323.098 | 331.36 |
| **85** | 2 | 125.4 | 58.970 | 25.094 | 11.794 | 8.01 | 3.7647 | 4.11 | 1.9317 | 11.6 | 5.452 | 135.14 | 174.284 | 217.05 |
| **86** | 2 | 254.8 | 119.76 | 50.964 | 23.953 | 11.84 | 5.5648 | 2.17 | 1.0199 | 28.58 | 13.4326 | 123.73 | 348.374 | 287.46 |
| **87** | 4 | 175.5 | 82.503 | 35.108 | 16.500 | 15.38 | 7.2286 | 2.94 | 1.3818 | 32.85 | 15.4395 | 275.48 | 261.818 | 398.53 |
| **88** | 2 | 250.8 | 117.89 | 50.166 | 23.57802 | 10.85 | 5.0995 | 2.53 | 1.1891 | 28.12 | 13.2164 | 134.01 | 342.496 | 294.98 |
| **89** | 1 | 240.8 | 113.19 | 48.166 | 22.63802 | 6.86 | 3.2242 | 2.13 | 1.0011 | 2.03 | 0.9541 | 72.88 | 300.016 | 213.88 |
| **90** | 5 | 98.67 | 46.374 | 19.734 | 9.27498 | 5.85 | 2.7495 | 2.63 | 1.2361 | 10.85 | 5.0995 | 84.37 | 137.734 | 149.10 |
| **Average** |  | 189.1 | 88.92 | 37.83 | 17.7845 | 8.501 | 4.0111 | 2.925 | 1.374 | 21.36 | 10.0400 | 162.6938 | 259.825 | 284.81 |
| **sd** |  | ±91.62 | ±43.06 | ±18.32 | ±8.61 | ±3.76 | ±1.76 | ±1.03 | ±0.48 | ±13.52 | ±6.35 | ±78.73 | ±122.59 | ±107.81 |
