# Supplementary material for: Carbon stock quantification and climate mitigation potential of a tropical moist forest in Ethiopia
Source: PLoS One. 2025 Jan 24;20(1):e0316886. doi: 10.1371/journal.pone.0316886 (PMC11760618; doi:10.1371/journal.pone.0316886)
Supplement: S5 Table — (DOC) [file pone.0316886.s011.doc]

S5 Table: Bulk density calculation and soil organic carbon storage in Sele-Nono forest at each sample plots

| **Plot No.** | **Depth (cm)** | **V (cm3)** | **Mass of oven-dry bulk soil**  **(gm)** | **C content of SOC of the composite soil**  **(%)** | **Bulk density**  **(gcm-3)** | **SOC (t/ha)** |
| --- | --- | --- | --- | --- | --- | --- |
| **1** | 30 | 98 | 115.90 | 6.41 | 1.18 | 227.60 |
| **2** | 30 | 98 | 51.41 | 4.22 | 0.52 | 66.58 |
| **3** | 30 | 98 | 148.03 | 5.46 | 1.51 | 247.89 |
| **4** | 30 | 98 | 61.341 | 3.42 | 0.62 | 64.40 |
| **5** | 30 | 98 | 158.36 | 3.58 | 1.61 | 174.02 |
| **6** | 30 | 98 | 89.22 | 9.03 | 0.91 | 246.82 |
| **7** | 30 | 98 | 39.68 | 4.28 | 0.40 | 52.11 |
| **8** | 30 | 98 | 126.14 | 6.78 | 1.28 | 262.04 |
| **9** | 30 | 98 | 100.06 | 6.02 | 1.02 | 184.60 |
| **10** | 30 | 98 | 63.20 | 10.75 | 0.64 | 208.17 |
| **11** | 30 | 98 | 55.87 | 7.16 | 0.57 | 122.62 |
| **12** | 30 | 98 | 230.68 | 3.21 | 2.35 | 227.36 |
| **13** | 30 | 98 | 11.61 | 11.37 | 0.11 | 40.47 |
| **14** | 30 | 98 | 109.30 | 9.21 | 1.11 | 308.38 |
| **15** | 30 | 98 | 110.50 | 8.41 | 1.12 | 284.68 |
| **16** | 30 | 98 | 237.76 | 3.02 | 2.42 | 220.53 |
| **17** | 30 | 98 | 30.91 | 9.44 | 0.31 | 89.42 |
| **18** | 30 | 98 | 126.35 | 9.21 | 1.28 | 356.49 |
| **19** | 30 | 98 | 82.60 | 5.62 | 0.84 | 142.35 |
| **20** | 30 | 98 | 30.07 | 8.12 | 0.30 | 74.82 |
| **21** | 30 | 98 | 73.65 | 6.54 | 0.75 | 147.65 |
| **22** | 30 | 98 | 34.79 | 8.10 | 0.35 | 86.37 |
| **23** | 30 | 98 | 135.76 | 6.62 | 1.38 | 275.52 |
| **24** | 30 | 98 | 115.84 | 4.28 | 1.18 | 152.13 |
| **25** | 30 | 98 | 105.14 | 7.20 | 1.07 | 232.04 |
| **26** | 30 | 98 | 91.89 | 2.16 | 0.93 | 61.04 |
| **27** | 30 | 98 | 41.12 | 4.64 | 0.41 | 58.54 |
| **28** | 30 | 98 | 221.10 | 3.31 | 2.25 | 224.71 |
| **29** | 30 | 98 | 83.54 | 5.84 | 0.85 | 149.53 |
| **30** | 30 | 98 | 51.48 | 4.09 | 0.52 | 64.61 |
| **31** | 30 | 98 | 20.08 | 5.91 | 0.20 | 36.38 |
| **32** | 30 | 98 | 95.27 | 4.55 | 0.97 | 132.99 |
| **33** | 30 | 98 | 52.79 | 6.54 | 0.53 | 105.84 |
| **34** | 30 | 98 | 65.96 | 5.76 | 0.67 | 116.52 |
| **35** | 30 | 98 | 156.17 | 5.95 | 1.59 | 284.77 |
| **36** | 30 | 98 | 92.30 | 9.47 | 0.94 | 267.80 |
| **37** | 30 | 98 | 97.91 | 5.29 | 0.99 | 158.84 |
| **38** | 30 | 98 | 116.44 | 6.68 | 1.18 | 238.38 |
| **39** | 30 | 98 | 117.63 | 8.22 | 1.20 | 296.16 |
| **40** | 30 | 98 | 29.51 | 4.59 | 0.30 | 41.56 |
| **41** | 30 | 98 | 112.04 | 6.38 | 1.14 | 218.84 |
| **42** | 30 | 98 | 114.97 | 7.16 | 1.17 | 252.31 |
| **43** | 30 | 98 | 80.92 | 9.33 | 0.82 | 231.33 |
| **44** | 30 | 98 | 46.47 | 7.83 | 0.47 | 111.48 |
| **45** | 30 | 98 | 94.72 | 4.41 | 0.96 | 128.16 |
| **46** | 30 | 98 | 110.52 | 3.73 | 1.12 | 126.53 |
| **47** | 30 | 98 | 29.63 | 5.76 | 0.30 | 52.34 |
| **48** | 30 | 98 | 215.92 | 4.28 | 2.20 | 283.55 |
| **49** | 30 | 98 | 60.73 | 5.84 | 0.61 | 108.69 |
| **50** | 30 | 98 | 558.59 | 1.22 | 5.69 | 210.26 |
| **51** | 30 | 98 | 91.00 | 5.91 | 0.92 | 164.81 |
| **52** | 30 | 98 | 98.77 | 8.44 | 1.00 | 255.34 |
| **53** | 30 | 98 | 28.86 | 8.32 | 0.29 | 73.59 |
| **54** | 30 | 98 | 129.28 | 4.59 | 1.31 | 182.05 |
| **55** | 30 | 98 | 55.05 | 5.55 | 0.56 | 93.71 |
| **56** | 30 | 98 | 71.20 | 7.64 | 0.72 | 166.62 |
| **57** | 30 | 98 | 97.90 | 5.31 | 0.99 | 159.43 |
| **58** | 30 | 98 | 41.50 | 3.93 | 0.42 | 50.06 |
| **59** | 30 | 98 | 72.61 | 3.40 | 0.74 | 75.80 |
| **60** | 30 | 98 | 66.68 | 5.95 | 0.68 | 121.59 |
| **61** | 30 | 98 | 85.64 | 4.51 | 0.87 | 118.50 |
| **62** | 30 | 98 | 77.24 | 4.62 | 0.78 | 109.47 |
| **63** | 30 | 98 | 100.86 | 6.63 | 1.02 | 204.87 |
| **64** | 30 | 98 | 91.57 | 5.45 | 0.93 | 153.05 |
| **65** | 30 | 98 | 23.95 | 6.54 | 0.24 | 48.02 |
| **66** | 30 | 98 | 101.01 | 4.61 | 1.03 | 142.86 |
| **67** | 30 | 98 | 194.34 | 4.33 | 1.98 | 258.18 |
| **68** | 30 | 98 | 161.43 | 3.53 | 1.64 | 174.93 |
| **69** | 30 | 98 | 27.97 | 4.98 | 0.28 | 42.73 |
| **70** | 30 | 98 | 217.27 | 2.53 | 2.21 | 168.93 |
| **71** | 30 | 98 | 170.63 | 3.55 | 1.74 | 185.93 |
| **72** | 30 | 98 | 150.76 | 3.86 | 1.53 | 178.60 |
| **73** | 30 | 98 | 102.29 | 8.41 | 1.04 | 263.53 |
| **74** | 30 | 98 | 79.37 | 10.36 | 0.80 | 251.83 |
| **75** | 30 | 98 | 57.06 | 3.58 | 0.58 | 62.71 |
| **76** | 30 | 98 | 61.33 | 10.36 | 0.62 | 194.60 |
| **77** | 30 | 98 | 32.26 | 6.67 | 0.32 | 65.93 |
| **78** | 30 | 98 | 77.36 | 6.92 | 0.78 | 164.02 |
| **79** | 30 | 98 | 81.70 | 5.81 | 0.83 | 145.51 |
| **80** | 30 | 98 | 97.87 | 8.10 | 0.99 | 242.94 |
| **81** | 30 | 98 | 133.95 | 6.41 | 1.36 | 263.04 |
| **82** | 30 | 98 | 106.18 | 4.77 | 1.08 | 155.37 |
| **83** | 30 | 98 | 120.99 | 6.54 | 1.23 | 242.55 |
| **84** | 30 | 98 | 136.70 | 4.28 | 1.39 | 179.51 |
| **85** | 30 | 98 | 75.50 | 5.84 | 0.77 | 135.14 |
| **86** | 30 | 98 | 56.38 | 7.16 | 0.57 | 123.73 |
| **87** | 30 | 98 | 95.24 | 9.44 | 0.97 | 275.48 |
| **88** | 30 | 98 | 101.10 | 4.32 | 1.03 | 134.01 |
| **89** | 30 | 98 | 56.15 | 4.23 | 0.57 | 72.88 |
| **90** | 30 | 98 | 32.50 | 8.47 | 0.33 | 84.37 |
| **Averag.** | 30 | 98 | 98.46 | 6.03 | 1.00 | 162.69 |
